# Supplementary material for: Investigating the optimal handling of uncertain pregnancy episodes in the CPRD GOLD Pregnancy Register: a methodological study using UK primary care data
Source: BMJ Open. 2022 Feb 22;12(2):e055773. doi: 10.1136/bmjopen-2021-055773 (PMC8867343; doi:10.1136/bmjopen-2021-055773)
Supplement: Supplementary data [file bmjopen-2021-055773supp001.pdf]

# Appendix

## Appendix 1: Key CPRD GOLD variables

| *Column name* | *Field name* | *Description* |
|---|---|---|
| Last Collection Date | lcd | Date of the last collection for the practice |
| Up to Standard Date | uts | Date at which the practice data is deemed to be of research quality. Derived using a CPRD algorithm that primarily looks at practice death recording and gaps in the data |
| First Registration Date | frd | Date the patient first registered with the practice. |
| Current Registration Date | crd | Date the patient's current period of registration with the practice began. |
| Transfer Out Date | tod | Date the patient transferred out of the practice, if relevant. Empty for patients who have not transferred out |
| Death Date | deathdate | Patient's date of death – derived using a CPRD algorithm |
| Acceptable Patient Flag | accept | Flag to indicate whether the patient has met certain quality standards: 1 = acceptable, 0 = unacceptable |
| Event Date | eventdate | Date associated with the event, as entered by the GP |

| | | |
|---|---|---|
| System Date | sysdate | The date on which information was entered on to the GP software system (generated automatically) |

## Appendix 2: CPRD Pregnancy Register Variables

| *Field name* | *Description* |
|---|---|
| Patid | Encrypted unique patient identifier |
| Pregid | Unique identifier of the pregnancy episode |
| Mblbabies | Number of babies the pregnancy is linked to in the MBL |
| babypatid[1] | Encrypted unique patient identifier (linked baby) |
| babymob | Baby's month of birth as recorded in the baby's medical record |
| babyyob | Baby's year of birth as recorded in the baby's medical record |
| totalpregs | Total number of identified pregnancy episodes (per woman) |
| pregnumber | Pregnancy episode number (per woman) |
| pregstart | Estimated start date of pregnancy |
| firstantenatal | Date of earliest antenatal record within the pregnancy |

**Appendix 3 Example of how a pregnancy may appear in the Register vs GOLD data vs reality**

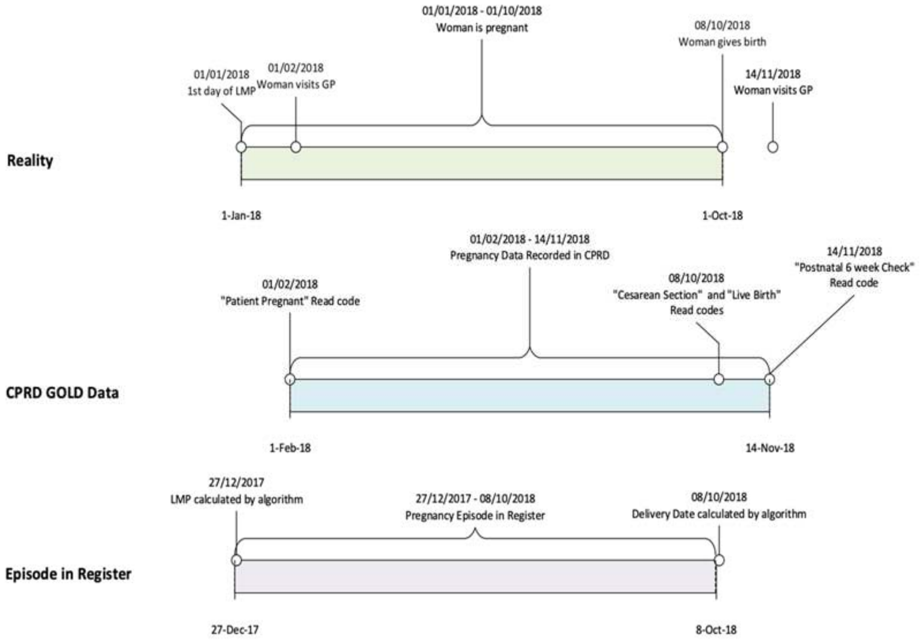

**Appendix 4: ICD codes indicating end of pregnancy**

| | | |
|---|---|---|
| O00 | Ectopic pregnancy | |
| O00.0 | Abdominal pregnancy | |
| O00.1 | Tubal pregnancy | |
| O00.2 | Ovarian pregnancy | |
| O00.8 | Other ectopic pregnancy | |
| O00.9 | Ectopic pregnancy, unspecified | |
| O01 | Hydatidiform mole | |
| O01.0 | Classical hydatidiform mole | |
| O01.1 | Incomplete and partial hydatidiform mole | |
| O01.9 | Hydatidiform mole, unspecified | |
| O02 | Other abnormal products of conception | |
| O02.0 | Blighted ovum and nonhydatidiform mole | |
| O02.1 | Missed abortion | |
| O02.8 | Other specified abnormal products of conception | |
| O02.9 | Abnormal product of conception, unspecified | |
| O03 | Spontaneous abortion | |
| O03.0 | Spontaneous abortion | Incomplete, complicated by genital tract and pelvic infection |
| O03.1 | Spontaneous abortion | Incomplete, complicated by delayed or excessive haemorrhage |
| O03.2 | Spontaneous abortion | Incomplete, complicated by embolism |
| O03.3 | Spontaneous abortion | Incomplete, with other and unspecified complications |
| O03.4 | Spontaneous abortion | Incomplete, without complication |
| O03.5 | Spontaneous abortion | Complete or unspecified, complicated by genital tract and pelvic infection |
| O03.6 | Spontaneous abortion | Complete or unspecified, complicated by delayed or excessive haemorrhage |
| O03.7 | Spontaneous abortion | Complete or unspecified, complicated by embolism |
| O03.8 | Spontaneous abortion | Complete or unspecified, with other and unspecified complications |
| O03.9 | Spontaneous abortion | Complete or unspecified, without complication |

| | | |
|---|---|---|
| O04 | Medical abortion | |
| O04.0 | Medical abortion | Incomplete, complicated by genital tract and pelvic infection |
| O04.1 | Medical abortion | Incomplete, complicated by delayed or excessive haemorrhage |
| O04.2 | Medical abortion | Incomplete, complicated by embolism |
| O04.3 | Medical abortion | Incomplete, with other and unspecified complications |
| O04.4 | Medical abortion | Incomplete, without complication |
| O04.5 | Medical abortion | Complete or unspecified, complicated by genital tract and pelvic infection |
| O04.6 | Medical abortion | Complete or unspecified, complicated by delayed or excessive haemorrhage |
| O04.7 | Medical abortion | Complete or unspecified, complicated by embolism |
| O04.8 | Medical abortion | Complete or unspecified, with other and unspecified complications |
| O04.9 | Medical abortion | Complete or unspecified, without complication |
| O05 | Other abortion | |
| O05.0 | Other abortion | Incomplete, complicated by genital tract and pelvic infection |
| O05.1 | Other abortion | Incomplete, complicated by delayed or excessive haemorrhage |
| O05.2 | Other abortion | Incomplete, complicated by embolism |
| O05.3 | Other abortion | Incomplete, with other and unspecified complications |
| O05.4 | Other abortion | Incomplete, without complication |
| O05.5 | Other abortion | Complete or unspecified, complicated by genital tract and pelvic infection |
| O05.6 | Other abortion | Complete or unspecified, complicated by delayed or excessive haemorrhage |
| O05.7 | Other abortion | Complete or unspecified, complicated by embolism |
| O05.8 | Other abortion | Complete or unspecified, with other and unspecified complications |
| O05.9 | Other abortion | Complete or unspecified, without complication |
| O06 | Unspecified abortion | |
| O06.0 | Unspecified abortion | Incomplete, complicated by genital tract and pelvic infection |
| O06.1 | Unspecified abortion | Incomplete, complicated by delayed or excessive haemorrhage |
| O06.2 | Unspecified abortion | Incomplete, complicated by embolism |
| O06.3 | Unspecified abortion | Incomplete, with other and unspecified complications |

| | | |
|---|---|---|
| O06.4 | Unspecified abortion | Incomplete, without complication |
| O06.5 | Unspecified abortion | Complete or unspecified, complicated by genital tract and pelvic infection |
| O06.6 | Unspecified abortion | Complete or unspecified, complicated by delayed or excessive haemorrhage |
| O06.7 | Unspecified abortion | Complete or unspecified, complicated by embolism |
| O06.8 | Unspecified abortion | Complete or unspecified, with other and unspecified complications |
| O06.9 | Unspecified abortion | Complete or unspecified, without complication |
| O07 | Failed attempted abortion | |
| O07.0 | Failed medical abortion, complicated by genital tract and pelvic infection | |
| O07.1 | Failed medical abortion, complicated by delayed or excessive haemorrhage | |
| O07.2 | Failed medical abortion, complicated by embolism | |
| O07.3 | Failed medical abortion, with other and unspecified complications | |
| O07.4 | Failed medical abortion, without complication | |
| O07.5 | Other and unspecified failed attempted abortion, complicated by genital tract and pelvic infection | |
| O07.6 | Other and unspecified failed attempted abortion, complicated by delayed or excessive haemorrhage | |
| O07.7 | Other and unspecified failed attempted abortion, complicated by embolism | |
| O07.8 | Other and unspecified failed attempted abortion, with other and unspecified complications | |
| O07.9 | Other and unspecified failed attempted abortion, without complication | |
| O08 | Complications following abortion and ectopic and molar pregnancy | |
| O08.0 | Genital tract and pelvic infection following abortion and ectopic and molar pregnancy | |
| O08.1 | Delayed or excessive haemorrhage following abortion and ectopic and molar pregnancy | |
| O08.2 | Embolism following abortion and ectopic and molar pregnancy | |
| O08.3 | Shock following abortion and ectopic and molar pregnancy | |
| O08.4 | Renal failure following abortion and ectopic and molar pregnancy | |
| O08.5 | Metabolic disorders following abortion and ectopic and molar pregnancy | |
| O08.6 | Damage to pelvic organs and tissues following abortion and ectopic and molar pregnancy | |
| O08.7 | Other venous complications following abortion and ectopic and molar pregnancy | |
| O08.8 | Other complications following abortion and ectopic and molar pregnancy | |
| O08.9 | Complication following abortion and ectopic and molar pregnancy, unspecified | |

| | |
|---|---|
| O60.1 | Preterm spontaneous labour with preterm delivery |
| O60.2 | Preterm spontaneous labour with term delivery |
| O62.3 | Precipitate labour |
| O68 | Labour and delivery complicated by fetal stress [distress] |
| O68.0 | Labour and delivery complicated by fetal heart rate anomaly |
| O68.1 | Labour and delivery complicated by meconium in amniotic fluid |
| O68.2 | Labour and delivery complicated by fetal heart rate anomaly with meconium in amniotic fluid |
| O68.3 | Labour and delivery complicated by biochemical evidence of fetal stress |
| O68.8 | Labour and delivery complicated by other evidence of fetal stress |
| O68.9 | Labour and delivery complicated by fetal stress, unspecified |
| O69 | Labour and delivery complicated by umbilical cord complications |
| O69.0 | Labour and delivery complicated by prolapse of cord |
| O69.1 | Labour and delivery complicated by cord around neck, with compression |
| O69.2 | Labour and delivery complicated by other cord entanglement, with compression |
| O69.3 | Labour and delivery complicated by short cord |
| O69.4 | Labour and delivery complicated by vasa praevia |
| O69.5 | Labour and delivery complicated by vascular lesion of cord |
| O69.8 | Labour and delivery complicated by other cord complications |
| O69.9 | Labour and delivery complicated by cord complication, unspecified |

| | |
|---|---|
| O70 | Perineal laceration during delivery |
| O70.0 | First degree perineal laceration during delivery |
| O70.1 | Second degree perineal laceration during delivery |
| O70.2 | Third degree perineal laceration during delivery |
| O70.3 | Fourth degree perineal laceration during delivery |
| O70.9 | Perineal laceration during delivery, unspecified |
| O74 | Complications of anaesthesia during labour and delivery |
| O74.0 | Aspiration pneumonitis due to anaesthesia during labour and delivery |
| O74.1 | Other pulmonary complications of anaesthesia during labour and delivery |
| O74.2 | Cardiac complications of anaesthesia during labour and delivery |
| O74.3 | Central nervous system complications of anaesthesia during labour and delivery |
| O74.4 | Toxic reaction to local anaesthesia during labour and delivery |
| O74.5 | Spinal and epidural anaesthesia-induced headache during labour and delivery |
| O74.6 | Other complications of spinal and epidural anaesthesia during labour and delivery |
| O74.7 | Failed or difficult intubation during labour and delivery |
| O74.8 | Other complications of anaesthesia during labour and delivery |
| O74.9 | Complication of anaesthesia during labour and delivery, unspecified |
| O75 | Other complications of labour and delivery, not elsewhere classified |
| O75.0 | Maternal distress during labour and delivery |

| | |
|---|---|
| O75.1 | Shock during or following labour and delivery |
| O75.5 | Delayed delivery after artificial rupture of membranes |
| O75.6 | Delayed delivery after spontaneous or unspecified rupture of membranes |
| O75.7 | Vaginal delivery following previous caesarean section |
| O75.8 | Other specified complications of labour and delivery |
| O75.9 | Complication of labour and delivery, unspecified |
| O80 | Single spontaneous delivery |
| O80.0 | Spontaneous vertex delivery |
| O80.1 | Spontaneous breech delivery |
| O80.8 | Other single spontaneous delivery |
| O80.9 | Single spontaneous delivery, unspecified |
| O81 | Single delivery by forceps and vacuum extractor |
| O81.0 | Low forceps delivery |
| O81.1 | Mid-cavity forceps delivery |
| O81.3 | Other and unspecified forceps delivery |
| O81.4 | Vacuum extractor delivery |
| O81.5 | Delivery by combination of forceps and vacuum extractor |
| O82 | Single delivery by caesarean section |
| O82.0 | Delivery by elective caesarean section |

| | |
|---|---|
| O82.1 | Delivery by emergency caesarean section |
| O82.2 | Delivery by caesarean hysterectomy |
| O82.8 | Other single delivery by caesarean section |
| O82.9 | Delivery by caesarean section, unspecified |
| O83 | Other assisted single delivery |
| O83.0 | Breech extraction |
| O83.1 | Other assisted breech delivery |
| O83.2 | Other manipulation-assisted delivery |
| O83.4 | Destructive operation for delivery |
| O83.8 | Other specified assisted single delivery |
| O83.9 | Assisted single delivery, unspecified |
| O84 | Multiple delivery |
| O84.0 | Multiple delivery, all spontaneous |
| O84.1 | Multiple delivery, all by forceps and vacuum extractor |
| O84.2 | Multiple delivery, all by caesarean section |
| O84.8 | Other multiple delivery |
| O84.9 | Multiple delivery, unspecified |
| P03 | Fetus and newborn affected by other complications of labour and delivery |
| P03.0 | Fetus and newborn affected by breech delivery and extraction |

| | |
|---|---|
| P03.1 | Fetus and newborn affected by other malpresentation, malposition and disproportion during labour and delivery |
| P03.2 | Fetus and newborn affected by forceps delivery |
| P03.3 | Fetus and newborn affected by delivery by vacuum extractor [ventouse] |
| P03.4 | Fetus and newborn affected by caesarean delivery |
| P03.5 | Fetus and newborn affected by precipitate delivery |
| P03.8 | Fetus and newborn affected by other specified complications of labour and delivery |
| P03.9 | Fetus and newborn affected by complication of labour and delivery, unspecified |
| P04.0 | Fetus and newborn affected by maternal anaesthesia and analgesia in pregnancy, labour and delivery |
| P20.1 | Intrauterine hypoxia first noted during labour and delivery |
| P61.2 | Anaemia of prematurity |
| Z37 | Outcome of delivery |
| Z37.0 | Single live birth |
| Z37.1 | Single stillbirth |
| Z37.2 | Twins, both liveborn |
| Z37.3 | Twins, one liveborn and one stillborn |
| Z37.4 | Twins, both stillborn |
| Z37.5 | Other multiple births, all liveborn |
| Z37.6 | Other multiple births, some liveborn |
| Z37.7 | Other multiple births, all stillborn |

| | |
|---|---|
| Z38 | Liveborn infants according to place of birth |
| Z38.0 | Singleton, born in hospital |
| Z38.1 | Singleton, born outside hospital |
| Z38.2 | Singleton, unspecified as to place of birth |
| Z38.3 | Twin, born in hospital |
| Z38.4 | Twin, born outside hospital |
| Z38.5 | Twin, unspecified as to place of birth |
| Z38.6 | Other multiple, born in hospital |
| Z38.7 | Other multiple, born outside hospital |
| Z38.8 | Other multiple, unspecified as to place of birth |
| Z39.0 | Care and examination immediately after delivery |

**Appendix 5: OPCS codes indicating end of pregnancy**

| OPCS | | |
|---|---|---|
| P141 | INCISION OF INTROITUS OF VAGINA | POSTERIOR EPISIOTOMY AND DIVISION OF LEVATOR ANI MUSCLE |
| P142 | INCISION OF INTROITUS OF VAGINA | POSTERIOR EPISIOTOMY NEC |
| P143 | INCISION OF INTROITUS OF VAGINA | ANTERIOR EPISIOTOMY |
| Q101 | CURETTAGE OF UTERUS | DILATION OF CERVIX UTERI AND CURETTAGE OF PRODUCTS OF CONCEP |
| Q102 | CURETTAGE OF UTERUS | CURETTAGE OF PRODUCTS OF CONCEPTION FROM UTERUS NEC |
| Q111 | OTHER EVACUATION OF CONTENTS OF UTERUS | VACUUM ASPIRATION OF PRODUCTS OF CONCEPTION FROM UTERUS NEC |
| Q112 | OTHER EVACUATION OF CONTENTS OF UTERUS | DILATION OF CERVIX UTERI AND EVACUATION OF PRODUCTS OF CONCE |
| Q113 | OTHER EVACUATION OF CONTENTS OF UTERUS | EVACUATION OF PRODUCTS OF CONCEPTION FROM UTERUS NEC |
| Q115 | OTHER EVACUATION OF CONTENTS OF UTERUS | VACUUM ASPIRATION/PRODUCTS OF CONCEPTION/UTERUS USING RIGID |
| Q116 | OTHER EVACUATION OF CONTENTS OF UTERUS | VACUUM ASPIRATION/PRODUCTS OF CONCEPTION/UTERUS USING FLEXI |
| Q141 | INTRODUCTION OF ABORTIFACIENT INTO UTERINE CAVITY | INTRA-AMNIOTIC INJECTION OF PROSTAGLANDIN |
| Q142 | INTRODUCTION OF ABORTIFACIENT INTO UTERINE CAVITY | INTRA-AMNIOTIC INJECTION OF ABORTIFACIENT NEC |
| Q143 | INTRODUCTION OF ABORTIFACIENT INTO UTERINE CAVITY | EXTRA-AMNIOTIC INJECTION OF PROSTAGLANDIN |
| Q144 | INTRODUCTION OF ABORTIFACIENT INTO UTERINE CAVITY | EXTRA-AMNIOTIC INJECTION OF ABORTIFACIENT NEC |
| Q145 | INTRODUCTION OF ABORTIFACIENT INTO UTERINE CAVITY | INSERTION OF PROSTAGLANDIN PESSARY |

| | | |
|---|---|---|
| Q146 | INTRODUCTION OF ABORTIFACIENT INTO UTERINE CAVITY | INSERTION OF ABORTIFACIENT PESSARY NEC |
| Q148 | INTRODUCTION OF ABORTIFACIENT INTO UTERINE CAVITY | OTHER SPECIFIED |
| Q149 | INTRODUCTION OF ABORTIFACIENT INTO UTERINE CAVITY | UNSPECIFIED |
| R031 | SELECTIVE DESTRUCTION OF FETUS | EARLY SELECTIVE FETICIDE |
| R032 | SELECTIVE DESTRUCTION OF FETUS | LATE SELECTIVE FETICIDE |
| R038 | SELECTIVE DESTRUCTION OF FETUS | OTHER SPECIFIED |
| R039 | SELECTIVE DESTRUCTION OF FETUS | UNSPECIFIED |
| R141 | SURGICAL INDUCTION OF LABOUR | FOREWATER RUPTURE OF AMNIOTIC MEMBRANE |
| R142 | SURGICAL INDUCTION OF LABOUR | HINDWATER RUPTURE OF AMNIOTIC MEMBRANE |
| R148 | SURGICAL INDUCTION OF LABOUR | OTHER SPECIFIED |
| R149 | SURGICAL INDUCTION OF LABOUR | UNSPECIFIED |
| R151 | OTHER INDUCTION OF LABOUR | MEDICAL INDUCTION OF LABOUR |
| R158 | OTHER INDUCTION OF LABOUR | OTHER SPECIFIED |
| R159 | OTHER INDUCTION OF LABOUR | UNSPECIFIED |
| R171 | ELECTIVE CAESAREAN DELIVERY | ELECTIVE UPPER UTERINE SEGMENT CAESAREAN DELIVERY |
| R172 | ELECTIVE CAESAREAN DELIVERY | ELECTIVE LOWER UTERINE SEGMENT CAESAREAN DELIVERY |
| R178 | ELECTIVE CAESAREAN DELIVERY | OTHER SPECIFIED |
| R179 | ELECTIVE CAESAREAN DELIVERY | UNSPECIFIED |
| R181 | OTHER CAESAREAN DELIVERY | UPPER UTERINE SEGMENT CAESAREAN DELIVERY NEC |
| R182 | OTHER CAESAREAN DELIVERY | LOWER UTERINE SEGMENT CAESAREAN DELIVERY NEC |
| R188 | OTHER CAESAREAN DELIVERY | OTHER SPECIFIED |
| R189 | OTHER CAESAREAN DELIVERY | UNSPECIFIED |

| | | |
|---|---|---|
| R191 | BREECH EXTRACTION DELIVERY | BREECH EXTRACTION DELIVERY WITH VERSION |
| R198 | BREECH EXTRACTION DELIVERY | OTHER SPECIFIED |
| R199 | BREECH EXTRACTION DELIVERY | UNSPECIFIED |
| R201 | OTHER BREECH DELIVERY | SPONTANEOUS BREECH DELIVERY |
| R202 | OTHER BREECH DELIVERY | ASSISTED BREECH DELIVERY |
| R208 | OTHER BREECH DELIVERY | OTHER SPECIFIED |
| R209 | OTHER BREECH DELIVERY | UNSPECIFIED |
| R211 | FORCEPS CEPHALIC DELIVERY | HIGH FORCEPS CEPHALIC DELIVERY WITH ROTATION |
| R212 | FORCEPS CEPHALIC DELIVERY | HIGH FORCEPS CEPHALIC DELIVERY NEC |
| R213 | FORCEPS CEPHALIC DELIVERY | MID FORCEPS CEPHALIC DELIVERY WITH ROTATION |
| R214 | FORCEPS CEPHALIC DELIVERY | MID FORCEPS CEPHALIC DELIVERY NEC |
| R215 | FORCEPS CEPHALIC DELIVERY | LOW FORCEPS CEPHALIC DELIVERY |
| R218 | FORCEPS CEPHALIC DELIVERY | OTHER SPECIFIED |
| R219 | FORCEPS CEPHALIC DELIVERY | UNSPECIFIED |
| R221 | VACUUM DELIVERY | HIGH VACUUM DELIVERY |
| R222 | VACUUM DELIVERY | LOW VACUUM DELIVERY |
| R223 | VACUUM DELIVERY | VACUUM DELIVERY BEFORE FULL DILATION OF CERVIX |
| R228 | VACUUM DELIVERY | OTHER SPECIFIED |
| R229 | VACUUM DELIVERY | UNSPECIFIED |
| R231 | CEPHALIC VAGINAL DELIVERY WITH ABNORMAL PRESENTATION OF | MANIPULATIVE CEPHALIC VAGINAL DELIVERY WITH ABNORMAL PRESENT |
| R232 | CEPHALIC VAGINAL DELIVERY WITH ABNORMAL PRESENTATION OF | NON-MANIPULATIVE CEPHALIC VAGINAL DELIVERY WITH ABNORMAL PRE |
| R238 | CEPHALIC VAGINAL DELIVERY WITH ABNORMAL PRESENTATION OF | OTHER SPECIFIED |

| | | |
|---|---|---|
| R239 | CEPHALIC VAGINAL DELIVERY WITH ABNORMAL PRESENTATION OF | UNSPECIFIED |
| R249 | NORMAL DELIVERY | ALL |
| R251 | OTHER METHODS OF DELIVERY | CAESAREAN HYSTERECTOMY |
| R252 | OTHER METHODS OF DELIVERY | DESTRUCTIVE OPERATION TO FACILITATE DELIVERY |
| R258 | OTHER METHODS OF DELIVERY | OTHER SPECIFIED |
| R259 | OTHER METHODS OF DELIVERY | UNSPECIFIED |
| R271 | OTHER OPERATIONS TO FACILITATE DELIVERY | EPISIOTOMY TO FACILITATE DELIVERY |
| R278 | OTHER OPERATIONS TO FACILITATE DELIVERY | OTHER SPECIFIED |
| R279 | OTHER OPERATIONS TO FACILITATE DELIVERY | UNSPECIFIED |
| R281 | INSTRUMENTAL REMOVAL/PRODUCTS/CONCEPTION FROM DEL.UTERU | CURETTAGE OF DELIVERED UTERUS |
| R288 | INSTRUMENTAL REMOVAL/PRODUCTS/CONCEPTION FROM DEL.UTERU | OTHER SPECIFIED |
| R289 | INSTRUMENTAL REMOVAL/PRODUCTS/CONCEPTION FROM DEL.UTERU | UNSPECIFIED |
| R291 | MANUAL REMOVAL/PRODUCTS/CONCEPTION FROM DELIVERED UTERU | MANUAL REMOVAL OF PLACENTA FROM DELIVERED UTERUS |
| R298 | MANUAL REMOVAL/PRODUCTS/CONCEPTION FROM DELIVERED UTERU | OTHER SPECIFIED |
| R299 | MANUAL REMOVAL/PRODUCTS/CONCEPTION FROM DELIVERED UTERU | UNSPECIFIED |

| | | |
|---|---|---|
| R301 | OTHER OPERATIONS ON DELIVERED UTERUS | REPOSITIONING OF INVERTED DELIVERED UTERUS |
| R302 | OTHER OPERATIONS ON DELIVERED UTERUS | EXPRESSION OF PLACENTA |
| R303 | OTHER OPERATIONS ON DELIVERED UTERUS | INSTRUMENTAL EXPLORATION OF DELIVERED UTERUS NEC |
| R304 | OTHER OPERATIONS ON DELIVERED UTERUS | MANUAL EXPLORATION OF DELIVERED UTERUS NEC |
| R308 | OTHER OPERATIONS ON DELIVERED UTERUS | OTHER SPECIFIED |
| R309 | OTHER OPERATIONS ON DELIVERED UTERUS | UNSPECIFIED |
| R321 | REPAIR OF OBSTETRIC LACERATION | REPAIR OF OBSTETRIC LACERATION OF UTERUS OR CERVIX UTERI |
| R322 | REPAIR OF OBSTETRIC LACERATION | REPAIR OF OBSTETRIC LACERATION OF PERINEUM AND SPHINCTER |
| R323 | REPAIR OF OBSTETRIC LACERATION | REPAIR OF OBSTETRIC LACERATION OF VAGINA AND FLOOR OF PELVIS |
| R324 | REPAIR OF OBSTETRIC LACERATION | REPAIR OF MINOR OBSTETRIC LACERATION |
| R325 | REPAIR OF OBSTETRIC LACERATION | REPAIR OBSTETRIC LACERATION PERINEUM SPHINCTER MUCOSA ANUS |
| R328 | REPAIR OF OBSTETRIC LACERATION | OTHER SPECIFIED |
| R329 | REPAIR OF OBSTETRIC LACERATION | UNSPECIFIED |

**Appendix 6: HES Maternity Values to indicate delivery**

| *Variable* | *Definition* | *Acceptable values* |
|---|---|---|
| numbaby | Number of babies delivered | 1-4 |
| delmeth | Method used to deliver a baby that is a registrable birth | 0-9 |
| delplac | Actual type of delivery place | 0-8 |
| delprean | Anaesthetic or analgesic administered before and during labour and delivery | 1-7 |
| delposan | Anaesthetic or analgesic administered after delivery | 1-7 |
| neodur | Baby's age in days | >=1 |
| neocare | Neonatal level of care | 0-3 |
| postdur | Postnatal days of stay | >=1 |

**Appendix 7: Pregnancy Read codes identified as likely to be recorded as useful pregnancy history**

| *medcode* | read_oxmis_code | read_oxmis_term |
|---|---|---|
| *164* | 635..13 | Premature baby |
| *165* | L04..11 | Miscarriage |
| *255* | L05..12 | Termination of pregnancy |
| *364* | 7F13111 | Lower uterine segment caesarean section (LSCS) NEC |
| *618* | L398400 | Delivery by emergency caesarean section |
| *683* | Q420.00 | Haemolytic disease due to rhesus isoimmunisation |
| *720* | L398.00 | Caesarean delivery |
| *740* | 7F12.00 | Elective caesarean delivery |
| *863* | L398200 | Caesarean section - pregnancy at term |
| *974* | Q4z..15 | Stillbirth NEC |
| *1413* | L264.00 | Intrauterine death |
| *1492* | L36..00 | Postpartum haemorrhage (PPH) |
| *1744* | L03..00 | Ectopic pregnancy |
| *2240* | Q4z..12 | Neonatal death |
| *2638* | L1...00 | Pregnancy complications |
| *2639* | E204.11 | Postnatal depression |
| *2664* | L180900 | Gestational diabetes mellitus |
| *2787* | L11..11 | Antepartum haemorrhage |
| *2923* | 62T1.00 | Puerperal depression |
| *2924* | 7E06600 | Hysterotomy and termination of pregnancy |
| *3029* | L166500 | Infections of kidney in pregnancy |
| *3085* | 7F12z00 | Elective caesarean delivery NOS |
| *3327* | L13..11 | Hyperemesis gravidarum |
| *3874* | L031200 | Tubal abortion |
| *4367* | L362.00 | Secondary and delayed postpartum haemorrhage |
| *4530* | L00..00 | Hydatidiform mole |
| *4607* | L414.00 | Postnatal deep vein thrombosis |

| | | |
|---|---|---|
| *4638* | 7F13.00 | Other caesarean delivery |
| *4786* | L213200 | Multiple delivery, all by caesarean section |
| *4979* | Eu53012 | [X]Postpartum depression NOS |
| *5113* | L39y411 | Postnatal vaginal discomfort |
| *5464* | L11y100 | Other antepartum haemorrhage - delivered |
| *7174* | L43..00 | Obstetric pulmonary embolism |
| *7670* | L398z00 | Caesarean delivery NOS |
| *7916* | Z254500 | Delivered by caesarean section - pregnancy at term |
| *8147* | L264.11 | Fetal death in utero |
| *8295* | Q48D100 | [X]Macerated stillbirth |
| *8446* | L180811 | Gestational diabetes mellitus |
| *8776* | Q48D.00 | [X] Stillbirth |
| *8906* | ZV27.12 | [V]Stillbirth |
| *9067* | L125.00 | Severe pre-eclampsia |
| *9668* | 7F12100 | Elective lower uterine segment caesarean delivery |
| *9800* | L398300 | Delivery by elective caesarean section |
| *10049* | 7F12111 | Elective lower uterine segment caesarean section (LSCS) |
| *10278* | L180800 | Diabetes mellitus arising in pregnancy |
| *11359* | L180.00 | Diabetes mellitus during pregnancy/childbirth/puerperium |
| *11947* | L181500 | Postpartum thyroiditis |
| *11986* | 7E13300 | Excision of ruptured ectopic tubal pregnancy |
| *12090* | L126.00 | Eclampsia |
| *12118* | 7F13300 | Emergency caesarean section |
| *12320* | L09..11 | Complications following abortion/ectopic/molar pregnancies |
| *13307* | Eu53011 | [X]Postnatal depression NOS |
| *13584* | 3885 | Edinburgh postnatal depression scale |
| *15061* | L13..12 | Hyperemesis of pregnancy |
| *15514* | 7F13000 | Upper uterine segment caesarean delivery NEC |
| *15533* | L451400 | Obstetric breast abscess with postnatal complication |

| | | |
|---|---|---|
| *16250* | L414.12 | Phlegmasia alba dolens - obstetric |
| *16281* | L45z400 | Obstetric breast infection NOS with postnatal complication |
| *16321* | L360.00 | Third-stage postpartum haemorrhage |
| *17614* | Eu53111 | [X]Puerperal psychosis NOS |
| *17744* | 7F13100 | Lower uterine segment caesarean delivery NEC |
| *18258* | L167.00 | Liver disorder in pregnancy |
| *18369* | ZV27100 | [V]Single stillbirth |
| *18702* | 6G00.00 | Postnatal depression counselling |
| *18770* | Q20yz13 | Renal injury due to birth trauma |
| *18830* | L414.11 | DVT - deep venous thrombosis, postnatal |
| *20152* | L090y00 | Sepsis NOS following abortion/ectopic/molar pregnancy |
| *20165* | L363.00 | Postpartum coagulation defects |
| *20307* | L091.00 | Delayed/excessive haemorrhage following abortive pregnancy |
| *20573* | Q48D000 | [X]Fresh stillbirth |
| *22775* | L11y.00 | Other antepartum haemorrhage |
| *23015* | 6334 | Twins - 1 still + 1 live born |
| *23588* | L414200 | Postnatal deep vein thrombosis with postnatal complication |
| *23642* | Eu53z00 | [X]Puerperal mental disorder, unspecified |
| *24089* | L356z00 | Obstetric damage to pelvic joints and ligaments NOS |
| *24927* | Eu53.00 | [X]Mental and behav disorders assoc with the puerperium NEC |
| *24951* | L18C.00 | Endocrine nutrition+metab dis complic pregn,childbirth+puerp |
| *25028* | L09z.00 | Complication NOS following abortion/ectopic/molar pregnancy |
| *25415* | Q411.00 | Perinatal intraventricular haemorrhage |
| *28364* | Q420.12 | Rhesus isoimmunisation of the newborn |
| *28861* | L398500 | Delivery by caesarean hysterectomy |
| *29155* | 7F1A000 | Caesarean hysterectomy |

| | | |
|---|---|---|
| *31203* | 6332 | Single stillbirth |
| *31857* | Q204.00 | Spine or spinal cord injury due to birth trauma |
| *32950* | L03y100 | Cornual pregnancy |
| *33477* | L398100 | Caesarean delivery - delivered |
| *33724* | L03z.00 | Ectopic pregnancy NOS |
| *34136* | L120z00 | Benign essential hypertension in preg/childb/puerp NOS |
| *34173* | L12B.00 | Proteinuric hypertension of pregnancy |
| *34299* | L240.00 | Congenital abnormality of uterus in preg/childbirth/puerp |
| *34502* | 6335 | Twins - both still born |
| *34639* | L180100 | Diabetes mellitus during pregnancy - baby delivered |
| *34868* | L4...00 | Complications of the puerperium |
| *35190* | 7F13z00 | Other caesarean delivery NOS |
| *35309* | 6755 | Post miscarriage counselling |
| *36421* | L167z00 | Liver disorder in pregnancy NOS |
| *37280* | L36z.00 | Postpartum haemorrhage NOS |
| *39117* | L126500 | Eclampsia in pregnancy |
| *40224* | Eu53000 | [X]Mild mental/behav disorder assoc with the puerperium NEC |
| *40500* | Eu53100 | [X]Severe mental and behav disorder assoc wth puerperium NEC |
| *40730* | L125z00 | Severe pre-eclampsia NOS |
| *42088* | L125100 | Severe pre-eclampsia - delivered |
| *42598* | L175.00 | Maternal rubella in pregnancy, childbirth and the puerperium |
| *44494* | L441z00 | Caesarean wound disruption NOS |
| *45806* | L070x00 | Unspecified abortion with complication NOS |
| *46756* | L184.00 | Mental disorders in pregnancy, childbirth and the puerperium |
| *47227* | ZV27300 | [V]Twins, one live born and one stillborn |
| *47542* | L362200 | Secondary postpartum haemorrhage with postnatal problem |

| | | |
|---|---|---|
| *47546* | 7F12y00 | Other specified elective caesarean delivery |
| *47607* | L440.11 | CVA - cerebrovascular accident in the puerperium |
| *47686* | L181.00 | Thyroid dysfunction in pregnancy/childbirth/puerperium |
| *47741* | L127000 | Pre-eclampsia or eclampsia with hypertension unspecified |
| *47863* | Lyu5200 | [X]Other single delivery by caesarean section |
| *48500* | Q49..00 | Cardiovascular disorders originating in the perinatal period |
| *49363* | Q200100 | Subdural haemorrhage unspecified, due to birth trauma |
| *50093* | L093000 | Oliguria following abortive pregnancy |
| *52875* | L398000 | Caesarean delivery unspecified |
| *52967* | Lyu0B00 | [X]Complic following abortion & ectopic & molar preg, unspec |
| *53141* | L241.00 | Tumour of uterine body in pregnancy/childbirth/puerperium |
| *54652* | L362z00 | Secondary and delayed postpartum haemorrhage NOS |
| *55304* | L131z00 | Hyperemesis gravidarum with metabolic disturbance NOS |
| *56279* | L440.12 | Stroke in the puerperium |
| *57236* | L400200 | Puerperal endometritis with postnatal complication |
| *58156* | L03y.00 | Other ectopic pregnancy |
| *58982* | L186.00 | Other cardiovascular diseases in pregnancy/childbirth/puerp |
| *61204* | L414z00 | Postnatal deep vein thrombosis NOS |
| *61578* | L441000 | Caesarean wound disruption unspecified |
| *62052* | L092500 | Uterus damage following abortive pregnancy |
| *62358* | L167000 | Liver disorder in pregnancy unspecified |
| *62919* | L125200 | Severe pre-eclampsia - delivered with postnatal complication |
| *63277* | L393.00 | Acute renal failure following labour and delivery |
| *64127* | L121000 | Renal hypertension in pregnancy/childbirth/puerp unspecified |
| *64384* | L180z00 | Diabetes mellitus in pregnancy/childbirth/puerperium NOS |
| *66213* | Q20yz12 | Kidney injury due to birth trauma |

| | | |
|---|---|---|
| *66594* | L186.11 | Heart disease during pregnancy |
| *67006* | L096400 | Pulmonary embolism following abortive pregnancy |
| *68319* | L351300 | Rupture of uterus during/after labour with postnatal problem |
| *70891* | L126400 | Eclampsia with postnatal complication |
| *71314* | L093.00 | Renal failure following abortive pregnancy |
| *71717* | L121100 | Renal hypertension in pregnancy/childbirth/puerp - delivered |
| *72215* | L241z00 | Uterine body tumour in pregnancy/childbirth/puerperium NOS |
| *72230* | L241100 | Tumour of uterine body - baby delivered |
| *72458* | L393000 | Post-delivery acute renal failure unspecified |
| *72513* | 7F13200 | Extraperitoneal caesarean section |
| *73407* | L261200 | Rhesus isoimmunisation with antenatal problem |
| *73617* | L261000 | Rhesus isoimmunisation unspecified |
| *73647* | L188000 | Abnormal GTT - unspec whether during pregnancy/puerperium |
| *86756* | Qyu3600 | [X]Other chronic resp diseases originating/perinatal period |
| *93710* | Q317y00 | Other specified perinatal chronic respiratory disease |
| *94718* | L121z00 | Renal hypertension in pregnancy/childbirth/puerperium NOS |
| *97367* | L43z100 | Obstetric pulmonary embolism NOS - delivered |
| *99188* | L173.00 | Maternal tuberculosis in pregnancy/childbirth/puerperium |
| *103465* | Qyu3B00 | [X]Cardiovasc disord origin in the perinat period, unspecif |
| *103677* | Eu32B00 | [X]Antenatal depression |
| *110868* | L181000 | Thyroid dysfunction - unspec whether in pregnancy/puerperium |
| *111574* | L114z00 | Antepartum haemorrhage with trauma NOS |

**Appendix 8: Antenatal Read codes identified as pregnancy advice codes**

| medcode | read_oxmis_code | read_oxmis_term |
|---|---|---|
| 30351 | 67A6.00 | Drugs in pregnancy advice |
| 36903 | 67AZ.00 | Pregnancy advice NOS |
| 102359 | 67AF.00 | Pregnancy advice for patients with epilepsy |
| 107892 | 67Iu.00 | Advice on risk harm to fetus from maternl medictn dur preg |
| 110888 | 67It.00 | Advice on risk harm to mother from maternl medictn dur preg |

| | |
|---|---|
| startsource | Data source used to estimate pregnancy start date: 1 = Imputed[2], 2 = EDD, 3 = LMP, 4 = Gestational age at birth, 5 = Gestational age from antenatal record, 6 = EDC |
| startadj | Flag to indicate whether the pregnancy start date has been adjusted: 0 = Not adjusted, 1 = Due to antenatal records in the preceding 4 weeks, 2 = Due to specific conflicts between the estimated pregnancy duration and records indicating gestational age at birth (live births and stillbirths only), 3 = Both |
| Secondtrim[3] | Estimated start date of second trimester |
| Thirdtrim[3] | Estimated start date of third trimester |
| pregend | Estimated end date of pregnancy. NB: For pregnancies with unknown outcome, the date of the latest antenatal record in the pregnancy episode is provided. |
| endsource | Data source used to estimate pregnancy end date: 1 = Delivery record, 2 = Postnatal record in the mother's medical record, 3 = Discharge date relating to a delivery, 4 = Baby's (month and) year of birth as recorded in the baby's medical record, 5 = Postnatal record in the baby's medical record, 6 = First consultation in the baby's medical record. Only completed for live births and stillbirths. |
| endadj | Flag to indicate whether the pregnancy end date has been adjusted: 0 = Not adjusted, 1 = Due to specific conflicts between the estimated pregnancy duration and records indicating gestational age, 2 = Due to prior adjustments to the start date, 3 = Both. Missing for deliveries based on late pregnancy records[4]. |
| gestdays | Estimated duration of pregnancy episode in days (calculated as pregend minus pregstart) |

| | |
|---|---|
| matage | Mother's age at end of pregnancy (years) |
| outcome | Outcome of pregnancy: 1 = Live birth, 2 = Stillbirth, 3 = 1 and 2, 4 = Miscarriage, 5 = TOP, 6 = Probable TOP, 7 = Ectopic, 8 = Molar, 9 = Blighted ovum, 10 = Unspecified loss, 11 = Delivery based on a third trimester pregnancy record, 12 = Delivery based on a late pregnancy record[4], 13 = Outcome unknown |
| preterm_ev | Flag to indicate evidence of a premature delivery: 1=preterm, 0=no evidence of preterm, 9=not applicable (outcome not a delivery) |
| postterm_ev | Flag to indicate evidence of a post-term delivery: 1=post-term, 0=no evidence of post-term, 9=not applicable (outcome not a delivery) |
| multiple_ev | Flag to indicate evidence of a multiple pregnancy: 1=multiple, 0=no evidence of multiple. Missing for pregnancy losses. |
| conflict | Flag to indicate whether the pregnancy episode overlaps with another episode (within a woman): 1=conflicting, 0= non-conflicting |

1 A single babypatid is provided. For multiple pregnancies resulting in >1 liveborn infant (when mblbabies>1), additional babypatids may be retrieved from the MBL.

2 For "Outcome unknown" pregnancies, the imputed start date is obtained by subtracting 4 weeks from the earliest antenatal record in the episode.

3 The timing of trimesters is estimated using a common convention: first trimester (first day of LMP [pregstart] to 13 completed weeks), second (weeks 14 to 26), and third (week 27 to delivery [pregend]).

4 Late pregnancy records refer to the period up to 3 weeks before delivery, e.g. "Baby overdue".

**Appendix 9: Read codes potentially misclassified as antenatal rather than outcomes**

| medcode | read_oxmis_code | read_oxmis_term |
|---|---|---|
| 424 | L281.00 | Premature rupture of membranes |
| 906 | L100.00 | Threatened abortion |
| 1413 | L264.00 | Intrauterine death |
| 1737 | L02..00 | Missed abortion |
| 1879 | L071.00 | Unspecified abortion incomplete |
| 3004 | L14..11 | Premature labour |
| 6730 | L051.12 | Surgical abortion - incomplete |
| 7114 | L044.00 | Inevitable abortion incomplete |
| 7413 | L041.00 | Spontaneous abortion incomplete |
| 8076 | 8H7W.00 | Refer to TOP counselling |
| 8147 | L264.11 | Fetal death in utero |
| 8173 | L043.00 | Inevitable abortion unspecified |
| 12241 | L02..11 | Missed miscarriage |
| 12337 | L051.00 | Legal abortion incomplete |
| 17625 | L044.11 | Inevitable miscarriage incomp |
| 20621 | ZV25313 | [V]Admission for termination of pregnancy |
| 20809 | L14..00 | Early or threatened labour |
| 20933 | 6776 | Preg. termination counselling |
| 25883 | L071y00 | Unspecified incomplete abortion + no mention of complication |
| 28605 | L051z00 | Incomplete legal abortion NOS |
| 29439 | L041z00 | Incomplete spontaneous abortion NOS |
| 33964 | L0A4.00 | Failed medical abortion, without complication |
| 35184 | L071z00 | Unspecified incomplete abortion NOS |
| 35273 | L097.00 | Readmission for abortive pregnancy (NHS codes) |
| 35701 | L100000 | Threatened abortion unspecified |
| 37831 | L264z00 | Intrauterine death NOS |

| | | |
|---|---|---|
| 39754 | L051.11 | Medal abortion - incomplete |
| 41118 | L08z.00 | Failed attempted abortion NOS |
| 41783 | L041100 | Incomp spontaneous abortion + delayed/excessive haemorrhage |
| 47376 | L0A1.00 | Failed medical abortion complic by genital tract/pelvic infn |
| 47435 | L097200 | Readmission for retained produc of concept, illegal abortion |
| 50903 | L0A2.00 | Failed medical abortion comp by delayed/excessive haem'ge |
| 53201 | ZV25B00 | [V]Admission for administration of abortifacient |
| 59572 | L0A3.00 | Failed medical abortion, complicated by embolism |
| 59789 | L14z.00 | Early or threatened labour NOS |
| 65716 | Q011.00 | Fetus/neonate affected maternal premature rupture membrane |
| 68683 | 7E0B.00 | Introduction of abortifacient into uterine cavity |
| 96418 | L06z.00 | Illegally induced abortion NOS |
| 97391 | L281200 | Premature rupture of membranes with antenatal problem |
| 99205 | 7E0Bz00 | Introduction of abortifacient into uterine cavity NOS |
| 101959 | 7E0B300 | Extraamniotic injection of abortifacient NEC |
| 102362 | 389B.00 | Assessment for termination of pregnancy |
| 102494 | 8Hh3.00 | Self referral to termination of pregnancy service |
| 105048 | 7E0By00 | Introduction of abortifacient into uterine cavity OS |

**Appendix 10: Outcome Groupings**

Pregnancy Outcomes will be grouped together with those pregnancies which would have similar rules applied and combinations of outcome group for each pair will be coded.

| *Group* | *Pregnancy Register codes* | *Group* |
|---|---|---|
| Early Pregnancy Loss | 4, 5, 6, 10, 7, 8, 9 | 1 |
| Delivery | 1, 2, 3, 11, 12 | 2 |
| Unknown Outcome | 13 | 3 |

## Appendix 11: Read Codes identified as likely to only be recorded during current pregnancy

| medcode | read_oxmis_term |
|---|---|
| 30979 | [SO]Fetus |
| 36441 | [V]Amniocentesis to screen for chromosomal anomalies |
| 61455 | [V]Amniotic fluid to screen for alphafetoprotein levels |
| 6298 | [V]Antenatal screening |
| 49665 | [V]Antenatal screening for chromosomal anomalies |
| 35912 | [V]Pregnancy confirmed |
| 43428 | [V]Screening for fetal growth retardation using ultrasonics |
| 103341 | [V]Screening for isoimmunisation |
| 7536 | [V]Screening for malformations using ultrasonics |
| 13167 | A/N 12 weeks examination |
| 13166 | A/N 16 week examination |
| 29364 | A/N 20 week examination |
| 13169 | A/N 24 week examination |
| 26554 | A/N 28 week examination |
| 29627 | A/N 30 week examination |
| 13171 | A/N 32 week examination |
| 13170 | A/N 34 week examination |
| 29727 | A/N 35 week examination |
| 29610 | A/N 36 week examination |
| 26552 | A/N 37 week examination |
| 26553 | A/N 38 week examination |
| 26551 | A/N 39 week examination |
| 29280 | A/N 40 week examination |
| 37029 | A/N 41 week examination |
| 55605 | A/N 42 week examination |
| 3517 | A/N booking examination |
| 13984 | Antenatal ultrasound confirms ectopic pregnancy |
| 12260 | A/N Rh antibody screen |

| | |
|---|---|
| 68089 | A/N Rh antibody screen NOS |
| 70616 | A/N sickle cell screen done |
| 102099 | A/N sickle cell screen NOS |
| 64141 | A/N syphilis screen-blood sent |
| 14086 | A/N U/S scan abnormal |
| 27057 | A/N U/S scan for ? abnormality |
| 64537 | A/N U/S scan for slow growth |
| 37221 | A/N U/S scan normal +? dates |
| 35826 | A/N U/S scan normal += dates |
| 106588 | Antenatal 22 week examination |
| 106923 | Antenatal 25 week examination |
| 106425 | Antenatal 31 week examination |
| 13168 | Antenatal examination NOS |
| 10056 | Antenatal examinations |
| 13416 | Antenatal sickle cell screen |
| 13417 | Antenatal syphilis screen |
| 42326 | Antenatal syphilis screen NOS |
| 13968 | Antenatal ultrasound confirms intra-uterine pregnancy |
| 2029 | Antenatal ultrasound scan |
| 27056 | Antenatal ultrasound scan at 17-22 weeks |
| 39611 | Antenatal ultrasound scan at 22-40 weeks |
| 14084 | Antenatal ultrasound scan at 9-16 weeks |
| 14083 | Antenatal ultrasound scan NOS |
| 14085 | Antenatal ultrasounds scan at 4-8 weeks |
| 12890 | Confirmation of pregnancy |
| 50546 | Dating scan |
| 9462 | Dating/booking US scan |
| 100164 | Detailed structural scan |
| 103741 | Doppler ultrasound scan of middle cerebral artery of fetus |
| 102885 | Doppler ultrasound scan of umbilical artery |
| 95166 | Doppler ultrasound scan of uterine artery |
| 46126 | Double test |
| 13414 | Downs screen - blood test |

| | |
|---|---|
| 38358 | Downs screen blood test abnormal |
| 34508 | Downs screen blood test normal |
| 64832 | Downs screening - blood sent |
| 39173 | Downs screening blood test NOS |
| 103893 | Fetal ascites scan |
| 19720 | Fetal monitoring |
| 19590 | Fetal movements felt |
| 55493 | Fetal movements seen |
| 53420 | Fetal tachycardia |
| 9164 | Fetal U-S scan |
| 31110 | Fundal height equal to dates |
| 25875 | Fundal height high for dates |
| 37039 | Fundal height low for dates |
| 37038 | Girth of pregnant abdomen |
| 91773 | Good baseline variability in fetal heart rate |
| 105992 | Height of uterine fundus |
| 92171 | Mid trimester scan |
| 85992 | Non routine obstetric scan for fetal observations |
| 95875 | Non routine obstetric scan for fetal observations NOS |
| 38846 | Normal fetal heart baseline pattern |
| 13997 | Nuchal scan |
| 95881 | O/E - fetal heart < 40 |
| 101119 | O/E - fetal heart > 200 |
| 68996 | O/E - fetal heart 100-120 |
| 26707 | O/E - fetal heart 120-160 |
| 62903 | O/E - fetal heart 160-180 |
| 62898 | O/E - fetal heart 180-200 |
| 72837 | O/E - fetal heart 40-80 |
| 70856 | O/E - fetal heart 80-100 |
| 7681 | O/E - fetal heart heard |
| 22815 | O/E - fetal movements |
| 25153 | O/E - fetal movements felt |
| 52857 | O/E - fetal movements NOS |
| 53687 | O/E - fetal movements seen |

| | |
|---|---|
| 27801 | O/E - fetal movemnt.diminished |
| 26710 | O/E - fetal presentation |
| 67186 | O/E - fetal presentation NOS |
| 69819 | O/E - fetal station NOS |
| 24701 | O/E - fetus very active |
| 26708 | O/E - fundal size = dates |
| 37049 | O/E - fundus = term size |
| 26705 | O/E - fundus 12-16 week size |
| 37051 | O/E - fundus 16-20 week size |
| 26704 | O/E - fundus 20-24 week size |
| 26709 | O/E - fundus 24-28 week size |
| 30802 | O/E - fundus 28-32 week size |
| 30803 | O/E - fundus 32-34 week size |
| 26703 | O/E - fundus 34-36 week size |
| 26706 | O/E - fundus 36-38 week size |
| 13318 | O/E - fundus size - obstetric |
| 30804 | O/E - gravid uterus size |
| 62897 | O/E - gravid uterus size NOS |
| 37180 | O/E - lie of fetus |
| 29788 | O/E - multiple presentation |
| 63024 | O/E -fetal presentation unsure |
| 37050 | O/E -fundus 38 weeks-term size |
| 49519 | Observation of position of pregnancy |
| 12625 | Obstetric monitoring |
| 44173 | Obstetric X-ray - fetus |
| 56727 | Obstetric X-ray - placenta |
| 85951 | Other non routine obstetric scan NOS |
| 96343 | Other specified routine obstetric scan |
| 13165 | Patient currently pregnant |
| 127 | Patient pregnant |
| 14899 | Patient pregnant NOS |
| 38669 | Placenta U-S scan |
| 9986 | Pregnancy care |
| 4536 | Pregnancy confirmed |

| | |
|---|---|
| 15338 | Pregnancy unplanned ? wanted |
| 14877 | Pregnant - ? planned |
| 30817 | Pregnant - blood test confirms |
| 51298 | Pregnant - on abdom. palpation |
| 20240 | Pregnant - planned |
| 16215 | Pregnant - urine test confirms |
| 35592 | Pregnant - V.E. confirms |
| 10173 | Pregnant abdomen observation |
| 15567 | Pregnant -unplanned-not wanted |
| 107698 | Pregnant uterus displaced laterally |
| 32975 | Pregnant, diaphragm failure |
| 29692 | Pregnant, IUD failure |
| 14994 | Pregnant, sheath failure |
| 11989 | Referral for termination of pregnancy |
| 2278 | Requests pregnancy termination |
| 69815 | Rh screen - 1st preg. sample |
| 29623 | Rh screen - 2nd preg. sample |
| 109416 | Rh screen - 3rd preg. sample |
| 93946 | Rhesus detailed scan |
| 86011 | Routine obstetric scan |
| 85245 | Routine obstetric scan NOS |
| 6095 | Seen in antenatal clinic |
| 29205 | Serum pregnancy test positive |
| 70845 | Sinusoidal pattern of fetal heart |
| 27614 | Triple test |
| 39218 | Ultrasonic doppler for fetal heart sounds |
| 19800 | Ultrasound in obstetric diagn. |
| 12837 | Ultrasound monitoring of early pregnancy |
| 13965 | Ultra-sound scan - obstetric |
| 3030 | Urine pregnancy test positive |
| 2382 | U-S obstetric diagn. scan NOS |
| 29685 | U-S obstetric scan abnormal |
| 4797 | U-S obstetric scan normal |
| 45963 | U-S scan - fetal abnormality |

| | |
|---|---|
| 72159 | U-S scan - fetal cephalometry |
| 42093 | U-S scan - fetal maturity |
| 41919 | U-S scan - fetal presentation |
| 41937 | U-S scan - multiple fetus |
| 35558 | U-S scan - obstetric, diagn. |
| 68858 | U-S scan -placental localisatn |
| 67047 | Viability scan |
| 37147 | Viability US scan |
| 10306 | Weeks pregnant |

**Appendix 12: Outcome Group Combinations**

Within conflicting pairs combinations of outcome groups will be coded as follows:

| *Outcome Group combination* | *Variable Code* |
|---|---|
| 1 1 (Loss- Loss) | 1 |
| 1 2 (Loss- Delivery) | 2 |
| 1 3 (Loss- Unknown) | 3 |
| 2 2 (Delivery- Delivery) | 4 |
| 2 3 (Delivery- Unknown) | 5 |
| 3 3 (Unknown- Unknown) | 6 |

**Appendix 13: Read codes for Antenatal scan**

| medcode | read_oxmis_code | Read term |
|---|---|---|
| 2029 | 62G..00 | Antenatal ultrasound scan |
| 13965 | 584..13 | Ultra-sound scan - obstetric |
| 9462 | 584A.00 | Dating/booking US scan |
| 2382 | 584Z.00 | U-S obstetric diagn. scan NOS |
| 13997 | 584G.00 | Nuchal scan |
| 42093 | 5846 | U-S scan - fetal maturity |
| 37147 | 584B.00 | Viability US scan |
| 4797 | 5842 | U-S obstetric scan normal |
| 27019 | 5841 | U-S obstetric scan requested |
| 9164 | 584..11 | Fetal U-S scan |
| 14083 | 62GZ.00 | Antenatal ultrasound scan NOS |
| 35826 | 62G6.00 | A/N U/S scan normal += dates |
| 14084 | 62GC.00 | Antenatal ultrasound scan at 9-16 weeks |
| 35558 | 584..12 | U-S scan - obstetric, diagn. |
| 50546 | 7F26000 | Dating scan |
| 29012 | 7F27300 | Nuchal translucency scan |
| 27056 | 62GD.00 | Antenatal ultrasound scan at 17-22 weeks |
| 39611 | 62GE.00 | Antenatal ultrasound scan at 22-40 weeks |
| 47415 | 62G5.00 | A/N U/S scan awaited |
| 37220 | 62G2.00 | A/N U/S scan offered |
| 14085 | 62GB.00 | Antenatal ultrasounds scan at 4-8 weeks |
| 29685 | 5843 | U-S obstetric scan abnormal |
| 72159 | 5845 | U-S scan - fetal cephalometry |
| 45963 | 5847 | U-S scan - fetal abnormality |
| 27057 | 62G9.00 | A/N U/S scan for ? abnormality |

| | | |
|---|---|---|
| 41919 | 5849 | U-S scan - fetal presentation |
| 30885 | 62G4.00 | A/N U/S scan wanted |
| 86011 | 7F26.00 | Routine obstetric scan |
| 68858 | 5844 | U-S scan -placental localisatn |
| 67047 | 7F26100 | Viability scan |
| 41937 | 5848 | U-S scan - multiple fetus |
| 14086 | 62G8.00 | A/N U/S scan abnormal |
| 85992 | 7F27.00 | Non routine obstetric scan for fetal observations |
| 37221 | 62G7.00 | A/N U/S scan normal +? dates |
| 38669 | 5844.11 | Placenta U-S scan |
| 78449 | 7F28.00 | Other non routine obstetric scan |
| 100164 | 7F27100 | Detailed structural scan |
| 92171 | 7F26200 | Mid trimester scan |
| 95166 | 7F2A111 | Doppler ultrasound scan of uterine artery |
| 64537 | 62GA.00 | A/N U/S scan for slow growth |
| 47116 | 7F28000 | Placental localisation scan |
| 85245 | 7F26z00 | Routine obstetric scan NOS |
| 102885 | 7F2A011 | Doppler ultrasound scan of umbilical artery |
| 96343 | 7F26y00 | Other specified routine obstetric scan |
| 95875 | 7F27z00 | Non routine obstetric scan for fetal observations NOS |
| 85951 | 7F28z00 | Other non routine obstetric scan NOS |
| 98261 | 7F27y00 | OS non routine obstetric scan for fetal observations |
| 95698 | 7F28y00 | Other specified other non routine obstetric scan |

## Appendix 14: DID Snomed foetal scan codes

| | |
|---|---|
| Dating/booking ultrasound scan (procedure) | 169229007 |
| Fetal anatomy study (procedure) | 271442007 |
| Fetal biophysical profile (procedure) | 21623001 |
| Fetal echocardiography (procedure) | 433235006 |
| Magnetic resonance imaging of multiple pregnancy (procedure) | 450825001 |
| Placental localization (procedure) | 164817009 |
| Ultrasonography of multiple pregnancy for fetal anomaly (procedure) | 445866007 |
| Ultrasonography of multiple pregnancy for fetal nuchal translucency (procedure) | 446810002 |
| Ultrasound scan for amniotic fluid volume (procedure) | 241494004 |
| Ultrasound scan for fetal growth (procedure) | 241493005 |

**Appendix 15: Number of episodes with a suitably timed outcome in linked HES data**

| Dataset in which evidence of a suitably timed pregnancy outcome was found. | N pregnancy episodes where evidence of an outcome was found (% of episodes which were eligible for this linked data source) | N pregnancy episodes which were during current registration and UTS follow up | Total number of pregnancy episodes with recorded outcome missing which were eligible for HES linkage to each source |
|---|---|---|---|
| HES Diagnosis (Part of HES APC) | 24,902 (5.9%) | 16,389 (65.8%) | 424,375 |
| HES Maternity (Part of HES APC) | 163,483 (38.5%) | 109,393 (66.9%) | 424,375 |
| HES Procedures (Part of HES APC) | 201,731 (47.5%) | 133,077 (66.0%) | 424,375 |
| HES Episodes (Part of HES APC) | 185,436 (43.7%) | 122,350 (66.0%) | 424,375 |
| HES Outpatient | 735 (0.2%) | 560 (76.2%) | 311,982 |
| Any HES Source | 211,070 (49.7%) | 139,084 (65.9%) | 424,375 |

**Appendix 16: Numbers of pregnancy episodes with recorded outcome missing which were within practice UTS follow-up and patient's current registration period that were consistent with applied criteria for each scenario**

| Scenario | Description | N pregnancy episodes which meet this scenario (% of total episodes with missing outcome) | N pregnancy episodes which only meet this scenario (% of the total episodes with missing outcome) | N pregnancy episodes with evidence of an outcome in linked HES (% of linkage eligible episodes) |
|---|---|---|---|---|
| Denominator | | 475,664 | 475,664 | 265,264 |
| *Problem 1: The women was pregnant at the time of the database record, but the outcome was not captured in CPRD primary care data.* | | | | |
| Scenario 1a | The pregnancy outcome occurred in hospital or elsewhere and information wasn't fed back to the practice. | 139,084 (29.2%) | 1,825 (0.4%) | 139,084 (52.4%) |
| Scenario 1b | The outcome of the pregnancy is recorded in the primary care data but has no event date associated with it. | 475 (0.1%) | 28 (0.0%) | 113 (0.0%) |
| Scenario 1c | The pregnancy occurred before the patient was registered at the practice or before UTS | - | - | - |

| | | | | |
|---|---|---|---|---|
| *Problem 2: The women was pregnant at the time of the database record, but the pregnancy was still ongoing at the end of available follow up in the database.* | | | | |
| Scenario 2a | The patient transferred out before the putative end of pregnancy | 117,571 (24.7%) | 34,659 (7.3%) | 52,601 (19.8%) |
| Scenario 2b | The last collection date of the practice was before the putative end of pregnancy | 58,698 (12.3%) | 20,122 (4.2%) | 21,702 (8.2%) |
| *Problem 3: The patient was not pregnant at the time of the database record.* | | | | |
| Scenario 3a | Episode is derived from historical pregnancy information recorded in the first few months after the patient joined the practice | 3,875 (0.8%) | 386 (0.1%) | 1,271 (0.5%) |
| Scenario 3b | Patient asks for advice whilst planning a pregnancy | 0 (0.0%) | 0 (0.0%) | 0 (0.0%) |
| *Problem 4: The pregnancy record belongs to another pregnancy episode in the Register.* | | | | |
| Scenario 4a | Delay in recording the outcome of a pregnancy, algorithm calculates LMP too late and uncovers records at the beginning of pregnancy creating this PWO. | 35,255 (7.4%) | 8,265 (1.7%) | 14,402 (5.4%) |

| | | | | |
|---|---|---|---|---|
| Scenario 4b | The LMP is derived from the data and is wrong resulting in early codes being uncovered creating this episode | 17,110 (3.6%) | 3,715 (0.8%) | 6,651 (2.5%) |
| Scenario 4c | The LMP has been shifted backwards uncovering records at the end of the pregnancy | 0 (0.0%) | 0 (0.0%) | 0 (0.0%) |
| Scenario 4d | A code recorded relating to the patient's delivery history is incorrectly identified by the algorithm as a delivery uncovering records at the end. | 219,505 (46.1%) | 109,161 (22.9%) | 65,883 (24.8%) |
| Scenario 4e | The outcome of the pregnancy episode has been misclassified as antenatal | 18,222 (3.8%) | 7,418 (1.6%) | 3,990 (1.5%) |
| Pregnancy Episodes which didn't meet any scenario | These pregnancy episodes did not meet the criteria for any identified scenarios. | 94,769 (19.9%) | 0 (0.0%) | 0 (0.0%) |

**Appendix 17: Numbers of conflicting pregnancy episodes which were within practice UTS follow-up and patient's current registration period that were consistent with applied criteria for each scenario**

| Scenario | Description | N pregnancy pairs (% of total conflicting pregnancy pairs) | N which only fit this scenario (% of the total pairs meeting this scenario) | N of pairs with a linked baby in the MBL (% of the total pairs meeting this scenario) | N pairs with evidence of pregnancy in linked HES |
|---|---|---|---|---|---|
| Denominator | | 144,670 | 144,670 | 144,670 | 93,100 |
| *Problem 1: Both pregnancies are true but one is a current pregnancy and one is a historical pregnancy* | | | | | |
| Scenario 1a | The GP records a past delivery or loss during a current pregnancy with the same outcome resulting in another episode being created | 1,981 (1.4%) | 317 (0.2%) | 1,782 (1.2%) | 1,875 (2.0%) |
| Scenario 1b | A patient has a record relating to a loss recorded during a pregnancy ending in delivery or vice-versa. Conflicting episodes are generated by the algorithm | 31,526 (21.8%) | 15,453 (10.7%) | 8,275 (5.7%) | 11,410 (12.3%) |
| *Problem 2: Both pregnancies are historical* | | | | | |

| | | | | | |
|---|---|---|---|---|---|
| Scenario 2a | A patient has information on historical pregnancies recorded with the current date rather than the actual date. | 12,557 (8.7%) | 0 (0.0%) | 97 (0.1%) | 4,309 (4.6%) |
| *Problem 3: Both pregnancies are true and current but the gestation of the second pregnancy estimated by the algorithm is too long.* | | | | | |
| Scenario 3a | The woman has two losses which are >8weeks and <12weeks apart. | 2,284 (1.6%) | 3 (0.0%) | 0 (0.0%) | 635 (0.7%) |
| Scenario 3b | The woman has two pregnancies close together and the second ends in delivery. If the LMP information is wrong for this pregnancy, then algorithm episodes may overlap. | 13,464 (9.3%) | 2,387 (1.6%) | 1,113 (0.8%) | 4,502 (4.8%) |
| *Problem 4: : The pregnancy is true and current but is split into separate episodes by the rules of the algorithm* | | | | | |
| Scenario 4a | The GP records further information about a pregnancy outcome >25 weeks later for deliveries or >8weeks <12 weeks later for losses. | 2,347 (1.6%) | 183 (0.1%) | 2,155 (1.5%) | 2,255 (2.4%) |

| | | | | | |
|---|---|---|---|---|---|
| Scenario 4b | The GP records further antenatal information after the end of a pregnancy. Conflicting episodes are generated by the algorithm | 27,131 (18.8%) | 25,097 (17.3%) | 11,097 (7.7%) | 11,668 (12.5%) |
| Scenario 4c | The patient has a follow up scan after a pregnancy loss. The scan is recorded in the data as an antenatal scan, a conflicting episode is then generated by the algorithm. | 2,088 (1.4%) | 0 (0.0%) | 0 (0.0%) | 587 (0.6%) |
| Scenario 4d | The GP records information about a pregnancy but no outcome with >6 weeks between records. If the second episode has gestational information the start may be assigned before the start of the first episode. | 9,911 (6.9%) | 9,911 (6.9%) | 0 (0.0%) | 5,079 (5.5%) |
| Scenario 4e | The pregnancy dates have been shifted backwards by the rules of the algorithm leaving uncovered records. Conflicting episodes are generated by the algorithm. | 55,205 (38.2%) | 53,044 (36.7%) | 43,945 (30.4%) | 33,057 (35.5%) |
| None | These pairs of pregnancies did not meet the criteria for any identified scenarios. | 15,650 (10.8%) | - | 8,921 (6.2%) | 8,235 (8.8%) |

**Appendix 18: Number of conflicting episode pairs by outcome combination**

| Outcome Combination | N pairs (% of total conflicting pairs) |
|---|---|
| two losses | 65,826 (26.2%) |
| one loss one delivery | 73,222 (29.2%) |
| one loss one unknown | 62,776 (25.0%) |
| two deliveries | 10,204 (4.1%) |
| one delivery one unknown | 24,303 (9.7%) |
| two unknowns | 14,695 (5.9%) |
| Total Pairs | 251,026 (100%) |
