# Supplementary material for: Investigating the optimal handling of uncertain pregnancy episodes in the CPRD GOLD Pregnancy Register: a methodological study using UK primary care data
Source: BMJ Open. 2022 Feb 22;12(2):e055773. doi: 10.1136/bmjopen-2021-055773 (PMC8867343; doi:10.1136/bmjopen-2021-055773)
Supplement: Author's manuscript [file bmjopen-2021-055773.draft_revisions.pdf]

BMJ Open

BMJ Open is committed to open peer review. As part of this commitment we make the peer review history of every article we publish publicly available.

When an article is published we post the peer reviewers' comments and the authors' responses online. We also post the versions of the paper that were used during peer review. These are the versions that the peer review comments apply to.

The versions of the paper that follow are the versions that were submitted during the peer review process. They are not the versions of record or the final published versions. They should not be cited or distributed as the published version of this manuscript.

BMJ Open is an open access journal and the full, final, typeset and author-corrected version of record of the manuscript is available on our site with no access controls, subscription charges or pay-per-view fees (http://bmjopen.bmj.com).

If you have any questions on BMJ Open's open peer review process please email info.bmjopen@bmj.com

BMJ Open

Investigating the Optimal Handling of Uncertain Pregnancy Episodes in the CPRD Pregnancy Register

Journal:	*BMJ Open*
Manuscript ID	bmjopen-2021-055773
Article Type:	Original research
Date Submitted by the Author:	16-Aug-2021

Complete List of Authors:	Campbell, Jennifer; Medicines and Healthcare Products Regulatory Agency, Clinical Practice Research Datalink; London School of Hygiene & Tropical Medicine, Faculty of Epidemiology and Population Health, Department of Non-communicable Disease Epidemiology<br>Bhaskaran, Krishnan; London School of Hygiene & Tropical Medicine, Faculty of Epidemiology and Population Health, Department of Non-communicable Disease Epidemiology<br>Thomas, Sara; London School of Hygiene & Tropical Medicine, Faculty of Epidemiology and Population Health, Department of Infectious Disease Epidemiology<br>Williams, Rachael; Medicines and Healthcare Products Regulatory Agency, Clinical Practice Research Datalink<br>McDonald, Helen; London School of Hygiene & Tropical Medicine, Faculty of Epidemiology and Population Health, Department of Infectious Disease Epidemiology<br>Minassian, Caroline ; London School of Hygiene & Tropical Medicine, Faculty of Epidemiology and Population Health, Department of Non-communicable Disease Epidemiology

Keywords:	Maternal medicine < OBSTETRICS, PUBLIC HEALTH, EPIDEMIOLOGY

SCHOLARONE™
Manuscripts

I, the Submitting Author has the right to grant and does grant on behalf of all authors of the Work (as defined in the below author licence), an exclusive licence and/or a non-exclusive licence for contributions from authors who are: i) UK Crown employees; ii) where BMJ has agreed a CC-BY licence shall apply, and/or iii) in accordance with the terms applicable for US Federal Government officers or employees acting as part of their official duties; on a worldwide, perpetual, irrevocable, royalty-free basis to BMJ Publishing Group Ltd ("BMJ") its licensees and where the relevant Journal is co-owned by BMJ to the co-owners of the Journal, to publish the Work in this journal and any other BMJ products and to exploit all rights, as set out in our licence.

The Submitting Author accepts and understands that any supply made under these terms is made by BMJ to the Submitting Author unless you are acting as an employee on behalf of your employer or a postgraduate student of an affiliated institution which is paying any applicable article publishing charge ("APC") for Open Access articles. Where the Submitting Author wishes to make the Work available on an Open Access basis (and intends to pay the relevant APC), the terms of reuse of such Open Access shall be governed by a Creative Commons licence – details of these licences and which Creative Commons licence will apply to this Work are set out in our licence referred to above.

Other than as permitted in any relevant BMJ Author's Self Archiving Policies, I confirm this Work has not been accepted for publication elsewhere, is not being considered for publication elsewhere and does not duplicate material already published. I confirm all authors consent to publication of this Work and authorise the granting of this licence.

For peer review only

Investigating the Optimal Handling of Uncertain Pregnancy Episodes in the CPRD Pregnancy Register

Jennifer Campbell [1,2](jennifer.campbell@mhra.gov.uk), Krishnan Bhaskaran[2] (krishnan.bhaskaran@lshtm.ac.uk), Sara Thomas[2] (saralmthomas@gmail.com), Rachael Williams [1] (rachael.williams@mhra.gov.uk), Helen I McDonald[2](helen.mcdonald@lshtm.ac.uk) Caroline Minassian[2](caroline.minassian@lshtm.ac.uk).

1. *Clinical Practice Research Datalink (CPRD)*
2. *London School of Hygiene and Tropical Medicine (LSHTM)*

Correspondence to Jennifer Campbell; jennifer.campbell@mhra.gov.uk

Keywords

Pregnancy; Electronic Health Records; United Kingdom; Pregnancy Outcome; Pharmacoepidemiology

Abstract (300 words)

Objectives:

To investigate why episodes of pregnancy identified from electronic health records may be incomplete or conflicting (overlapping) and provide guidance on how to handle them.

Setting:

Pregnancy Register generated from the Clinical Practice Research Datalink (CPRD) GOLD UK primary care database.

Participants:

Female patients with at least one pregnancy episode in the Register which had no recorded outcome or conflicted with another episode.

Design:

We identified multiple scenarios potentially explaining why uncertain episodes occur. Criteria were established and systematically applied to determine whether episodes had evidence of each scenario. Linked Hospital Episode Statistics were used to identify pregnancy events not captured in primary care

Results:

Of 5.8 million pregnancy episodes in the Register, 932,604 (16%) had no recorded outcome, and 478,341 (8.5%) conflicted with another episode (251,026 distinct conflicting pairs of episodes among 210,593 women). 826,146 (89%) of the episodes without outcome recorded in primary care and 215,577 (86%) of the conflicting pairs were consistent with one or more of our proposed scenarios. For 689,737 (74%) episodes with recorded outcome missing and 215,544 (86%) of the conflicting pairs (at least one episode) supportive evidence (e.g. antenatal appointment records, linked hospital records) suggested they were true and current pregnancies. Furthermore, 516,818 (55 %) and 160,936 (64%) respectively were during research quality follow-up time. For a sizeable proportion of

uncertain episode there is evidence to suggest that historical outcomes being recorded by the GP during an ongoing pregnancy may offer explanation (73,208 (29.2%) and 349,874 (37.5%)).

Conclusions:

This work provides insight to users of the CPRD Pregnancy Register on why uncertain pregnancy episodes exist and indicates that most of these episodes are likely to be real pregnancies. Guidance is given to help researchers consider whether to include/exclude uncertain pregnancies from their studies, and how-to tailor approaches to minimise underestimation and bias.

Strengths and Limitations of this study

- This work enables researchers to make informed decisions about whether to include incomplete and uncertain pregnancy data when designing studies in electronic health records.
- Detailed scenarios are described as to why uncertain pregnancy episodes may occur along with criteria which researchers can apply to ascertain which episodes may fit each scenario.
- Clinician advice and clinical guidelines were used to as to generate assumptions as to why and when clinicians may record information relating to pregnancy however, these may not be correct in every case.
- Electronic health data is not collected for the purposes of research and can be messy for a variety of reasons, some of which may not have been captured in this study.

Introduction

Understanding how diseases, drugs and other exposures affect pregnant women and their children is an important public health priority. However, pregnant women are excluded from many trials due to potential risks to the woman and her unborn child. Observational research using electronic healthcare records (EHR) has thus become a well-established vital tool for investigating disease prevalence, risk factors and pharmacovigilance in pregnant women. UK primary care databases are particularly useful due to the gate-keeper healthcare system meaning all antenatal care is overseen by a general practitioner (GP) (1). One example of such a database is CPRD GOLD. This database is produced and maintained by the Clinical Practice Research Datalink (CPRD), a government research service collecting de-identified and fully coded patient-level EHR from primary care practices across the UK (2). However, challenges such as incomplete data capture in EHR data can make it difficult to identify accurately the start and end of pregnancies. Recently a collaboration between CPRD and the London School of Hygiene and Tropical Medicine (LSHTM) established a Pregnancy Register of all pregnancies in CPRD GOLD (3) which includes approximately six million estimated pregnancies (henceforth, pregnancies in the Register will be referred to as pregnancy episodes).

Previous approaches to generating pregnancy registers have been limited by the exclusion of pregnancies without identified outcomes and pregnancy records which do not fit chronologically into an identified pregnancy episode (4). Ignoring these records potentially excludes periods when women were pregnant. If these pregnancies systematically differ from those captured more completely, their exclusion may lead to bias. For example, pregnancies ending in miscarriage may be less likely to have the outcome recorded than pregnancies ending in live birth (3). Ignoring pregnancy data which is challenging to interpret may therefore underestimate adverse outcomes. Incomplete capture of pregnancies also impacts descriptive studies that need pregnancies as

For peer review only - http://bmjopen.bmj.com/site/about/guidelines.xhtml

denominator data, such as vaccine uptake studies. A further limitation of previous approaches is that some women have pregnancies that seemingly overlap in the data, and these are not addressed. These conflicting pregnancies highlight that estimated timings of some pregnancies may be suboptimal and/or some pregnancy episodes may not be true pregnancies. Approaches which exclude incongruent or incomplete pregnancy data may lead to misclassification of exposure timings.

The unique advantage of the CPRD Pregnancy Register is that it utilises all pregnancy data in CPRD GOLD, thereby capturing all documented pregnancies regardless of completeness. However, this also presents interpretational challenges: approximately 950,000 pregnancy episodes (16% of all pregnancy episodes) have no outcome recorded and approximately 500,000 pregnancy episodes conflict with another episode for the same woman (episodes identified by the algorithm with at least one day of overlap). These episodes are flagged in the Register enabling researchers to identify them when designing their study. However, there may be multiple reasons for the occurrence of uncertain episodes and therefore absolute rules on whether to include or exclude them from a study may be inappropriate.

We therefore aimed to investigate possible reasons why the algorithm used to generate the CPRD Pregnancy Register identifies uncertain episodes and thus generate information to guide future use of this important resource. Our specific objectives were:

1. To identify potential scenarios which may result in pregnancy episodes without a recorded outcome or those which conflict with another episode for the same woman.
2. To use available data (including linked data) to investigate these potential scenarios and flag pregnancy episodes which are consistent with each one.
3. To provide information to researchers using the Register to help inform their decisions on how to handle these uncertain episodes when designing studies.

Methods

Data Sources

CPRD Primary care data and the Pregnancy Register

The CPRD GOLD UK primary care database contains registration information and all care events that general practice staff record to support clinical care. This includes demographic information (birthyear, sex, etc.), clinical events (signs, symptoms, medical diagnoses), referrals to specialists and secondary care, prescriptions issued in primary care, vaccinations, test results, lifestyle information (e.g. smoking status), and other care administered as part of GP practice (5). CPRD data also contain indicators of data quality at the patient level (known as the acceptability flag Appendix 1) and at the practice level (known as the practice up to standard date Appendix 1). As CPRD GOLD is a longitudinal database, updated monthly, it contains variables indicating whether the patient and practice are still contributing data.

The Pregnancy Register lists and characterises all pregnancies identified in CPRD GOLD based on an algorithm (3). A single record represents a unique pregnancy episode. Each woman may have

For peer review only - http://bmjopen.bmj.com/site/about/guidelines.xhtml

multiple episodes. Information includes the estimated start and end of pregnancy, its outcome (when recorded) and whether it was a singleton or multiple pregnancy. For live birth pregnancies, patient identifiers of linked babies identified through the CPRD Mother-Baby-Link (MBL) (6) are provided. Figure 1 gives an overview of the algorithm steps and Appendix 2 gives a list of the variables provided in the Register. Figure in Appendix 3 shows an example of how a real pregnancy might manifest in (a) raw CPRD gold data, and (b) the processed pregnancy register dataset.

Linked data

Person-level linkage of CPRD primary care data with other datasets (e.g. Hospital Episode Statistics HES) is available for English practices who have consented to participate in the linkage scheme (7). These linkages cover approximately ~56% of contributing CPRD GOLD practices in the UK. Where available we utilised linked data to look for further information about the pregnancy episodes within the Register. HES APC (Admitted Patient Care) data includes information on admission and discharge dates, diagnoses, specialists seen and procedures undertaken for linked patients with a hospitalisation record (8) We searched HES APC data for records of pregnancy outcomes using ICD 10 and OPCS codes (Appendix 4 and 5). HES APC Maternity records were also utilised: a recording of an acceptable value in any of the variables identified as relating to delivery (Appendix 6) was taken as evidence that a delivery had taken place.

The HES Diagnostic Imaging Dataset (DID) provides detailed information about diagnostic imaging tests, including x-rays, MRI scans and foetal growth scans, taken from NHS providers' radiological information systems. This was used for records of foetal scans. Office for National Statistics (ONS) mortality data was also used to ascertain additional death records which may have been missing from CPRD.

Study Population

This study included all individuals who had a least one pregnancy episode without a recorded outcome or at least one conflicting pregnancy episode in the February 2018 version of the Pregnancy Register. All pregnancy records for these patients were extracted from the CPRD GOLD database using the pregnancy code-list upon which the pregnancy algorithm is based (3) thereby creating a dataset which included all pregnancy records and the summary Pregnancy Register information for these women. Women were followed-up until the minimum of leaving the practice, death or practice last collection date. In the linked data analysis women with HES records beyond this point were followed-up until the end of linked data coverage.

Identifying scenarios to explain the occurrence of uncertain episodes

Potential scenarios which may result in uncertain pregnancy episodes, including those without recorded outcomes and those which conflicted with another episode, were identified through discussions with the creators of the Register (CM, ST, RW), clinicians and CPRD data experts. The scenarios are based on the structure of the CPRD GOLD data and the Pregnancy Register algorithm (Figure 1, steps 1-8)). The scenarios are not mutually exclusive; thus, episodes may be consistent with more than one scenario.

Pregnancy Episodes with no recorded outcome

Scenarios with the potential to result in episodes with missing outcomes were identified. There are four overarching problems with various specific scenarios within them: the pregnancies are true and current , but the outcome was not captured in CPRD primary care data; the pregnancies are true and

current , but the pregnancy was still ongoing at the end of follow up in the database; the patient was not pregnant at the time of the database record; the pregnancy is really part of another pregnancy episode in the Register. The twelve scenarios which fall under these problems are described in Table 1a.

Conflicting Pregnancy Episodes

Scenarios with the potential to result in conflicting episodes were proposed and are described in detail in Table 1b. Identifying the scenarios was an iterative process, after applying initial scenarios we took a sample of 50 conflicting pregnancy episodes and reviewed the patient data. This allowed us to validate existing scenarios and identify further scenarios. Scenarios can be grouped under four overarching problems: both pregnancies are true but one is a historical pregnancy; both pregnancies are historical; both pregnancies are true and current but the gestation of the second pregnancy estimated by the algorithm is too long; the woman was pregnant, but one pregnancy has been split into multiple episodes by the rules of the algorithm (Appendix 3).

For peer review only

Table 1a: Description of potential scenarios leading to pregnancy episodes with no recorded outcome and scenario criteria applied

Scenario	How does this appear in the data?	Criteria used to determine if there is evidence in the data that an episode is consistent with the scenario in question
Problem 1: The women was pregnant at the time of the database record, but the outcome ***was not captured in CPRD primary care data.***		
1a. The woman was pregnant. She had a delivery, miscarriage, or termination of pregnancy (TOP) in hospital or elsewhere and information either was not fed back to the general practice, or was fed back but not coded in the woman's records	There will be no evidence of an outcome in CPRD data up to 38 weeks* (for delivery) or up to 20 weeks (for miscarriage or TOP) after the first antenatal record for the pregnancy. However, there may be evidence of delivery/miscarriage/TOP in one of the linked HES APC data.	• The woman must be eligible for linkage.<br>• There must be at least one day of overlap between the data coverage for each HES source and the pregstart + 294 days (42 weeks) to give a maximum potential end date.<br>• There must be a record in HES of delivery or loss within 294 days (42 weeks)
1b. The pregnancy outcome was recorded in the primary care data but has no event date recorded alongside it and is therefore not picked up by the algorithm.	There will be an outcome code with missing eventdate** within 38 weeks after the first antenatal record of the pregnancy episode (using the systemdate**as a proxy for the event date)	• There must be an antenatal code with missing eventdate** recorded with a systemdate** >= 294 days after pregnancy episode start

For peer review only - http://bmjopen.bmj.com/site/about/guidelines.xhtml

1c. The pregnancy outcome occurred before the patient was registered at their current practice or before the start of the practice up-to-standard follow-up (UTS). When the patient joined the practice, information was recorded about the pregnancy but not the outcome.	The pregnancy episode will occur before the start of the patient's current registration and/or UTS.	• Pregnancy episode end date must be < up to standard (uts) date** OR <= current registration date
Problem 2: The women was pregnant at the time of the database record***, but the pregnancy was still ongoing at the end of available follow up in the database.***		
2a. The woman moved practices before the end of her pregnancy. If a patient transfers out of a CPRD practice, then follow up is lost. OR The woman died before the end of her pregnancy.	There will be a transfer out date or death date (in either CPRD or the ONS mortality data) less than 38 weeks after the earliest antenatal record for the pregnancy episode.	• The earliest of the woman's transfer out date** or death date (in either CPRD or the ONS mortality data) minus pregnancy episode start date must be < = 294 days
2b. The last collection of data from the practice was before the pregnancy outcome.	There will be a last collection date less than 42 weeks after the start of the pregnancy episode.	• The woman's last collection date minus pregnancy episode start date must be < = 294 days
Problem 3: The patient was not pregnant at the time of the database record.		

For peer review only

3a. A historical pregnancy was recorded retrospectively in the first few months after patient joins the practice. In this scenario information about the pregnancy is recorded with the current date rather than the date it occurred (different from scenario 1c). This is more likely to occur when a woman joins a practice and the GP may wish to record past pregnancy events which are relevant to her current clinical care	The pregnancy episode will occur less than one year after the women's current registration date. There will be a record of a pregnancy event which may be clinically useful for future care between the start and end of the pregnancy episode.	• Pregnancy episode start date is < 365 days after current registration date.<br>• There is a record of a pregnancy code from a list identified as likely to be recorded as useful pregnancy history information (Appendix 7).<br>• This must have an eventdate >= pregstart ** & <=pregend**
3b. The woman was not pregnant but was planning a pregnancy and discussed this with the GP, e.g. due to other medical conditions which may complicate pregnancy.	The pregnancy episode will include a pregnancy advice code, for example "67AF.00 Pregnancy advice for patients with epilepsy"	• The woman has antenatal codes identified as pregnancy advice codes (Appendix 8) with an eventdate** >= pregstart** & <= pregend **
Problem 4: The pregnancy record belongs to another pregnancy episode in the Register.		

For peer review only - http://bmjopen.bmj.com/site/about/guidelines.xhtml

4a. There was a delay in recording the outcome of a pregnancy by the practice. Thus, the outcome code has an eventdate** which is later than the true outcome date. The algorithm then calculates the LMP as being later than it was (Figure 1, steps 5 and 6). Records which occurred early in pregnancy are then left unassigned to the pregnancy episode and appear as if belonging to a previous pregnancy episode which has no outcome recorded (Figure 1, step 8).	As the pregnancy episode without outcome has been created from unassigned records at the beginning of the pregnancy it will be followed by another pregnancy episode. There is unlikely to be more than a three-month delay in outcome recording due to the mother attending the practice for postnatal checks and/or infant vaccinations. Therefore, there will be less than 12 weeks between the end of the episode with no recorded outcome and the start of the next pregnancy episode.	• The woman must have >1 episode in the pregnancy register.<br>• Episodes with recorded outcome missing were eligible if they were not the last pregnancy episode for that woman.<br>• There must be <= 84 days (12 weeks) between the pregend** of the episode without outcome and the pregstart** of the woman's next episode.

For peer review only

4b. The LMP is derived from information in the data and is estimated by the algorithm to have occurred later than reality (Figure 1, steps 5). This may lead to a short pregnancy episode and unassigned codes before the estimated start of pregnancy. These are then grouped to form a pregnancy episode with no recorded outcome (Figure 1, step 8).	The pregnancy episode without outcome will be followed by another pregnancy episode which will be less than 40 weeks long.	• The woman must have >1 episode in the pregnancy register.<br>• The episode after the episode with missing outcome must have a startsource** = to 2,4,5 or 6 (Appendix 2). The length (gestdays) of the episode must be <280 days
4c. If there are pregnancy records within 4 weeks before the estimated LMP, the identified pregnancy episode is shifted earlier in time by the algorithm (within plausible limits) to encompass those records (Figure 1, step 6). This may leave unassigned pregnancy records which occurred shortly after the new estimated delivery date which will then be grouped to form a pregnancy episode with no recorded outcome (Figure 1, step 8).	The pregnancy episode must not be the only pregnancy for this to apply. There will be another pregnancy episode which ends <8 weeks before the first antenatal record of the pregnancy episode without outcome for which the end has been adjusted by the algorithm.	• The woman must have >1 episode in the pregnancy register.<br>• The episode before the one with recorded outcome missing must have an endadj** =2 (Appendix 2)<br>• The pregend** date for the episode with missing outcome must be <= 56 days (8 weeks) after the pregend** for that previous episode.

For peer review only

4d. The GP records a code relating to the patient's pregnancy outcome history whilst the patient is pregnant. This is incorrectly identified by the algorithm as the outcome of the current pregnancy (Figure 1, step 3). If the actual outcome is <=25 weeks after for delivery or <=12 weeks after for pregnancy losses they will be grouped together as the same outcome. Subsequent antenatal records may then be grouped together to form a new pregnancy episode with no recorded outcome (Figure 1, step 8)	The pregnancy episode must not be the patient's first pregnancy. The pregnancy episode would be within 25 weeks after the previous outcome.	• The woman must have >1 episode in the pregnancy register.<br>• The pregend** date for the episode with missing outcome had to be <= 175 days (25 weeks) after the pregend** for the previous episode.
4e. The outcome of the pregnancy episode has been misclassified as an antenatal event e.g. 'Failed abortion', 'refer to TOP counselling', 'premature labour' etc.	There will be an antenatal code which should have been an outcome code within 38 weeks after the first antenatal record of the pregnancy episode with recorded outcome missing.	• There must be an antenatal record from a code list of potentially misclassified outcomes (Appendix 9) 266 days (38 weeks) of the firstantenatal** record.

*The first antenatal record is assumed to be recorded ≥4 weeks after the LMP as the woman is unlikely to know she is pregnant before then.

** Refers to a CPRD GOLD specific variable for example: pregend = the end of episode as defined by the algorithm; pregstart = the start of episode as defined by the algorithm; endadj = an indication that the end of the episode has been adjusted and how; startsource= which data were used to generate the start of the episode. These variables and others are defined in more detail in appendix 2

Table 1b: Description of potential scenarios leading to conflicting episodes and scenario criteria applied

Scenario	How does this appear in the data?	Criteria applied to pairs of conflicting episodes to determine if there is evidence in the data that the pair is consistent with the scenario in question.
Problem 1: Both pregnancies are true, but one is a current pregnancy and one is a historical pregnancy		
1a. The GP records a past delivery during a current pregnancy > 25weeks before the true delivery of that pregnancy. OR a past pregnancy loss > 12 weeks before the actual loss of that pregnancy	Both pregnancies will have the same outcome type. Evidence of current pregnancy codes would be expected to fall within the second pregnancy.	• The outcome combination of the two episodes must be delivery/delivery or loss/loss (see Appendix 10 for outcome classifications)<br>• The second episode had an antenatal code from a list deemed likely to only be recorded if the patient was currently pregnant (Appendix 11) OR a scan record in the HES DID data between firstantenatal* and pregend*.

For peer review only

1b. If a patient has a record relating to a previous loss recorded during a pregnancy ending in delivery or vice-versa then conflicting episodes will be created by the algorithm. The algorithm first generates episodes for consecutive deliveries; it then does the same thing for pregnancy losses. There is no step in the algorithm to check that the loss episodes do not coincide with the delivery episodes (Figure 1, steps 1-6).	The conflicting pregnancies must consist of one loss and one delivery.<br><br>Evidence of current pregnancy codes would be expected to fall within the second pregnancy.	• The outcome combination of the two episodes must be delivery/loss or loss/delivery (see Appendix 10 for outcome classifications)<br><br>• The second episode had an antenatal code from a list deemed likely to only be recorded if the patient was currently pregnant (Appendix 11) OR an antenatal scan record in the HES DID data between firstantenatal* and pregend*
Problem 2: Both pregnancies are historical		
2a. A patient joins a new practice (or has another reason for a full obstetric history to be taken) and has information on historical pregnancies recorded with the current date rather than the actual date of the event. Losses and deliveries recorded on the same date will result in conflicting episodes in the Register as different outcome types are generated separately by the algorithm (Figure 1, steps 1-5).	The conflicting pregnancies must consist of one loss and one delivery. The pregnancy end dates will be the same for both pregnancies. Both pregnancies are likely to be <1 year after the patient's current registration date. We would not expect to find codes indicating current pregnancy.	• The outcome combination of the two episodes must be a delivery and a loss.<br><br>• The pregend* dates must be the same.<br><br>• There must be no antenatal codes relating to current pregnancy (Appendix 11) or HES DID antenatal scan recorded between the firstantenatal* date and the pregend* date of either episode.

Problem 3: Both pregnancies are true and current but the gestation of the second pregnancy estimated by the algorithm is too long.		
3a. The woman has two pregnancy losses which are >8 weeks and <12 weeks apart. The second pregnancy has no information about gestation recorded so the algorithm applies a default of 12 weeks and the episodes overlap.	Both conflicting pregnancies must be losses. The maximum overlap between the two pregnancies must be 4 weeks. Evidence of current pregnancy codes could be found in either pregnancy.	• The outcome combination of the two episodes must be two losses. The pregend* for the first episode must be <= 28 days after the pregstart* of the second episode.
3b. The woman has two pregnancies close together and the second pregnancy ends in delivery. If the information on the LMP in the data of the second pregnancy is wrong, then the algorithm may generate the start too early resulting in an overlap.	The second pregnancy must be a delivery and have no information about gestation in the data. The overlap must be <15 weeks (otherwise the two outcomes would be <25 weeks apart and would have been grouped as one see Figure 1 step 3). There may be evidence of current pregnancy codes in either pregnancy	• The outcome of the second episode must be a delivery.<br>• The startsource* of the second episode must be not equal to 4 or 5 (Appendix 2)<br>• The pregstart *of the second episode must be <105 days (15 weeks) before the pregend* of the first episode.
Problem 4: The pregnancy is true and current but is split into separate episodes by the rules of the algorithm		

For peer review only

4a. The GP records further information about a pregnancy outcome > 25 weeks after the delivery date for pregnancies ending in delivery OR >8 weeks but <12 weeks for pregnancies ending in loss. The algorithm assumes this further information is a different pregnancy and generates a new episode, which may overlap with the "true" episode.	Both pregnancies must be of the same outcome type. Evidence of current pregnancy codes would be expected to fall within the first pregnancy.	• The outcome combination of the two episodes must be delivery/delivery or loss/loss (Appendix 12)<br>• The first episode had an antenatal code from a list deemed likely to only be recorded if the patient was currently pregnant (Appendix 11) OR a scan record in the HES DID data between firstantenatal* and pregend*.
4b. The GP records further antenatal information about a pregnancy after delivery or pregnancy loss. This will then be used to generate a new pregnancy without outcome episode by the algorithm. If the code is within 4 weeks of the end of the true pregnancy episode the two will overlap	The first pregnancy must be a pregnancy with an outcome recorded in the data. The second pregnancy must be a pregnancy without outcome which consists of one antenatal code not related to a scan.	• The first episode must have outcome= 1-10 in the register (Appendix 2) and must have endadj* =0<br>• The second episode must have no recorded outcome (outcome= 13)<br>• The second episode must have a gestdays* =28 (likely to consist of one code) and there must NOT be a scan code (Appendix 13) with an eventdate* = pregend* of the second episode.

For peer review only

4c. The patient has a follow up scan after a pregnancy loss. This is recorded in the data by the GP as an antenatal scan. The algorithm then creates a second pregnancy episode based on the antenatal scan code which becomes a pregnancy without outcome in the register.	The first pregnancy must be a pregnancy loss. The second pregnancy must be a pregnancy without outcome which consists of one antenatal code related to a scan.	• The outcome combination of the two episodes must be loss/missing.<br>• The second episode must have a gestdays* =28 (likely to consist of one code) and there must be a scan code (Appendix 13) with an eventdate* = pregend* of the second episode.

For peer review only

4d. The GP records information about a pregnancy but no information about the outcome. If records relating to this pregnancy are more than 6 weeks apart, they will be turned into multiple episodes. Once estimated start dates are generated for these episodes based on the data recorded (Figure 1, step 8) episodes may overlap. For example, if there is gestational information included in the second episode the start of this episode will be assigned before the start of the previous episode resulting in a nested pregnancy episode.	Both pregnancies must be pregnancies without outcome in the register. The end of the first pregnancy must be greater than six weeks before the first antenatal of the second.	• The outcome combination of the two episodes must be missing/missing.<br>• The pregend* of the first episode is > 42 days before the firstantenatal* date of the second episode.

For peer review

4e. The first pregnancy episode ended in delivery and has been shifted backwards by the rules of the algorithm leaving unassigned late pregnancy or third trimester records. These records will then be identified by the algorithm as end of pregnancies (Figure 1, step 6) and new conflicting episodes will be created.	The first pregnancy must be a pregnancy with a delivery outcome recorded in the data. The end of the first pregnancy must have been adjusted. The second pregnancy must be a pregnancy where the outcome is based on a late pregnancy or third trimester record.	• The first episode must have a delivery outcome code and endadj* variable not = to 0<br>• The second episode must have outcome= to 11, 12 or 13.

* Refers to a CPRD GOLD specific variable for example: pregend = the end of episode as defined by the algorithm; pregstart = the start of episode as defined by the algorithm; endadj = an indication that the end of the episode has been adjusted and how; startsource= which data were used to generate the start of the episode. These variables and others are defined in more detail in appendix 2

For peer review only

Applying Criteria to identify evidence of each scenario.

Evidence in HES

For each episode it was ascertained whether the woman was eligible for linkage to other data and whether the episode occurred within the coverage period of each linked data source. For pregnancy episodes occurring within the linkage coverage period, the linked HES data was examined for evidence of pregnancy outcomes. The period for which outcomes were searched was from the episode start date to nine months after the episode end date; we excluded from this analysis pregnancies where this period was entirely outside the coverage dates for linked HES data.

ICD 10 and OPCS code lists were used to look for evidence of outcomes in the HES APC Episodes, Diagnosis and Procedures tables (Appendix 4 and 5). In the HES APC Maternity data a recording of an acceptable value in any of the variables identified as relating to delivery (Appendix 6) was flagged as evidence that a delivery had taken place. In the HES OP data an ICD 10 code list for evidence of delivery, termination or early pregnancy loss was used. Snomed codes (Appendix 14) were used to identify all foetal scan records in the HES DID data.

Pregnancy Episodes with recorded outcome missing

All episodes coded as outcome unknown ("13" in the outcome field) were extracted from the Pregnancy Register. For each episode, we extracted information on the timing of the episode in relation to the start and end of patient follow-up and the period of research standard ("up to standard", or UTS) data recording in CPRD, and we also searched for relevant codes in the patient's record, namely: early pregnancy codes which were likely to be recorded in the patient's first antenatal visits to the GP; codes which are likely to be recorded by the GP as clinically important in the patient's medical history even when the patient was not pregnant; codes which may indicate an outcome but were originally classified by the Register as antenatal; codes which are likely to be recorded by the GP as part of a consultation about the potential health impacts on a patient of becoming pregnant (code lists in Appendix 7, 9, 8).

For each scenario a set of criteria based on how these should appear in the data were established (described in detail in Table 1a). Criteria were systematically applied to the data to establish which episodes were consistent with each scenario.

Conflicting pregnancy episodes

All conflicting episodes (those with at least one day of overlap with another episode for the same woman) were ascertained using the conflict flag in the Register. Pregnancy episodes may conflict with more than one other episode. Each conflicting pair was treated separately and therefore an individual pregnancy episode could appear in the analysis multiple times. A dataset was created which contained one row per pair of conflicting pregnancy episodes.

For peer review only

Episodes were ordered by start date with episode one being the earlier start date of the two. Descriptive variables were added to the dataset from the CPRD GOLD data to indicate if the episodes were during current registration and UTS follow-up. Pregnancy episode outcomes were grouped into three categories: delivery, loss or missing, and a variable was generated to indicate the combination of outcomes in each conflicting pair (Appendix 12).

For each scenario a set of criteria based on how these should appear in the data were established (described in detail in Table 1b). Criteria were systematically applied to the data to establish which conflicting pairs were consistent with each scenario.

Patient and Public Involvement

There was no patient or public involvement in this methodological work.

Results

There were 2,438,493 women with a pregnancy episode in the February 2018 version of the Pregnancy Register of these patients 731,368 (30%) had at least one uncertain episode. Mean patient follow up time for all women was 4,720 days, this was slightly lower for women with a missing outcome record (4,349 days) (Table 2). Women with an uncertain episode were more likely to be over 30 years of age. Uncertain pregnancy episodes were also more likely to be recent (after 2000) (Table 2).

Pregnancy Episodes with recorded outcome missing

Of the 5.8 million pregnancy episodes in the Pregnancy Register there were 932,604 (16%) episodes with no recorded outcome of which over half (516,818, 55.4%) were during UTS follow up and current registration (Table 2). 826,146 (89%) had evidence consistent with at least one of the identified scenarios (Table 3). 689,737 (74%) had evidence of a scenario indicating they were true (either current or historical) pregnancies (scenarios 1a,1b, 1c, 2a, 2b or 4e). The largest proportion of pregnancy episodes occurred before the patient registered at their current practice which contributed the data to CPRD or before that practice was deemed to be contributing research standard data (415,807, 44.6% Scenario 1c). 211,070 (22.6%%) episodes had data in HES consistent with the outcome occurring in hospital and not being fed back to the GP (Scenario 1a), representing approximately 50% of episodes with recorded outcome missing which were eligible for linkage. HES APC data was the most useful linked data source for ascertaining pregnancy outcomes with a small number found in HES Outpatient (Appendix 15).

The second most common potential explanation for pregnancies without outcome was scenario 4d, where a code relating to the patient's pregnancy history may have been recorded by the GP whilst the patient was pregnant. 349,874 (37.5%) episodes without outcome were consistent with this scenario. Relatively fewer episodes were consistent with scenario 4a, 4b and 4e, none were consistent with 4c. For 242,698 (26%) episodes, follow-up ended before the predicted end of the pregnancy (Scenario 2a and 2b) for 822 episodes (<0.1%) of these episodes follow-up ended due to death. Only small proportions of episodes were consistent with other scenarios.

For peer review only

Table 2: Baseline characteristics of the pregnancy episodes in the February 2018 Pregnancy Register.

	**Episodes with recorded outcome missing N (%)**	**Conflicting episodes N (%)**	**All episodes in the Pregnancy Register N (%)**
Number of patients	*643,689 (26.4%)*	*210,593 (8.6%)*	*2,438,493*
Mean patient follow up time (years)	*11.92*	*12.92*	*12.93*
Mean number of pregnancy episodes per patient	*3.63*	*4.66*	*3.44*
Pregnancy end was during UTS follow up and current registration	516,818 (55.4%)	160,936 (64.1%)	1,926,077 (33.1%)
Age Group of the patient at the end of the pregnancy episode			
11-14	1,344 (0.1%)	76 (0.0%)	7,867 (0.1%)
15-19	72,543 (7.8%)	15,420 (6.1%)	551,025 (9.5%)
20-24	196,979 (21.1%)	48,273 (19.2%)	1397717 (24.0%)
25-29	254,352 (27.3%)	65,601 (26.1%)	1624350 (27.9%)
30-34	235,995 (25.3%)	69,236 (27.6%)	1339439 (23.0%)
35-39	126,369 (13.6%)	40,079 (16.0%)	685,421 (11.8%)
40-44	37,640 (4.0%)	11,355 (4.5%)	194,354 (3.3%)
45-49	7,382 (0.8%)	953 (0.4%)	24,208 (0.4%)
Year pregnancy episode ended			
pre 1950	1,417 (0.2%)	41 (0.0%)	16,695 (0.3%)
1950-1959	8,061 (0.9%)	522 (0.2%)	98,436 (1.7%)
1960-1969	19,312 (2.1%)	1,887 (0.8%)	283,757 (4.9%)
1970-1979	24,296 (2.6%)	3,882 (1.5%)	493,217 (8.5%)
1980-1989	38,768 (4.2%)	9,135 (3.6%)	803,380 (13.8%)
1990-1999	248,016 (26.6%)	54,254 (21.6%)	1,530,212 (26.3%)
2000-2009	336,523 (36.1%)	116,429 (46.4%)	1,705,380 (29.3%)
2010-2018	256,211 (27.5%)	64,843 (25.8%)	893,304 (15.3%)
**Total Pregnancies**	**932,604**	**251,026**	**5,824,381**

For peer review only

Table 3: Numbers of pregnancy episodes with recorded outcome missing which were consistent with applied criteria for each scenario**

**Scenario**	**Description**	**N pregnancy episodes with evidence of this scenario (% of total episodes with missing outcome)**	**N pregnancy episodes with evidence of this scenario only (% of total episodes with missing outcome)**	**N pregnancy episodes with evidence of an outcome in linked HES (% of linkage eligible episodes with recorded outcome missing*)**	**N episodes which were during current registration and UTS follow up (% of total episodes with missing outcome)****
Denominator		932,604	932,604	424,375*	932,604
Problem 1: The women was pregnant at the time of the database record, but the outcome was not captured in CPRD primary care data.					
Scenario 1a	The pregnancy outcome occurred in hospital or elsewhere and information wasn't fed back to the practice.	211,070 (22.6%)	1,934 (0.2%)	211,070 (49.7%)	139,084 (14.9%)
Scenario 1b	The outcome of the pregnancy is recorded in the primary care data but has no event date associated with it.	1,595 (0.2%)	48 (0.0%)	523 (0.1%)	475 (0.1%)
Scenario 1c	The pregnancy occurred before the patient was registered at the practice or before UTS	415,807 (44.6%)	204,176 (21.9%)	60,423 (14.2%)	0 (0.0%)

For peer review only - http://bmjopen.bmj.com/site/about/guidelines.xhtml

Problem 2: The women was pregnant at the time of the database record, but the pregnancy was still ongoing at the end of available follow up in the database.					
Scenario 2a	The patient transferred out or died before the putative end of pregnancy	177,557 (19.0%)	40,191 (4.3%)	71,012 (16.7%)	117,571 (12.6%)
Scenario 2b	The last collection date of the practice was before the putative end of pregnancy	65,141 (7.0%)	22,039 (2.4%)	24,091 (5.7%)	58,698 (6.3%)
Problem 3: The patient was not pregnant at the time of the database record.					
Scenario 3a	Episode is derived from historical pregnancy information recorded in the first few months after the patient joined the practice	10,235 (1.1%)	588 (0.1%)	3,058 (0.7%)	3,875 (0.4%)
Scenario 3b	Patient asks for advice whilst planning a pregnancy	0 (0.0%)	0 (0.0%)	0 (0.0%)	0 (0.0%)
Problem 4: The pregnancy record belongs to another pregnancy episode in the Register.					
Scenario 4a	Delay in recording the outcome of a pregnancy, algorithm calculates LMP too late and uncovers records at the beginning of pregnancy creating this PWO.	61,662 (6.6%)	9,299 (1.0%)	23,099 (5.4%)	35,255 (3.8%)

For peer review only - http://bmjopen.bmj.com/site/about/guidelines.xhtml

Scenario 4b	The LMP is derived from the data and is wrong resulting in early codes being uncovered creating this episode	29,057 (3.1%)	4,022 (0.4%)	11,304 (2.7%)	17,110 (1.8%)
Scenario 4c	The LMP has been shifted earlier in time uncovering records at the end of the pregnancy	0 (0.0%)	0 (0.0%)	0 (0.0%)	0 (0.0%)
Scenario 4d	A code recorded relating to the patient's delivery history is incorrectly identified by the algorithm as a delivery uncovering records at the end.	349,874 (37.5%)	113,688 (12.2%)	90,274 (21.3%)	219,505 (23.5%)
Scenario 4e	The outcome of the pregnancy episode has been misclassified as antenatal	38,848 (4.2%)	8,000 (0.9%)	6,611 (1.6%)	18,222 (2.0%)
None	These pregnancy episodes did not meet the criteria for any identified scenarios.	106,458 (11.4%)	-	-	94,769 (10.2%)

* Denominator = pregnancy episodes which had at least one day overlap with the available HES follow up period and where the woman was eligible for linkage.

**A version of this table restricted to episodes which occurred during practice UTS follow-up and patient's current registration is given in the appendices (Appendix 16)

For peer review only

Conflicting Pregnancy Episodes

There were 478,341 (8.5%) pregnancy episodes with a conflict recorded in the February 2018 Pregnancy Register, amounting to 251,026 conflicting pregnancy pairs. Over half of the pairs (160,936, 64%) were during UTS follow up and current registration. There were 215,577 (88.6%) pairs which were consistent with at least one identified scenario. Of the remaining 106,458 (11.4%), less than half were during UTS follow up and current registration (table showing these pregnancies by scenario is given in Appendix 17). Across all scenarios at least 40% were during UTS follow up and current registration. Of the pregnancy pairs 215,544 (86%) had evidence of a scenario indicating that at least one episode was a true and current pregnancy (scenarios 1a, 1b, 3a, 3b, and 4a-e). Most conflicting pairs had at least one pregnancy episode ending in loss 201,783 (80.3%) (Appendix 18). Furthermore, 41% (101,760) of pairs included at least one pregnancy with no outcome recorded.

75,672 (30%) of all conflicting pairs were shown to have evidence that they were consistent with problem 1, that a patient had a record relating to the outcome of a previous pregnancy recorded during a current pregnancy. This includes scenario 1b: a record of a previous loss recorded during a pregnancy ending in delivery or vice versa, one of the most common scenarios (29% of conflicting pairs) (Table 4).

73,191 (29%) of pairs were consistent with scenario 4e: that adjusting of pregnancy dates by the algorithm had led to unassigned records. Of these, over 96% (70,472) were consistent with this scenario only, and 73% (53,464) of these pairs had a linked baby identified. 43,581 (17.4%) of episodes had evidence that they were consistent with further antenatal information having been recorded after the end of pregnancy (scenario 4b).

For approximately 16% (39,373) of conflicting pairs there was evidence to suggest that the gestation of the second pregnancy episode specified by the algorithm may have been too long leading to an overlap (scenario 3a and 3b).

Ten percent of conflicting pairs had a loss and delivery recorded on the same date and no "current pregnancy" antenatal codes suggesting they may have been recorded as part of an obstetric history (scenario 2a). Only small percentages of episodes were consistent with other scenarios.

For peer review only

Table 4: Numbers of conflicting pregnancy episodes which were consistent with applied criteria for each scenario**

Scenario	Description	N pregnancy pairs with evidence of this scenario (% of total conflicting pregnancy pairs)	N pairs with evidence of only this scenario (% of total conflicting pregnancy pairs)	N of pairs with a linked baby in the MBL (% of total conflicting pregnancy pairs)	N pairs with evidence of pregnancy in linked HES (% of pairs eligible for HES linkage*)	N pairs which were during current registration and UTS follow up MBL (% of total conflicting pregnancy pairs)
Denominator		251,026	251,026	251,026	160,461*	251,026
Problem 1: Both pregnancies are true but one is a current pregnancy and one is a historical pregnancy						
Scenario 1a	The GP records a past delivery or loss during a current pregnancy with the same outcome resulting in another episode being created	2,464 (1.0%)	413 (0.2%)	2,164 (0.9%)	2,332 (1.5%)	1,981 (0.8%)
Scenario 1b	A patient has a record relating to a loss recorded during a pregnancy ending in delivery or vice-versa. Conflicting episodes are generated by the algorithm	73,208 (29.2%)	35,026 (14.0%)	11,388 (4.5%)	19,900 (12.4%)	31,526 (12.6%)
Problem 2: Both pregnancies are historical						
Scenario 2a	A patient has information on historical pregnancies recorded with the current date rather than the actual date.	27,250 (10.9%)	0 (0.0%)	175 (0.1%)	6,835 (4.3%)	12,557 (5.0%)
Problem 3: Both pregnancies are true and current but the gestation of the second pregnancy estimated by the algorithm is too long.						
Scenario 3a	The woman has two losses which are >8weeks and <12weeks apart.	6,425 (2.6%)	12 (0.0%)	0 (0.0%)	1,336 (0.8%)	2,284 (0.9%)

For peer review only

Scenario 3b	The woman has two pregnancies close together and the second ends in delivery. If the LMP information is wrong for this pregnancy, then algorithm episodes may overlap.	32,948 (13.1%)	3,705 (1.5%)	1,564 (0.6%)	7,833 (4.9%)	13,464 (5.4%)
Problem 4: The pregnancy is real but is split into separate episodes by the rules of the algorithm						
Scenario 4a	The GP records further information about a pregnancy outcome >25 weeks later for deliveries or >8weeks <12 weeks later for losses.	2,939 (1.2%)	251 (0.1%)	2,646 (1.1%)	2,824 (1.8%)	2,347 (0.9%)
Scenario 4b	The GP records further antenatal information after the end of a pregnancy. Conflicting episodes are generated by the algorithm	43,581 (17.4%)	40,928 (16.3%)	13,531 (5.4%)	16,718 (10.4%)	27,131 (10.8%)
Scenario 4c	The patient has a follow up scan after a pregnancy loss. The scan is recorded in the data as an antenatal scan, a conflicting episode is then generated by the algorithm.	2,734 (1.1%)	0 (0.0%)	0 (0.0%)	744 (0.5%)	2,088 (0.8%)
Scenario 4d	The GP records information about a pregnancy but no outcome with >6 weeks between records. If the second episode has gestational information the start may be assigned before the start of the first episode.	14,695 (5.9%)	14,695 (5.9%)	0 (0.0%)	7,392 (4.6%)	9,911 (3.9%)
Scenario 4e	The pregnancy dates have been shifted backwards by the rules of the algorithm leaving uncovered records. Conflicting episodes are generated by the algorithm.	73,191 (29.2%)	70,472 (28.1%)	53,464 (21.3%)	42,785 (26.7%)	55,205 (22.0%)

For peer review only

None	These pairs of pregnancies did not meet the criteria for any identified scenarios.	35,449 (14.1%)	-	13,241 (5.3%)	14,173 (8.8%)	15,650 (6.2%)

* Denominator = pregnancy episodes which had at least one day overlap with the available HES follow up period and where the woman was eligible for linkage.

**A version of this table restricted to episodes which occurred during practice UTS follow-up and patient's current registration is given in the appendices (Appendix 17)

For peer review only

Discussion

This work has shown that uncertain pregnancy episodes in the CPRD pregnancy register can contain valuable information about a woman's pregnancy. A high proportion of the uncertain episodes were during research quality follow up time and therefore comprise data which would usually be included in study designs (9). We have systematically identified potential reasons for the existence of uncertain episodes within the pregnancy register to allow researchers to consider in more detail whether inclusion is appropriate for their study. This work adds further value to the CPRD Pregnancy Register which is already unique in its inclusion of all pregnancy data regardless of completion (3,4).

We found that most episodes with a missing outcome could be explained by the outcomes not being captured in the CPRD GOLD primary care database; either the patient was not registered at the time of the pregnancy, the outcome was not recorded by the GP but could be found in linked data, or follow up ended before the outcome. These are likely to be genuine and contemporaneous pregnancies which would be missed if episodes with recorded outcome missing were excluded from the Register. In fact, most of the scenarios we identified are consistent with the episodes being true and current pregnancies. When conducting drug utilisation or vaccine uptake studies researchers may wish to include episodes where the database follow-up ended before the outcome to avoid underestimation especially for new drugs or vaccination programs. Table 5 outlines potential considerations for researchers deciding whether to include or exclude uncertain episodes from their study.

There is evidence to suggest that historical outcomes being recorded by the GP during an ongoing pregnancy may explain a sizeable proportion of the uncertain episodes generated by the algorithm. This can lead to true pregnancies being split by the algorithm and depending on the timing this will either generate an additional episode with outcome missing or two separate episodes with outcomes (Figure 1, step 3). In either case the resulting episodes may conflict with one another. Based on our findings this appears to be something that happens fairly frequently. One concern is that these episodes are likely to appear more frequently for women with a history of complicated pregnancy outcomes. For example, previous caesarean sections may be likely to be noted by the GP during current care as would outcomes such as ectopic pregnancies. Researchers should be aware that exclusion of women who have overlapping pregnancies for this reason might therefore systematically exclude those with a history of pregnancy complications, introducing bias.

It is also possible that current pregnancies with serious complications are more likely to have an uncertain episode in the Register. For example, women with pre-eclampsia are more likely to have consultant led antenatal care carried out in hospital increasing the chances that their primary care record is incomplete and has no recorded outcome (10). This data pattern is likely to result in the pregnancy being spilt into multiple episodes without outcome (Figure 1, step 8). Dropping all uncertain episodes at the study design stage may mean that these patients are missed. Researchers who are interested in specific pregnancy complications should take this into consideration and use a tailored approach when selecting a study population.

Whilst some conflicting episodes may be caused by poor quality data there are many conflicting episodes for which it may be possible to clarify which time period is likely to be the true pregnancy. We found that episode conflicts were more likely to occur for pregnancies ending in loss; this is of

For peer review only

little surprise given the wider variation around the true gestation of such pregnancies (11). There was also a large overlap between the conflicting episodes and those that were missing an outcome. Again, this is not surprising as the start and end dates for the missing outcome episodes have large margins of error, given they are often estimated based on one or two antenatal codes (Figure 1, step 8) (3). Not including uncertain episodes may lead to under ascertainment of miscarriage as an outcome. However, including them all may lead to exposure status misclassification due to mis-timed start and end dates or past pregnancy outcomes being counted.

Investigation of the linked HES data has shown that utilising this additional data alongside the Register could help users to identify many missing outcomes (Harron et al., 2016; NHS Digital, 2021; Padmanabhan et al., 2019). Potentially useful pregnancy outcome data was found in multiple places across the HES APC database (NHS Digital, 2021). Identifying outcomes in HES could allow users of the Register to adjust the dates of the pregnancy episodes. Furthermore, utilising the HES DID data to access antenatal scan records offers a useful way to validate the dates of primary care pregnancy episodes as patients are unlikely to have an antenatal scan when they are not currently pregnant (13).

The main limitation of this work is that it relies on the assumption that real life scenarios will consistently result in the same data patterns. EHR data such as CPRD GOLD is not collected for the purposes of research and can be messy for a variety of reasons. The criteria we applied to identify our proposed scenarios may not have been a true fit to each pregnancy episode, this may have resulted in misclassification of the true underlying cause. Whilst we did validate a random sample of pregnancy episodes by looking at the individual Read codes recorded it was not possible to look at every episode in detail. Furthermore, some of our scenarios relied on assumptions as to why and when GPs may record clinical information relating to pregnancy. Whilst this was informed by clinician advice and clinical guidelines it may not be correct in every case. There is also the possibility that there are other scenarios which we did not identify.

We have described in detail reasons why uncertain pregnancy episodes may occur in the CPRD Pregnancy Register and criteria which researchers can apply to ascertain which episodes may fit each scenario. This work offers researchers the opportunity to tailor their study to accommodate these episodes where appropriate (Table 5)

For peer review only

Table 5: Issues with different approaches to dealing with uncertain episodes and recommendations

**Example uses**	**Issues with a Highly Specific Approach: Excluding all uncertain episodes**	**Issues with a Highly Sensitive Approach: Including all uncertain episodes**	**Recommended Tailored approach: Including or excluding uncertain episodes based on scenario criteria.**
Vaccine uptake study	• Underestimate of uptake during pregnancy	• Overestimate of uptake during pregnancy where historic episodes are included	• Consider utilising episodes without recorded outcome which continue after data follow-up.<br>• Consider utilising linked data to obtain additional outcomes.<br>• Exclude episodes which are likely to be derived from historical data.
Drug/Vaccine safety study	• Underestimation of pregnancies ending in loss.<br>• Underestimation of pregnancy complications	• Misclassification of exposure status<br>• Overestimation of outcomes	• Consider utilising linked data to obtain additional outcomes.<br>• Exclude episodes which are likely to be derived from historical data.<br>• Consider merging conflicting episodes which are consistent with problem 4 and adjusting the timing accordingly.

For peer review only

Ascertaining Pregnancy History	• Underestimation of parity.<br>• Underestimation of certain pregnancy events.<br>• Underestimation of pregnancies ending in loss.	• Overestimation of parity.	• Consider utilising linked data to obtain additional outcomes.<br>• Exclude episodes which are likely to be derived from historical data.
Excluding Pregnant Women from a study cohort	• Reduction in potential study population	• Potential misclassification of pregnancy status<br>• Potential errors in pregnancy timing	• Consider merging conflicting episodes which are consistent with problem 4 and adjusting the timing accordingly.<br>• Consider utilising linked data to obtain additional outcomes.<br>• Exclude episodes which are likely to be derived from historical data.

For peer review only

Conclusions

This work has shown evidence that most uncertain pregnancy episodes are consistent with true and current pregnancies for which the data contains valuable information. It is important that researchers carefully consider the impact of including or excluding these episodes from their study. We have demonstrated that examining patterns of events within the primary care data or looking for further evidence in linked data can help to identify possible explanations. Here we offer users of the Pregnancy Register an insight into why these episodes exist and guidance on how to tailor their study population accordingly.

Contributors

JC, KB, ST, RW, HIM and CM contributed to the initiation, planning and design of the study. JC performed the analysis. KB, ST, RW, and CM conducted study supervision. HIM and ST provided clinical input. JC wrote the manuscript with KB, ST, RW, HIM and CM performing critical revision.

Ethics Statement

This study was approved by the Independent Scientific Advisory Committee (ISAC) for Medicines and Healthcare Products Regulatory Agency Database Research (protocol no: 17_285R2 and 19_140). The study was approved by the London School of Hygiene and Tropical Medicine Ethics Committee

Competing Interests

JC and RW are employees of CPRD. There are no other conflicts of interest to report.

Funding

This work forms part of JC's PhD which is funded by CPRD (grant number N/A). KB is funded by a Wellcome Senior Research Fellowship (220283/Z/20/Z). CM was supported by a UKRI Innovation Fellowship at Health Data Research UK London (MR/S003932/1) HIM and ST were funded by the National Institute for Health Research (NIHR) Health Protection Research Unit (HPRU) in Immunisation (IS-HPU1112-10096) at the London School of Hygiene and Tropical Medicine in partnership with Public Health England (PHE). The views expressed are those of the authors and not necessarily those of the NHS, the NIHR, the Department of Health and Social Care, or PHE.

Acknowledgements

This work uses data provided by patients and collected by the NHS as part of their care and support.

For peer review only

Data sharing statement

The data used for this study were obtained from the Clinical Practice Research Datalink (CPRD). All data are available via an application via CPRD's Research Data Governance (RDG) Process (see https://www.cprd.com/research-applications). Data acquisition is associated with a fee and subject to ethics approval.

References

1. National Institute for Health and Care Excellence. Antenatal care for uncomplicated pregnancies. Clin Guidel [Internet]. 2008;(March):1–55. Available from: https://www.nice.org.uk/guidance/cg62/resources/antenatal-care-for-uncomplicated-pregnancies-975564597445

2. CPRD. CPRD: Clinical Practice Research Datalink [Internet]. 2021. Available from: www.cprd.com

3. Minassian C, Williams R, Meeraus WH, Smeeth L, Campbell OMR, Thomas SL. Methods to generate and validate a Pregnancy Register in the UK Clinical Practice Research Datalink primary care database. Pharmacoepidemiol Drug Saf [Internet]. 2019;(April):1–11. Available from: http://doi.wiley.com/10.1002/pds.4811

4. Margulis A V., Palmsten K, Andrade SE, Charlton RA, Hardy JR, Cooper WO, et al. Beginning and duration of pregnancy in automated health care databases: Review of estimation methods and validation results. Pharmacoepidemiology and Drug Safety. 2015.

5. Padmanabhan S. CPRD GOLD Data Specification [Internet]. 2017 [cited 2020 Jul 8]. Available from: https://cprd.com/sites/default/files/CPRD GOLD Full Data Specification v2.0_0.pdf

6. Boggon R, Gallagher A, Williams T van ST. Creating a Mother Baby Link and Pregnancy Register for a UK Population. Pharmacoepidemiol Drug Saf. 2011;20(S44).

7. Padmanabhan S, Carty L, Cameron E, Ghosh RE, Williams R, Strongman H. Approach to record linkage of primary care data from Clinical Practice Research Datalink to other health-related patient data: overview and implications. Eur J Epidemiol [Internet]. 2019;34(1):91–9. Available from: https://doi.org/10.1007/s10654-018-0442-4

8. NHS Digital. Hospital Episode Statistics [Internet]. 2021. Available from: https://digital.nhs.uk/data-and-information/data-tools-and-services/data-services/hospital-episode-statistics

9. Lewis JD, Bilker WB, Weinstein RB, Strom BL. The relationship between time since registration and measured incidence rates in the General Practice Research Database. Pharmacoepidemiol Drug Saf. 2005 Jul;14(7):443–51.

10. National Institute for Health and Care Excellence. Hypertension in pregnancy: pre-eclampsia. 2020.

11. NHS. Miscarriage Overview [Internet]. 2021. Available from: https://www.nhs.uk/conditions/miscarriage/

For peer review only

12. Harron K, Gilbert R, Cromwell D, Van Der Meulen J. Linking data for mothers and babies in de-identified electronic health data. PLoS One [Internet]. 2016;11(10):1–18. Available from: http://dx.doi.org/10.1371/journal.pone.0164667

13.NHS Digital. Diagnostic Imaging Datset [Internet]. 2021. Available from: https://did.hscic.gov.uk/

Figure 1: Pregnancy Register Algorithm Steps used to create the CPRD Pregnancy Register

For peer review only

For peer review only

For peer review only

1. Identify all pregnancy outcome records : All records relating to pregnancy outcomes of any type (live births, stillbirths and early pregnancy losses) are extracted. Records relating to deliveries are considered separately to those relating to early pregnancy losses.

↓

2. Date the first pregnancy outcome: The date of each woman's first pregnancy outcome is estimated using the records identified in step 1 and additional data from linked babies' records (for live births, when available) .

↓

3. Group together records relating to the first pregnancy outcome and characterise the pregnancy outcome: Additional pregnancy outcome records relating to each woman's first outcome which are <= 25 weeks apart for deliveries and <= 8weeks apart for pregnancy losses, are assigned to that outcome. Characteristics of the delivery (pre- or post-term, stillbirth, multiple birth) or the type of early pregnancy loss (miscarriage, induced abortion, ectopic, molar pregnancy or blighted ovum) are determined from these assigned records.

↓

4. Date and characterise each successive pregnancy outcome *:* Steps 2 & 3 are repeated to identify, date and characterise successive pregnancy outcomes sequentially for each woman.

↓

5. Estimate the start of each pregnancy episode *:* Records relating to the timing of the start of pregnancy (first day of last menstrual period) are used to estimate pregnancy start dates. These include information such as records of gestational age, estimated date of delivery and estimated date of conception. In the absence of such data, pregnancy start dates are imputed according to the type of pregnancy outcome (40 weeks when the pregnancy is not flagged as preterm, post-term or multiple , 36 weeks for pre-term pregnancies , 37 weeks for multiple pregnancies, 41 weeks for post term pregnancies).

↓

6. Adjust the start and end dates of a pregnancy *:* Adjustments to pregnancy start and end dates are made either when antenatal records are identified in the 4 weeks before the estimated pregnancy start date (indicating that the initial estimated start date was too late), or when the estimated pregnancy duration exceeds the maximum duration for that type of pregnancy outcome.

↓

7. Assign antenatal records to each pregnancy episode *:* Antenatal records occurring between the start and end date of an identified pregnancy episode are assigned to the pregnancy.

↓

8. Identify additional pregnancies with no recorded outcome *:* All remaining unassigned antenatal records are extracted and categorised into distinct pregnancy episodes. Successive records which are <= 6 weeks apart are grouped together to create a pregnancy. The date of the latest antenatal record in the episode is used as a proxy for the pregnancy end date. The pregnancy start date is estimated using information on gestational age (when available) or by subtracting 4 weeks from the earliest antenatal record in the episode.

Appendix

Appendix 1: Key CPRD GOLD variables

Column name	*Field name*	*Description*
Last Collection Date	lcd	Date of the last collection for the practice
Up to Standard Date	uts	Date at which the practice data is deemed to be of research quality. Derived using a CPRD algorithm that primarily looks at practice death recording and gaps in the data
First Registration Date	frd	Date the patient first registered with the practice.
Current Registration Date	crd	Date the patient's current period of registration with the practice began.
Transfer Out Date	tod	Date the patient transferred out of the practice, if relevant. Empty for patients who have not transferred out
Death Date	deathdate	Patient's date of death – derived using a CPRD algorithm
Acceptable Patient Flag	accept	Flag to indicate whether the patient has met certain quality standards: 1 = acceptable, 0 = unacceptable
Event Date	eventdate	Date associated with the event, as entered by the GP

For peer review only

System Date	sysdate	The date on which information was entered on to the GP software system (generated automatically)

For peer review only

Appendix 2: CPRD Pregnancy Register Variables

Field name	*Description*
Patid	Encrypted unique patient identifier
Pregid	Unique identifier of the pregnancy episode
Mblbabies	Number of babies the pregnancy is linked to in the MBL
babypatid[1]	Encrypted unique patient identifier (linked baby)
babymob	Baby's month of birth as recorded in the baby's medical record
babyyob	Baby's year of birth as recorded in the baby's medical record
totalpregs	Total number of identified pregnancy episodes (per woman)
pregnumber	Pregnancy episode number (per woman)
pregstart	Estimated start date of pregnancy
firstantenatal	Date of earliest antenatal record within the pregnancy

For peer review only

Appendix 3 Example of how a pregnancy may appear in the Register vs GOLD data vs reality

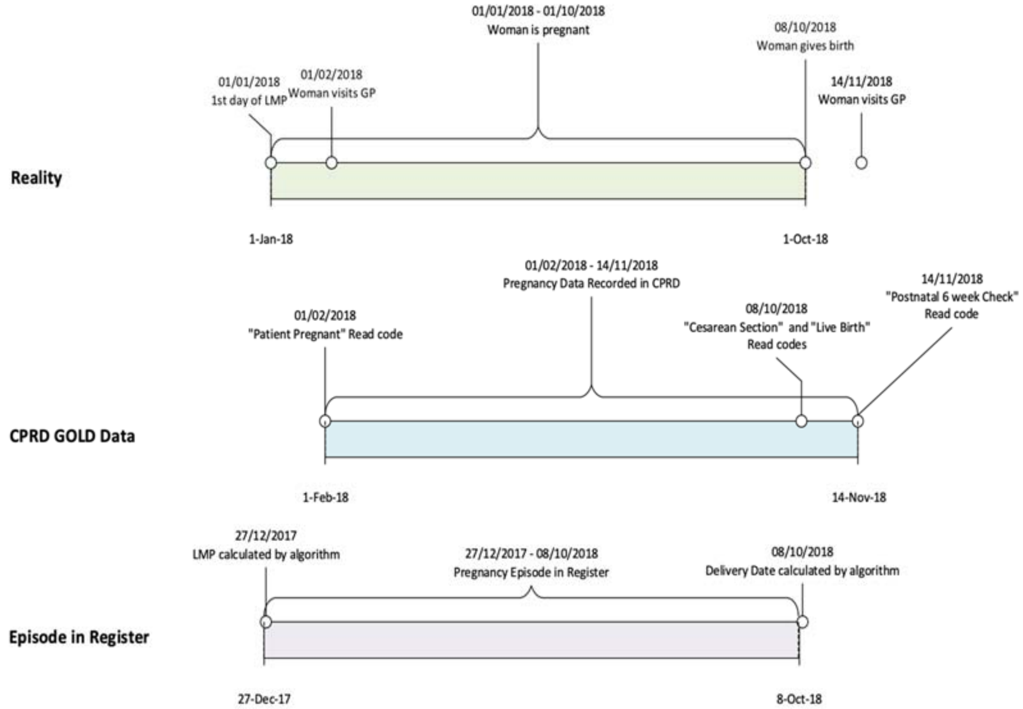

Appendix 4: ICD codes indicating end of pregnancy

O00	O00	Ectopic pregnancy	
O00.0	O000	Abdominal pregnancy	
O00.1	O001	Tubal pregnancy	
O00.2	O002	Ovarian pregnancy	
O00.8	O008	Other ectopic pregnancy	
O00.9	O009	Ectopic pregnancy, unspecified	
O01	O01	Hydatidiform mole	
O01.0	O010	Classical hydatidiform mole	
O01.1	O011	Incomplete and partial hydatidiform mole	
O01.9	O019	Hydatidiform mole, unspecified	
O02	O02	Other abnormal products of conception	
O02.0	O020	Blighted ovum and nonhydatidiform mole	
O02.1	O021	Missed abortion	
O02.8	O028	Other specified abnormal products of conception	
O02.9	O029	Abnormal product of conception, unspecified	
O03	O03	Spontaneous abortion	
O03.0	O030	Spontaneous abortion	Incomplete, complicated by genital tract and pelvic infection
O03.1	O031	Spontaneous abortion	Incomplete, complicated by delayed or excessive haemorrhage
O03.2	O032	Spontaneous abortion	Incomplete, complicated by embolism
O03.3	O033	Spontaneous abortion	Incomplete, with other and unspecified complications
O03.4	O034	Spontaneous abortion	Incomplete, without complication
O03.5	O035	Spontaneous abortion	Complete or unspecified, complicated by genital tract and pelvic infection
O03.6	O036	Spontaneous abortion	Complete or unspecified, complicated by delayed or excessive haemorrhage
O03.7	O037	Spontaneous abortion	Complete or unspecified, complicated by embolism
O03.8	O038	Spontaneous abortion	Complete or unspecified, with other and unspecified complications
O03.9	O039	Spontaneous abortion	Complete or unspecified, without complication

For peer review only

O04	O04	Medical abortion	
O04.0	O040	Medical abortion	Incomplete, complicated by genital tract and pelvic infection
O04.1	O041	Medical abortion	Incomplete, complicated by delayed or excessive haemorrhage
O04.2	O042	Medical abortion	Incomplete, complicated by embolism
O04.3	O043	Medical abortion	Incomplete, with other and unspecified complications
O04.4	O044	Medical abortion	Incomplete, without complication
O04.5	O045	Medical abortion	Complete or unspecified, complicated by genital tract and pelvic infection
O04.6	O046	Medical abortion	Complete or unspecified, complicated by delayed or excessive haemorrhage
O04.7	O047	Medical abortion	Complete or unspecified, complicated by embolism
O04.8	O048	Medical abortion	Complete or unspecified, with other and unspecified complications
O04.9	O049	Medical abortion	Complete or unspecified, without complication
O05	O05	Other abortion	
O05.0	O050	Other abortion	Incomplete, complicated by genital tract and pelvic infection
O05.1	O051	Other abortion	Incomplete, complicated by delayed or excessive haemorrhage
O05.2	O052	Other abortion	Incomplete, complicated by embolism
O05.3	O053	Other abortion	Incomplete, with other and unspecified complications
O05.4	O054	Other abortion	Incomplete, without complication
O05.5	O055	Other abortion	Complete or unspecified, complicated by genital tract and pelvic infection
O05.6	O056	Other abortion	Complete or unspecified, complicated by delayed or excessive haemorrhage
O05.7	O057	Other abortion	Complete or unspecified, complicated by embolism
O05.8	O058	Other abortion	Complete or unspecified, with other and unspecified complications
O05.9	O059	Other abortion	Complete or unspecified, without complication
O06	O06	Unspecified abortion	
O06.0	O060	Unspecified abortion	Incomplete, complicated by genital tract and pelvic infection
O06.1	O061	Unspecified abortion	Incomplete, complicated by delayed or excessive haemorrhage
O06.2	O062	Unspecified abortion	Incomplete, complicated by embolism
O06.3	O063	Unspecified abortion	Incomplete, with other and unspecified complications

O06.4	O064	Unspecified abortion	Incomplete, without complication
O06.5	O065	Unspecified abortion	Complete or unspecified, complicated by genital tract and pelvic infection
O06.6	O066	Unspecified abortion	Complete or unspecified, complicated by delayed or excessive haemorrhage
O06.7	O067	Unspecified abortion	Complete or unspecified, complicated by embolism
O06.8	O068	Unspecified abortion	Complete or unspecified, with other and unspecified complications
O06.9	O069	Unspecified abortion	Complete or unspecified, without complication
O07	O07	Failed attempted abortion	
O07.0	O070	Failed medical abortion, complicated by genital tract and pelvic infection	
O07.1	O071	Failed medical abortion, complicated by delayed or excessive haemorrhage	
O07.2	O072	Failed medical abortion, complicated by embolism	
O07.3	O073	Failed medical abortion, with other and unspecified complications	
O07.4	O074	Failed medical abortion, without complication	
O07.5	O075	Other and unspecified failed attempted abortion, complicated by genital tract and pelvic infection	
O07.6	O076	Other and unspecified failed attempted abortion, complicated by delayed or excessive haemorrhage	
O07.7	O077	Other and unspecified failed attempted abortion, complicated by embolism	
O07.8	O078	Other and unspecified failed attempted abortion, with other and unspecified complications	
O07.9	O079	Other and unspecified failed attempted abortion, without complication	
O08	O08	Complications following abortion and ectopic and molar pregnancy	
O08.0	O080	Genital tract and pelvic infection following abortion and ectopic and molar pregnancy	
O08.1	O081	Delayed or excessive haemorrhage following abortion and ectopic and molar pregnancy	
O08.2	O082	Embolism following abortion and ectopic and molar pregnancy	
O08.3	O083	Shock following abortion and ectopic and molar pregnancy	
O08.4	O084	Renal failure following abortion and ectopic and molar pregnancy	
O08.5	O085	Metabolic disorders following abortion and ectopic and molar pregnancy	
O08.6	O086	Damage to pelvic organs and tissues following abortion and ectopic and molar pregnancy	
O08.7	O087	Other venous complications following abortion and ectopic and molar pregnancy	
O08.8	O088	Other complications following abortion and ectopic and molar pregnancy	
O08.9	O089	Complication following abortion and ectopic and molar pregnancy, unspecified	

For peer review only

Appendix 5: OPCS codes indicating end of pregnancy

OPCS		
P141	INCISION OF INTROITUS OF VAGINA	POSTERIOR EPISIOTOMY AND DIVISION OF LEVATOR ANI MUSCLE
P142	INCISION OF INTROITUS OF VAGINA	POSTERIOR EPISIOTOMY NEC
P143	INCISION OF INTROITUS OF VAGINA	ANTERIOR EPISIOTOMY
Q101	CURETTAGE OF UTERUS	DILATION OF CERVIX UTERI AND CURETTAGE OF PRODUCTS OF CONCEP
Q102	CURETTAGE OF UTERUS	CURETTAGE OF PRODUCTS OF CONCEPTION FROM UTERUS NEC
Q111	OTHER EVACUATION OF CONTENTS OF UTERUS	VACUUM ASPIRATION OF PRODUCTS OF CONCEPTION FROM UTERUS NEC
Q112	OTHER EVACUATION OF CONTENTS OF UTERUS	DILATION OF CERVIX UTERI AND EVACUATION OF PRODUCTS OF CONCE
Q113	OTHER EVACUATION OF CONTENTS OF UTERUS	EVACUATION OF PRODUCTS OF CONCEPTION FROM UTERUS NEC
Q115	OTHER EVACUATION OF CONTENTS OF UTERUS	VACUUM ASPIRATION/PRODUCTS OF CONCEPTION/UTERUS USING RIGID
Q116	OTHER EVACUATION OF CONTENTS OF UTERUS	VACUUM ASPIRATION/PRODUCTS OF CONCEPTION/UTERUS USING FLEXI
Q141	INTRODUCTION OF ABORTIFACIENT INTO UTERINE CAVITY	INTRA-AMNIOTIC INJECTION OF PROSTAGLANDIN
Q142	INTRODUCTION OF ABORTIFACIENT INTO UTERINE CAVITY	INTRA-AMNIOTIC INJECTION OF ABORTIFACIENT NEC
Q143	INTRODUCTION OF ABORTIFACIENT INTO UTERINE CAVITY	EXTRA-AMNIOTIC INJECTION OF PROSTAGLANDIN
Q144	INTRODUCTION OF ABORTIFACIENT INTO UTERINE CAVITY	EXTRA-AMNIOTIC INJECTION OF ABORTIFACIENT NEC
Q145	INTRODUCTION OF ABORTIFACIENT INTO UTERINE CAVITY	INSERTION OF PROSTAGLANDIN PESSARY

For peer review only

Q146	INTRODUCTION OF ABORTIFACIENT INTO UTERINE CAVITY	INSERTION OF ABORTIFACIENT PESSARY NEC
Q148	INTRODUCTION OF ABORTIFACIENT INTO UTERINE CAVITY	OTHER SPECIFIED
Q149	INTRODUCTION OF ABORTIFACIENT INTO UTERINE CAVITY	UNSPECIFIED
R031	SELECTIVE DESTRUCTION OF FETUS	EARLY SELECTIVE FETICIDE
R032	SELECTIVE DESTRUCTION OF FETUS	LATE SELECTIVE FETICIDE
R038	SELECTIVE DESTRUCTION OF FETUS	OTHER SPECIFIED
R039	SELECTIVE DESTRUCTION OF FETUS	UNSPECIFIED
R141	SURGICAL INDUCTION OF LABOUR	FOREWATER RUPTURE OF AMNIOTIC MEMBRANE
R142	SURGICAL INDUCTION OF LABOUR	HINDWATER RUPTURE OF AMNIOTIC MEMBRANE
R148	SURGICAL INDUCTION OF LABOUR	OTHER SPECIFIED
R149	SURGICAL INDUCTION OF LABOUR	UNSPECIFIED
R151	OTHER INDUCTION OF LABOUR	MEDICAL INDUCTION OF LABOUR
R158	OTHER INDUCTION OF LABOUR	OTHER SPECIFIED
R159	OTHER INDUCTION OF LABOUR	UNSPECIFIED
R171	ELECTIVE CAESAREAN DELIVERY	ELECTIVE UPPER UTERINE SEGMENT CAESAREAN DELIVERY
R172	ELECTIVE CAESAREAN DELIVERY	ELECTIVE LOWER UTERINE SEGMENT CAESAREAN DELIVERY
R178	ELECTIVE CAESAREAN DELIVERY	OTHER SPECIFIED
R179	ELECTIVE CAESAREAN DELIVERY	UNSPECIFIED
R181	OTHER CAESAREAN DELIVERY	UPPER UTERINE SEGMENT CAESAREAN DELIVERY NEC
R182	OTHER CAESAREAN DELIVERY	LOWER UTERINE SEGMENT CAESAREAN DELIVERY NEC
R188	OTHER CAESAREAN DELIVERY	OTHER SPECIFIED
R189	OTHER CAESAREAN DELIVERY	UNSPECIFIED

For peer review only

R191	BREECH EXTRACTION DELIVERY	BREECH EXTRACTION DELIVERY WITH VERSION
R198	BREECH EXTRACTION DELIVERY	OTHER SPECIFIED
R199	BREECH EXTRACTION DELIVERY	UNSPECIFIED
R201	OTHER BREECH DELIVERY	SPONTANEOUS BREECH DELIVERY
R202	OTHER BREECH DELIVERY	ASSISTED BREECH DELIVERY
R208	OTHER BREECH DELIVERY	OTHER SPECIFIED
R209	OTHER BREECH DELIVERY	UNSPECIFIED
R211	FORCEPS CEPHALIC DELIVERY	HIGH FORCEPS CEPHALIC DELIVERY WITH ROTATION
R212	FORCEPS CEPHALIC DELIVERY	HIGH FORCEPS CEPHALIC DELIVERY NEC
R213	FORCEPS CEPHALIC DELIVERY	MID FORCEPS CEPHALIC DELIVERY WITH ROTATION
R214	FORCEPS CEPHALIC DELIVERY	MID FORCEPS CEPHALIC DELIVERY NEC
R215	FORCEPS CEPHALIC DELIVERY	LOW FORCEPS CEPHALIC DELIVERY
R218	FORCEPS CEPHALIC DELIVERY	OTHER SPECIFIED
R219	FORCEPS CEPHALIC DELIVERY	UNSPECIFIED
R221	VACUUM DELIVERY	HIGH VACUUM DELIVERY
R222	VACUUM DELIVERY	LOW VACUUM DELIVERY
R223	VACUUM DELIVERY	VACUUM DELIVERY BEFORE FULL DILATION OF CERVIX
R228	VACUUM DELIVERY	OTHER SPECIFIED
R229	VACUUM DELIVERY	UNSPECIFIED
R231	CEPHALIC VAGINAL DELIVERY WITH ABNORMAL PRESENTATION OF	MANIPULATIVE CEPHALIC VAGINAL DELIVERY WITH ABNORMAL PRESENT
R232	CEPHALIC VAGINAL DELIVERY WITH ABNORMAL PRESENTATION OF	NON-MANIPULATIVE CEPHALIC VAGINAL DELIVERY WITH ABNORMAL PRE
R238	CEPHALIC VAGINAL DELIVERY WITH ABNORMAL PRESENTATION OF	OTHER SPECIFIED

For peer review only

R239	CEPHALIC VAGINAL DELIVERY WITH ABNORMAL PRESENTATION OF	UNSPECIFIED
R249	NORMAL DELIVERY	ALL
R251	OTHER METHODS OF DELIVERY	CAESAREAN HYSTERECTOMY
R252	OTHER METHODS OF DELIVERY	DESTRUCTIVE OPERATION TO FACILITATE DELIVERY
R258	OTHER METHODS OF DELIVERY	OTHER SPECIFIED
R259	OTHER METHODS OF DELIVERY	UNSPECIFIED
R271	OTHER OPERATIONS TO FACILITATE DELIVERY	EPISIOTOMY TO FACILITATE DELIVERY
R278	OTHER OPERATIONS TO FACILITATE DELIVERY	OTHER SPECIFIED
R279	OTHER OPERATIONS TO FACILITATE DELIVERY	UNSPECIFIED
R281	INSTRUMENTAL REMOVAL/PRODUCTS/CONCEPTION FROM DEL.UTERU	CURETTAGE OF DELIVERED UTERUS
R288	INSTRUMENTAL REMOVAL/PRODUCTS/CONCEPTION FROM DEL.UTERU	OTHER SPECIFIED
R289	INSTRUMENTAL REMOVAL/PRODUCTS/CONCEPTION FROM DEL.UTERU	UNSPECIFIED
R291	MANUAL REMOVAL/PRODUCTS/CONCEPTION FROM DELIVERED UTERU	MANUAL REMOVAL OF PLACENTA FROM DELIVERED UTERUS
R298	MANUAL REMOVAL/PRODUCTS/CONCEPTION FROM DELIVERED UTERU	OTHER SPECIFIED
R299	MANUAL REMOVAL/PRODUCTS/CONCEPTION FROM DELIVERED UTERU	UNSPECIFIED

For peer review only

R301	OTHER OPERATIONS ON DELIVERED UTERUS	REPOSITIONING OF INVERTED DELIVERED UTERUS
R302	OTHER OPERATIONS ON DELIVERED UTERUS	EXPRESSION OF PLACENTA
R303	OTHER OPERATIONS ON DELIVERED UTERUS	INSTRUMENTAL EXPLORATION OF DELIVERED UTERUS NEC
R304	OTHER OPERATIONS ON DELIVERED UTERUS	MANUAL EXPLORATION OF DELIVERED UTERUS NEC
R308	OTHER OPERATIONS ON DELIVERED UTERUS	OTHER SPECIFIED
R309	OTHER OPERATIONS ON DELIVERED UTERUS	UNSPECIFIED
R321	REPAIR OF OBSTETRIC LACERATION	REPAIR OF OBSTETRIC LACERATION OF UTERUS OR CERVIX UTERI
R322	REPAIR OF OBSTETRIC LACERATION	REPAIR OF OBSTETRIC LACERATION OF PERINEUM AND SPHINCTER
R323	REPAIR OF OBSTETRIC LACERATION	REPAIR OF OBSTETRIC LACERATION OF VAGINA AND FLOOR OF PELVIS
R324	REPAIR OF OBSTETRIC LACERATION	REPAIR OF MINOR OBSTETRIC LACERATION
R325	REPAIR OF OBSTETRIC LACERATION	REPAIR OBSTETRIC LACERATION PERINEUM SPHINCTER MUCOSA ANUS
R328	REPAIR OF OBSTETRIC LACERATION	OTHER SPECIFIED
R329	REPAIR OF OBSTETRIC LACERATION	UNSPECIFIED

For peer review only

Appendix 6: HES Maternity Values to indicate delivery

Variable	*Definition*	*Acceptable values*
numbaby	Number of babies delivered	1-4
delmeth	Method used to deliver a baby that is a registrable birth	0-9
delplac	Actual type of delivery place	0-8
delprean	Anaesthetic or analgesic administered before and during labour and delivery	1-7
delposan	Anaesthetic or analgesic administered after delivery	1-7
neodur	Baby's age in days	>=1
neocare	Neonatal level of care	0-3
postdur	Postnatal days of stay	>=1

For peer review only

Appendix 7: Pregnancy Read codes identified as likely to be recorded as useful pregnancy history

medcode	read_oxmis_code	read_oxmis_term
164	635..13	Premature baby
165	L04..11	Miscarriage
255	L05..12	Termination of pregnancy
364	7F13111	Lower uterine segment caesarean section (LSCS) NEC
618	L398400	Delivery by emergency caesarean section
683	Q420.00	Haemolytic disease due to rhesus isoimmunisation
720	L398.00	Caesarean delivery
740	7F12.00	Elective caesarean delivery
863	L398200	Caesarean section - pregnancy at term
974	Q4z..15	Stillbirth NEC
1413	L264.00	Intrauterine death
1492	L36..00	Postpartum haemorrhage (PPH)
1744	L03..00	Ectopic pregnancy
2240	Q4z..12	Neonatal death
2638	L1...00	Pregnancy complications
2639	E204.11	Postnatal depression
2664	L180900	Gestational diabetes mellitus
2787	L11..11	Antepartum haemorrhage
2923	62T1.00	Puerperal depression
2924	7E06600	Hysterotomy and termination of pregnancy
3029	L166500	Infections of kidney in pregnancy
3085	7F12z00	Elective caesarean delivery NOS
3327	L13..11	Hyperemesis gravidarum
3874	L031200	Tubal abortion
4367	L362.00	Secondary and delayed postpartum haemorrhage
4530	L00..00	Hydatidiform mole
4607	L414.00	Postnatal deep vein thrombosis

For peer review only

4638	7F13.00	Other caesarean delivery
4786	L213200	Multiple delivery, all by caesarean section
4979	Eu53012	[X]Postpartum depression NOS
5113	L39y411	Postnatal vaginal discomfort
5464	L11y100	Other antepartum haemorrhage - delivered
7174	L43..00	Obstetric pulmonary embolism
7670	L398z00	Caesarean delivery NOS
7916	Z254500	Delivered by caesarean section - pregnancy at term
8147	L264.11	Fetal death in utero
8295	Q48D100	[X]Macerated stillbirth
8446	L180811	Gestational diabetes mellitus
8776	Q48D.00	[X] Stillbirth
8906	ZV27.12	[V]Stillbirth
9067	L125.00	Severe pre-eclampsia
9668	7F12100	Elective lower uterine segment caesarean delivery
9800	L398300	Delivery by elective caesarean section
10049	7F12111	Elective lower uterine segment caesarean section (LSCS)
10278	L180800	Diabetes mellitus arising in pregnancy
11359	L180.00	Diabetes mellitus during pregnancy/childbirth/puerperium
11947	L181500	Postpartum thyroiditis
11986	7E13300	Excision of ruptured ectopic tubal pregnancy
12090	L126.00	Eclampsia
12118	7F13300	Emergency caesarean section
12320	L09..11	Complications following abortion/ectopic/molar pregnancies
13307	Eu53011	[X]Postnatal depression NOS
13584	3885	Edinburgh postnatal depression scale
15061	L13..12	Hyperemesis of pregnancy
15514	7F13000	Upper uterine segment caesarean delivery NEC
15533	L451400	Obstetric breast abscess with postnatal complication

For peer review only

16250	L414.12	Phlegmasia alba dolens - obstetric
16281	L45z400	Obstetric breast infection NOS with postnatal complication
16321	L360.00	Third-stage postpartum haemorrhage
17614	Eu53111	[X]Puerperal psychosis NOS
17744	7F13100	Lower uterine segment caesarean delivery NEC
18258	L167.00	Liver disorder in pregnancy
18369	ZV27100	[V]Single stillbirth
18702	6G00.00	Postnatal depression counselling
18770	Q20yz13	Renal injury due to birth trauma
18830	L414.11	DVT - deep venous thrombosis, postnatal
20152	L090y00	Sepsis NOS following abortion/ectopic/molar pregnancy
20165	L363.00	Postpartum coagulation defects
20307	L091.00	Delayed/excessive haemorrhage following abortive pregnancy
20573	Q48D000	[X]Fresh stillbirth
22775	L11y.00	Other antepartum haemorrhage
23015	6334	Twins - 1 still + 1 live born
23588	L414200	Postnatal deep vein thrombosis with postnatal complication
23642	Eu53z00	[X]Puerperal mental disorder, unspecified
24089	L356z00	Obstetric damage to pelvic joints and ligaments NOS
24927	Eu53.00	[X]Mental and behav disorders assoc with the puerperium NEC
24951	L18C.00	Endocrine nutrition+metab dis complic pregn,childbirth+puerp
25028	L09z.00	Complication NOS following abortion/ectopic/molar pregnancy
25415	Q411.00	Perinatal intraventricular haemorrhage
28364	Q420.12	Rhesus isoimmunisation of the newborn
28861	L398500	Delivery by caesarean hysterectomy
29155	7F1A000	Caesarean hysterectomy

For peer review only

31203	6332	Single stillbirth
31857	Q204.00	Spine or spinal cord injury due to birth trauma
32950	L03y100	Cornual pregnancy
33477	L398100	Caesarean delivery - delivered
33724	L03z.00	Ectopic pregnancy NOS
34136	L120z00	Benign essential hypertension in preg/childb/puerp NOS
34173	L12B.00	Proteinuric hypertension of pregnancy
34299	L240.00	Congenital abnormality of uterus in preg/childbirth/puerp
34502	6335	Twins - both still born
34639	L180100	Diabetes mellitus during pregnancy - baby delivered
34868	L4...00	Complications of the puerperium
35190	7F13z00	Other caesarean delivery NOS
35309	6755	Post miscarriage counselling
36421	L167z00	Liver disorder in pregnancy NOS
37280	L36z.00	Postpartum haemorrhage NOS
39117	L126500	Eclampsia in pregnancy
40224	Eu53000	[X]Mild mental/behav disorder assoc with the puerperium NEC
40500	Eu53100	[X]Severe mental and behav disorder assoc wth puerperium NEC
40730	L125z00	Severe pre-eclampsia NOS
42088	L125100	Severe pre-eclampsia - delivered
42598	L175.00	Maternal rubella in pregnancy, childbirth and the puerperium
44494	L441z00	Caesarean wound disruption NOS
45806	L070x00	Unspecified abortion with complication NOS
46756	L184.00	Mental disorders in pregnancy, childbirth and the puerperium
47227	ZV27300	[V]Twins, one live born and one stillborn
47542	L362200	Secondary postpartum haemorrhage with postnatal problem

For peer review only

47546	7F12y00	Other specified elective caesarean delivery
47607	L440.11	CVA - cerebrovascular accident in the puerperium
47686	L181.00	Thyroid dysfunction in pregnancy/childbirth/puerperium
47741	L127000	Pre-eclampsia or eclampsia with hypertension unspecified
47863	Lyu5200	[X]Other single delivery by caesarean section
48500	Q49..00	Cardiovascular disorders originating in the perinatal period
49363	Q200100	Subdural haemorrhage unspecified, due to birth trauma
50093	L093000	Oliguria following abortive pregnancy
52875	L398000	Caesarean delivery unspecified
52967	Lyu0B00	[X]Complic following abortion & ectopic & molar preg, unspec
53141	L241.00	Tumour of uterine body in pregnancy/childbirth/puerperium
54652	L362z00	Secondary and delayed postpartum haemorrhage NOS
55304	L131z00	Hyperemesis gravidarum with metabolic disturbance NOS
56279	L440.12	Stroke in the puerperium
57236	L400200	Puerperal endometritis with postnatal complication
58156	L03y.00	Other ectopic pregnancy
58982	L186.00	Other cardiovascular diseases in pregnancy/childbirth/puerp
61204	L414z00	Postnatal deep vein thrombosis NOS
61578	L441000	Caesarean wound disruption unspecified
62052	L092500	Uterus damage following abortive pregnancy
62358	L167000	Liver disorder in pregnancy unspecified
62919	L125200	Severe pre-eclampsia - delivered with postnatal complication
63277	L393.00	Acute renal failure following labour and delivery
64127	L121000	Renal hypertension in pregnancy/childbirth/puerp unspecified
64384	L180z00	Diabetes mellitus in pregnancy/childbirth/puerperium NOS
66213	Q20yz12	Kidney injury due to birth trauma

For peer review only

66594	L186.11	Heart disease during pregnancy
67006	L096400	Pulmonary embolism following abortive pregnancy
68319	L351300	Rupture of uterus during/after labour with postnatal problem
70891	L126400	Eclampsia with postnatal complication
71314	L093.00	Renal failure following abortive pregnancy
71717	L121100	Renal hypertension in pregnancy/childbirth/puerp - delivered
72215	L241z00	Uterine body tumour in pregnancy/childbirth/puerperium NOS
72230	L241100	Tumour of uterine body - baby delivered
72458	L393000	Post-delivery acute renal failure unspecified
72513	7F13200	Extraperitoneal caesarean section
73407	L261200	Rhesus isoimmunisation with antenatal problem
73617	L261000	Rhesus isoimmunisation unspecified
73647	L188000	Abnormal GTT - unspec whether during pregnancy/puerperium
86756	Qyu3600	[X]Other chronic resp diseases originating/perinatal period
93710	Q317y00	Other specified perinatal chronic respiratory disease
94718	L121z00	Renal hypertension in pregnancy/childbirth/puerperium NOS
97367	L43z100	Obstetric pulmonary embolism NOS - delivered
99188	L173.00	Maternal tuberculosis in pregnancy/childbirth/puerperium
103465	Qyu3B00	[X]Cardiovasc disord origin in the perinat period, unspecif
103677	Eu32B00	[X]Antenatal depression
110868	L181000	Thyroid dysfunction - unspec whether in pregnancy/puerperium
111574	L114z00	Antepartum haemorrhage with trauma NOS

For peer review only

Appendix 8: Antenatal Read codes identified as pregnancy advice codes

medcode	read_oxmis_code	read_oxmis_term
30351	67A6.00	Drugs in pregnancy advice
36903	67AZ.00	Pregnancy advice NOS
102359	67AF.00	Pregnancy advice for patients with epilepsy
107892	67Iu.00	Advice on risk harm to fetus from maternl medictn dur preg
110888	67It.00	Advice on risk harm to mother from maternl medictn dur preg

For peer review only

startsource	Data source used to estimate pregnancy start date: 1 = Imputed[2], 2 = EDD, 3 = LMP, 4 = Gestational age at birth, 5 = Gestational age from antenatal record, 6 = EDC
startadj	Flag to indicate whether the pregnancy start date has been adjusted: 0 = Not adjusted, 1 = Due to antenatal records in the preceding 4 weeks, 2 = Due to specific conflicts between the estimated pregnancy duration and records indicating gestational age at birth (live births and stillbirths only), 3 = Both
Secondtrim[3]	Estimated start date of second trimester
Thirdtrim[3]	Estimated start date of third trimester
pregend	Estimated end date of pregnancy. NB: For pregnancies with unknown outcome, the date of the latest antenatal record in the pregnancy episode is provided.
endsource	Data source used to estimate pregnancy end date: 1 = Delivery record, 2 = Postnatal record in the mother's medical record, 3 = Discharge date relating to a delivery, 4 = Baby's (month and) year of birth as recorded in the baby's medical record, 5 = Postnatal record in the baby's medical record, 6 = First consultation in the baby's medical record. Only completed for live births and stillbirths.
endadj	Flag to indicate whether the pregnancy end date has been adjusted: 0 = Not adjusted, 1 = Due to specific conflicts between the estimated pregnancy duration and records indicating gestational age, 2 = Due to prior adjustments to the start date, 3 = Both. Missing for deliveries based on late pregnancy records[4].
gestdays	Estimated duration of pregnancy episode in days (calculated as pregend minus pregstart)

For peer review only

matage	Mother's age at end of pregnancy (years)
outcome	Outcome of pregnancy: 1 = Live birth, 2 = Stillbirth, 3 = 1 and 2, 4 = Miscarriage, 5 = TOP, 6 = Probable TOP, 7 = Ectopic, 8 = Molar, 9 = Blighted ovum, 10 = Unspecified loss, 11 = Delivery based on a third trimester pregnancy record, 12 = Delivery based on a late pregnancy record[4], 13 = Outcome unknown
preterm_ev	Flag to indicate evidence of a premature delivery: 1=preterm, 0=no evidence of preterm, 9=not applicable (outcome not a delivery)
postterm_ev	Flag to indicate evidence of a post-term delivery: 1=post-term, 0=no evidence of post-term, 9=not applicable (outcome not a delivery)
multiple_ev	Flag to indicate evidence of a multiple pregnancy: 1=multiple, 0=no evidence of multiple. Missing for pregnancy losses.
conflict	Flag to indicate whether the pregnancy episode overlaps with another episode (within a woman): 1=conflicting, 0= non-conflicting

1 A single babypatid is provided. For multiple pregnancies resulting in >1 liveborn infant (when mblbabies>1), additional babypatids may be retrieved from the MBL.

2 For "Outcome unknown" pregnancies, the imputed start date is obtained by subtracting 4 weeks from the earliest antenatal record in the episode.

3 The timing of trimesters is estimated using a common convention: first trimester (first day of LMP [pregstart] to 13 completed weeks), second (weeks 14 to 26), and third (week 27 to delivery [pregend]).

4 Late pregnancy records refer to the period up to 3 weeks before delivery, e.g. "Baby overdue".

For peer review only

Appendix 9: Read codes potentially misclassified as antenatal rather than outcomes

medcode	read_oxmis_code	read_oxmis_term
424	L281.00	Premature rupture of membranes
906	L100.00	Threatened abortion
1413	L264.00	Intrauterine death
1737	L02..00	Missed abortion
1879	L071.00	Unspecified abortion incomplete
3004	L14..11	Premature labour
6730	L051.12	Surgical abortion - incomplete
7114	L044.00	Inevitable abortion incomplete
7413	L041.00	Spontaneous abortion incomplete
8076	8H7W.00	Refer to TOP counselling
8147	L264.11	Fetal death in utero
8173	L043.00	Inevitable abortion unspecified
12241	L02..11	Missed miscarriage
12337	L051.00	Legal abortion incomplete
17625	L044.11	Inevitable miscarriage incomp
20621	ZV25313	[V]Admission for termination of pregnancy
20809	L14..00	Early or threatened labour
20933	6776	Preg. termination counselling
25883	L071y00	Unspecified incomplete abortion + no mention of complication
28605	L051z00	Incomplete legal abortion NOS
29439	L041z00	Incomplete spontaneous abortion NOS
33964	L0A4.00	Failed medical abortion, without complication
35184	L071z00	Unspecified incomplete abortion NOS
35273	L097.00	Readmission for abortive pregnancy (NHS codes)
35701	L100000	Threatened abortion unspecified
37831	L264z00	Intrauterine death NOS

For peer review only

39754	L051.11	Medal abortion - incomplete
41118	L08z.00	Failed attempted abortion NOS
41783	L041100	Incomp spontaneous abortion + delayed/excessive haemorrhage
47376	L0A1.00	Failed medical abortion complic by genital tract/pelvic infn
47435	L097200	Readmission for retained produc of concept, illegal abortion
50903	L0A2.00	Failed medical abortion comp by delayed/excessive haem'ge
53201	ZV25B00	[V]Admission for administration of abortifacient
59572	L0A3.00	Failed medical abortion, complicated by embolism
59789	L14z.00	Early or threatened labour NOS
65716	Q011.00	Fetus/neonate affected maternal premature rupture membrane
68683	7E0B.00	Introduction of abortifacient into uterine cavity
96418	L06z.00	Illegally induced abortion NOS
97391	L281200	Premature rupture of membranes with antenatal problem
99205	7E0Bz00	Introduction of abortifacient into uterine cavity NOS
101959	7E0B300	Extraamniotic injection of abortifacient NEC
102362	389B.00	Assessment for termination of pregnancy
102494	8Hh3.00	Self referral to termination of pregnancy service
105048	7E0By00	Introduction of abortifacient into uterine cavity OS

For peer review only

Appendix 10: Outcome Groupings

Pregnancy Outcomes will be grouped together with those pregnancies which would have similar rules applied and combinations of outcome group for each pair will be coded.

Group	*Pregnancy Register codes*	*Group*
Early Pregnancy Loss	4, 5, 6, 10, 7, 8, 9	1
Delivery	1, 2, 3, 11, 12	2
Unknown Outcome	13	3

For peer review only

Appendix 11: Read Codes identified as likely to only be recorded during current pregnancy

medcode	read_oxmis_term
30979	[SO]Fetus
36441	[V]Amniocentesis to screen for chromosomal anomalies
61455	[V]Amniotic fluid to screen for alphafetoprotein levels
6298	[V]Antenatal screening
49665	[V]Antenatal screening for chromosomal anomalies
35912	[V]Pregnancy confirmed
43428	[V]Screening for fetal growth retardation using ultrasonics
103341	[V]Screening for isoimmunisation
7536	[V]Screening for malformations using ultrasonics
13167	A/N 12 weeks examination
13166	A/N 16 week examination
29364	A/N 20 week examination
13169	A/N 24 week examination
26554	A/N 28 week examination
29627	A/N 30 week examination
13171	A/N 32 week examination
13170	A/N 34 week examination
29727	A/N 35 week examination
29610	A/N 36 week examination
26552	A/N 37 week examination
26553	A/N 38 week examination
26551	A/N 39 week examination
29280	A/N 40 week examination
37029	A/N 41 week examination
55605	A/N 42 week examination
3517	A/N booking examination
13984	Antenatal ultrasound confirms ectopic pregnancy
12260	A/N Rh antibody screen

For peer review only

68089	A/N Rh antibody screen NOS
70616	A/N sickle cell screen done
102099	A/N sickle cell screen NOS
64141	A/N syphilis screen-blood sent
14086	A/N U/S scan abnormal
27057	A/N U/S scan for ? abnormality
64537	A/N U/S scan for slow growth
37221	A/N U/S scan normal +? dates
35826	A/N U/S scan normal += dates
106588	Antenatal 22 week examination
106923	Antenatal 25 week examination
106425	Antenatal 31 week examination
13168	Antenatal examination NOS
10056	Antenatal examinations
13416	Antenatal sickle cell screen
13417	Antenatal syphilis screen
42326	Antenatal syphilis screen NOS
13968	Antenatal ultrasound confirms intra-uterine pregnancy
2029	Antenatal ultrasound scan
27056	Antenatal ultrasound scan at 17-22 weeks
39611	Antenatal ultrasound scan at 22-40 weeks
14084	Antenatal ultrasound scan at 9-16 weeks
14083	Antenatal ultrasound scan NOS
14085	Antenatal ultrasounds scan at 4-8 weeks
12890	Confirmation of pregnancy
50546	Dating scan
9462	Dating/booking US scan
100164	Detailed structural scan
103741	Doppler ultrasound scan of middle cerebral artery of fetus
102885	Doppler ultrasound scan of umbilical artery
95166	Doppler ultrasound scan of uterine artery
46126	Double test
13414	Downs screen - blood test

For peer review only

38358	Downs screen blood test abnormal
34508	Downs screen blood test normal
64832	Downs screening - blood sent
39173	Downs screening blood test NOS
103893	Fetal ascites scan
19720	Fetal monitoring
19590	Fetal movements felt
55493	Fetal movements seen
53420	Fetal tachycardia
9164	Fetal U-S scan
31110	Fundal height equal to dates
25875	Fundal height high for dates
37039	Fundal height low for dates
37038	Girth of pregnant abdomen
91773	Good baseline variability in fetal heart rate
105992	Height of uterine fundus
92171	Mid trimester scan
85992	Non routine obstetric scan for fetal observations
95875	Non routine obstetric scan for fetal observations NOS
38846	Normal fetal heart baseline pattern
13997	Nuchal scan
95881	O/E - fetal heart < 40
101119	O/E - fetal heart > 200
68996	O/E - fetal heart 100-120
26707	O/E - fetal heart 120-160
62903	O/E - fetal heart 160-180
62898	O/E - fetal heart 180-200
72837	O/E - fetal heart 40-80
70856	O/E - fetal heart 80-100
7681	O/E - fetal heart heard
22815	O/E - fetal movements
25153	O/E - fetal movements felt
52857	O/E - fetal movements NOS
53687	O/E - fetal movements seen

For peer review only

27801	O/E - fetal movemnt.diminished
26710	O/E - fetal presentation
67186	O/E - fetal presentation NOS
69819	O/E - fetal station NOS
24701	O/E - fetus very active
26708	O/E - fundal size = dates
37049	O/E - fundus = term size
26705	O/E - fundus 12-16 week size
37051	O/E - fundus 16-20 week size
26704	O/E - fundus 20-24 week size
26709	O/E - fundus 24-28 week size
30802	O/E - fundus 28-32 week size
30803	O/E - fundus 32-34 week size
26703	O/E - fundus 34-36 week size
26706	O/E - fundus 36-38 week size
13318	O/E - fundus size - obstetric
30804	O/E - gravid uterus size
62897	O/E - gravid uterus size NOS
37180	O/E - lie of fetus
29788	O/E - multiple presentation
63024	O/E -fetal presentation unsure
37050	O/E -fundus 38 weeks-term size
49519	Observation of position of pregnancy
12625	Obstetric monitoring
44173	Obstetric X-ray - fetus
56727	Obstetric X-ray - placenta
85951	Other non routine obstetric scan NOS
96343	Other specified routine obstetric scan
13165	Patient currently pregnant
127	Patient pregnant
14899	Patient pregnant NOS
38669	Placenta U-S scan
9986	Pregnancy care
4536	Pregnancy confirmed

For peer review only

15338	Pregnancy unplanned ? wanted
14877	Pregnant - ? planned
30817	Pregnant - blood test confirms
51298	Pregnant - on abdom. palpation
20240	Pregnant - planned
16215	Pregnant - urine test confirms
35592	Pregnant - V.E. confirms
10173	Pregnant abdomen observation
15567	Pregnant -unplanned-not wanted
107698	Pregnant uterus displaced laterally
32975	Pregnant, diaphragm failure
29692	Pregnant, IUD failure
14994	Pregnant, sheath failure
11989	Referral for termination of pregnancy
2278	Requests pregnancy termination
69815	Rh screen - 1st preg. sample
29623	Rh screen - 2nd preg. sample
109416	Rh screen - 3rd preg. sample
93946	Rhesus detailed scan
86011	Routine obstetric scan
85245	Routine obstetric scan NOS
6095	Seen in antenatal clinic
29205	Serum pregnancy test positive
70845	Sinusoidal pattern of fetal heart
27614	Triple test
39218	Ultrasonic doppler for fetal heart sounds
19800	Ultrasound in obstetric diagn.
12837	Ultrasound monitoring of early pregnancy
13965	Ultra-sound scan - obstetric
3030	Urine pregnancy test positive
2382	U-S obstetric diagn. scan NOS
29685	U-S obstetric scan abnormal
4797	U-S obstetric scan normal
45963	U-S scan - fetal abnormality

For peer review only

72159	U-S scan - fetal cephalometry
42093	U-S scan - fetal maturity
41919	U-S scan - fetal presentation
41937	U-S scan - multiple fetus
35558	U-S scan - obstetric, diagn.
68858	U-S scan -placental localisatn
67047	Viability scan
37147	Viability US scan
10306	Weeks pregnant

For peer review only

Appendix 12: Outcome Group Combinations

Within conflicting pairs combinations of outcome groups will be coded as follows:

Outcome Group combination	*Variable Code*
1 1 (Loss- Loss)	1
1 2 (Loss- Delivery)	2
1 3 (Loss- Unknown)	3
2 2 (Delivery- Delivery)	4
2 3 (Delivery- Unknown)	5
3 3 (Unknown- Unknown)	6

For peer review only

Appendix 13: Read codes for Antenatal scan

medcode	read_oxmis_code	Read term
2029	62G..00	Antenatal ultrasound scan
13965	584..13	Ultra-sound scan - obstetric
9462	584A.00	Dating/booking US scan
2382	584Z.00	U-S obstetric diagn. scan NOS
13997	584G.00	Nuchal scan
42093	5846	U-S scan - fetal maturity
37147	584B.00	Viability US scan
4797	5842	U-S obstetric scan normal
27019	5841	U-S obstetric scan requested
9164	584..11	Fetal U-S scan
14083	62GZ.00	Antenatal ultrasound scan NOS
35826	62G6.00	A/N U/S scan normal += dates
14084	62GC.00	Antenatal ultrasound scan at 9-16 weeks
35558	584..12	U-S scan - obstetric, diagn.
50546	7F26000	Dating scan
29012	7F27300	Nuchal translucency scan
27056	62GD.00	Antenatal ultrasound scan at 17-22 weeks
39611	62GE.00	Antenatal ultrasound scan at 22-40 weeks
47415	62G5.00	A/N U/S scan awaited
37220	62G2.00	A/N U/S scan offered
14085	62GB.00	Antenatal ultrasounds scan at 4-8 weeks
29685	5843	U-S obstetric scan abnormal
72159	5845	U-S scan - fetal cephalometry
45963	5847	U-S scan - fetal abnormality
27057	62G9.00	A/N U/S scan for ? abnormality

For peer review only

41919	5849	U-S scan - fetal presentation
30885	62G4.00	A/N U/S scan wanted
86011	7F26.00	Routine obstetric scan
68858	5844	U-S scan -placental localisatn
67047	7F26100	Viability scan
41937	5848	U-S scan - multiple fetus
14086	62G8.00	A/N U/S scan abnormal
85992	7F27.00	Non routine obstetric scan for fetal observations
37221	62G7.00	A/N U/S scan normal +? dates
38669	5844.11	Placenta U-S scan
78449	7F28.00	Other non routine obstetric scan
100164	7F27100	Detailed structural scan
92171	7F26200	Mid trimester scan
95166	7F2A111	Doppler ultrasound scan of uterine artery
64537	62GA.00	A/N U/S scan for slow growth
47116	7F28000	Placental localisation scan
85245	7F26z00	Routine obstetric scan NOS
102885	7F2A011	Doppler ultrasound scan of umbilical artery
96343	7F26y00	Other specified routine obstetric scan
95875	7F27z00	Non routine obstetric scan for fetal observations NOS
85951	7F28z00	Other non routine obstetric scan NOS
98261	7F27y00	OS non routine obstetric scan for fetal observations
95698	7F28y00	Other specified other non routine obstetric scan

For peer review only

Appendix 14: DID Snomed foetal scan codes

Dating/booking ultrasound scan (procedure)	169229007
Fetal anatomy study (procedure)	271442007
Fetal biophysical profile (procedure)	21623001
Fetal echocardiography (procedure)	433235006
Magnetic resonance imaging of multiple pregnancy (procedure)	450825001
Placental localization (procedure)	164817009
Ultrasonography of multiple pregnancy for fetal anomaly (procedure)	445866007
Ultrasonography of multiple pregnancy for fetal nuchal translucency (procedure)	446810002
Ultrasound scan for amniotic fluid volume (procedure)	241494004
Ultrasound scan for fetal growth (procedure)	241493005

For peer review only

Appendix 15: Number of episodes with a suitably timed outcome in linked HES data

Dataset in which evidence of a suitably timed pregnancy outcome was found.	N pregnancy episodes where evidence of an outcome was found (% of episodes which were eligible for this linked data source)	N pregnancy episodes which were during current registration and UTS follow up	Total number of pregnancy episodes with recorded outcome missing which were eligible for HES linkage to each source
HES Diagnosis (Part of HES APC)	24,902 (5.9%)	16,389 (65.8%)	424,375
HES Maternity (Part of HES APC)	163,483 (38.5%)	109,393 (66.9%)	424,375
HES Procedures (Part of HES APC)	201,731 (47.5%)	133,077 (66.0%)	424,375
HES Episodes (Part of HES APC)	185,436 (43.7%)	122,350 (66.0%)	424,375
HES Outpatient	735 (0.2%)	560 (76.2%)	311,982
Any HES Source	211,070 (49.7%)	139,084 (65.9%)	424,375

Appendix 16: Numbers of pregnancy episodes with recorded outcome missing which were within practice UTS follow-up and patient's current registration period that were consistent with applied criteria for each scenario

Scenario	Description	N pregnancy episodes which meet this scenario (% of total episodes with missing outcome)	N pregnancy episodes which only meet this scenario (% of the total episodes with missing outcome)	N pregnancy episodes with evidence of an outcome in linked HES (% of linkage eligible episodes)
Denominator		475,664	475,664	265,264
Problem 1: The women was pregnant at the time of the database record, but the outcome was not captured in CPRD primary care data.				
Scenario 1a	The pregnancy outcome occurred in hospital or elsewhere and information wasn't fed back to the practice.	139,084 (29.2%)	1,825 (0.4%)	139,084 (52.4%)
Scenario 1b	The outcome of the pregnancy is recorded in the primary care data but has no event date associated with it.	475 (0.1%)	28 (0.0%)	113 (0.0%)
Scenario 1c	The pregnancy occurred before the patient was registered at the practice or before UTS	-	-	-

For peer review only - http://bmjopen.bmj.com/site/about/guidelines.xhtml

Problem 2: The women was pregnant at the time of the database record, but the pregnancy was still ongoing at the end of available follow up in the database.				
Scenario 2a	The patient transferred out before the putative end of pregnancy	117,571 (24.7%)	34,659 (7.3%)	52,601 (19.8%)
Scenario 2b	The last collection date of the practice was before the putative end of pregnancy	58,698 (12.3%)	20,122 (4.2%)	21,702 (8.2%)
Problem 3: The patient was not pregnant at the time of the database record.				
Scenario 3a	Episode is derived from historical pregnancy information recorded in the first few months after the patient joined the practice	3,875 (0.8%)	386 (0.1%)	1,271 (0.5%)
Scenario 3b	Patient asks for advice whilst planning a pregnancy	0 (0.0%)	0 (0.0%)	0 (0.0%)
Problem 4: The pregnancy record belongs to another pregnancy episode in the Register.				
Scenario 4a	Delay in recording the outcome of a pregnancy, algorithm calculates LMP too late and uncovers records at the beginning of pregnancy creating this PWO.	35,255 (7.4%)	8,265 (1.7%)	14,402 (5.4%)

For peer review only

Scenario 4b	The LMP is derived from the data and is wrong resulting in early codes being uncovered creating this episode	17,110 (3.6%)	3,715 (0.8%)	6,651 (2.5%)
Scenario 4c	The LMP has been shifted backwards uncovering records at the end of the pregnancy	0 (0.0%)	0 (0.0%)	0 (0.0%)
Scenario 4d	A code recorded relating to the patient's delivery history is incorrectly identified by the algorithm as a delivery uncovering records at the end.	219,505 (46.1%)	109,161 (22.9%)	65,883 (24.8%)
Scenario 4e	The outcome of the pregnancy episode has been misclassified as antenatal	18,222 (3.8%)	7,418 (1.6%)	3,990 (1.5%)
Pregnancy Episodes which didn't meet any scenario	These pregnancy episodes did not meet the criteria for any identified scenarios.	94,769 (19.9%)	0 (0.0%)	0 (0.0%)

For peer review only

Appendix 17: Numbers of conflicting pregnancy episodes which were within practice UTS follow-up and patient's current registration period that were consistent with applied criteria for each scenario

Scenario	Description	N pregnancy pairs (% of total conflicting pregnancy pairs)	N which only fit this scenario (% of the total pairs meeting this scenario)	N of pairs with a linked baby in the MBL (% of the total pairs meeting this scenario)	N pairs with evidence of pregnancy in linked HES
Denominator		144,670	144,670	144,670	93,100
Problem 1: Both pregnancies are true but one is a current pregnancy and one is a historical pregnancy					
Scenario 1a	The GP records a past delivery or loss during a current pregnancy with the same outcome resulting in another episode being created	1,981 (1.4%)	317 (0.2%)	1,782 (1.2%)	1,875 (2.0%)
Scenario 1b	A patient has a record relating to a loss recorded during a pregnancy ending in delivery or vice-versa. Conflicting episodes are generated by the algorithm	31,526 (21.8%)	15,453 (10.7%)	8,275 (5.7%)	11,410 (12.3%)
Problem 2: Both pregnancies are historical					

Scenario 2a	A patient has information on historical pregnancies recorded with the current date rather than the actual date.	12,557 (8.7%)	0 (0.0%)	97 (0.1%)	4,309 (4.6%)
Problem 3: Both pregnancies are true and current but the gestation of the second pregnancy estimated by the algorithm is too long.					
Scenario 3a	The woman has two losses which are >8weeks and <12weeks apart.	2,284 (1.6%)	3 (0.0%)	0 (0.0%)	635 (0.7%)
Scenario 3b	The woman has two pregnancies close together and the second ends in delivery. If the LMP information is wrong for this pregnancy, then algorithm episodes may overlap.	13,464 (9.3%)	2,387 (1.6%)	1,113 (0.8%)	4,502 (4.8%)
Problem 4: : The pregnancy is true and current but is split into separate episodes by the rules of the algorithm					
Scenario 4a	The GP records further information about a pregnancy outcome >25 weeks later for deliveries or >8weeks <12 weeks later for losses.	2,347 (1.6%)	183 (0.1%)	2,155 (1.5%)	2,255 (2.4%)

For peer review only

Scenario 4b	The GP records further antenatal information after the end of a pregnancy. Conflicting episodes are generated by the algorithm	27,131 (18.8%)	25,097 (17.3%)	11,097 (7.7%)	11,668 (12.5%)
Scenario 4c	The patient has a follow up scan after a pregnancy loss. The scan is recorded in the data as an antenatal scan, a conflicting episode is then generated by the algorithm.	2,088 (1.4%)	0 (0.0%)	0 (0.0%)	587 (0.6%)
Scenario 4d	The GP records information about a pregnancy but no outcome with >6 weeks between records. If the second episode has gestational information the start may be assigned before the start of the first episode.	9,911 (6.9%)	9,911 (6.9%)	0 (0.0%)	5,079 (5.5%)
Scenario 4e	The pregnancy dates have been shifted backwards by the rules of the algorithm leaving uncovered records. Conflicting episodes are generated by the algorithm.	55,205 (38.2%)	53,044 (36.7%)	43,945 (30.4%)	33,057 (35.5%)
None	These pairs of pregnancies did not meet the criteria for any identified scenarios.	15,650 (10.8%)	-	8,921 (6.2%)	8,235 (8.8%)

For peer review only

Appendix 18: Number of conflicting episode pairs by outcome combination

Outcome Combination	N pairs (% of total conflicting pairs)
two losses	65,826 (26.2%)
one loss one delivery	73,222 (29.2%)
one loss one unknown	62,776 (25.0%)
two deliveries	10,204 (4.1%)
one delivery one unknown	24,303 (9.7%)
two unknowns	14,695 (5.9%)
Total Pairs	251,026 (100%)

For peer review only

BMJ Open

Investigating the Optimal Handling of Uncertain Pregnancy Episodes in the CPRD GOLD Pregnancy Register: a methodological study using UK primary care data

Journal:	*BMJ Open*
Manuscript ID	bmjopen-2021-055773.R1
Article Type:	Original research
Date Submitted by the Author:	25-Nov-2021
Complete List of Authors:	Campbell, Jennifer; Medicines and Healthcare Products Regulatory Agency, Clinical Practice Research Datalink; London School of Hygiene & Tropical Medicine, Faculty of Epidemiology and Population Health, Department of Non-communicable Disease Epidemiology<br>Bhaskaran, Krishnan; London School of Hygiene & Tropical Medicine, Faculty of Epidemiology and Population Health, Department of Non-communicable Disease Epidemiology<br>Thomas, Sara; London School of Hygiene & Tropical Medicine, Faculty of Epidemiology and Population Health, Department of Infectious Disease Epidemiology<br>Williams, Rachael; Medicines and Healthcare Products Regulatory Agency, Clinical Practice Research Datalink<br>McDonald, Helen; London School of Hygiene & Tropical Medicine, Faculty of Epidemiology and Population Health, Department of Infectious Disease Epidemiology<br>Minassian, Caroline ; London School of Hygiene & Tropical Medicine, Faculty of Epidemiology and Population Health, Department of Non-communicable Disease Epidemiology
**Primary Subject Heading**:	Epidemiology
Secondary Subject Heading:	Obstetrics and gynaecology, General practice / Family practice
Keywords:	Maternal medicine < OBSTETRICS, PUBLIC HEALTH, EPIDEMIOLOGY

SCHOLARONE™
Manuscripts

I, the Submitting Author has the right to grant and does grant on behalf of all authors of the Work (as defined in the below author licence), an exclusive licence and/or a non-exclusive licence for contributions from authors who are: i) UK Crown employees; ii) where BMJ has agreed a CC-BY licence shall apply, and/or iii) in accordance with the terms applicable for US Federal Government officers or employees acting as part of their official duties; on a worldwide, perpetual, irrevocable, royalty-free basis to BMJ Publishing Group Ltd ("BMJ") its licensees and where the relevant Journal is co-owned by BMJ to the co-owners of the Journal, to publish the Work in this journal and any other BMJ products and to exploit all rights, as set out in our licence.

The Submitting Author accepts and understands that any supply made under these terms is made by BMJ to the Submitting Author unless you are acting as an employee on behalf of your employer or a postgraduate student of an affiliated institution which is paying any applicable article publishing charge ("APC") for Open Access articles. Where the Submitting Author wishes to make the Work available on an Open Access basis (and intends to pay the relevant APC), the terms of reuse of such Open Access shall be governed by a Creative Commons licence – details of these licences and which Creative Commons licence will apply to this Work are set out in our licence referred to above.

Other than as permitted in any relevant BMJ Author's Self Archiving Policies, I confirm this Work has not been accepted for publication elsewhere, is not being considered for publication elsewhere and does not duplicate material already published. I confirm all authors consent to publication of this Work and authorise the granting of this licence.

For peer review only

Investigating the Optimal Handling of Uncertain Pregnancy Episodes in the CPRD GOLD Pregnancy Register: a methodological study using UK primary care data

Jennifer Campbell [1,2](jennifer.campbell@mhra.gov.uk), Krishnan Bhaskaran[2] (krishnan.bhaskaran@lshtm.ac.uk), Sara Thomas[2] (saralmthomas@gmail.com), Rachael Williams [1] (rachael.williams@mhra.gov.uk), Helen I McDonald[2](helen.mcdonald@lshtm.ac.uk) Caroline Minassian[2](caroline.minassian@lshtm.ac.uk).

1. ***Clinical Practice Research Datalink (CPRD)***
2. ***London School of Hygiene and Tropical Medicine (LSHTM)***

Correspondence to Jennifer Campbell; jennifer.campbell@mhra.gov.uk

Keywords

Pregnancy; Electronic Health Records; United Kingdom; Pregnancy Outcome; Pharmacoepidemiology

Abstract (300 words)

Objectives:

To investigate why episodes of pregnancy identified from electronic health records may be incomplete or conflicting (overlapping) and provide guidance on how to handle them.

Setting:

Pregnancy Register generated from the Clinical Practice Research Datalink (CPRD) GOLD UK primary care database.

Participants:

Female patients with at least one pregnancy episode in the Register (01/01/1937 -31/12/2017) which had no recorded outcome or conflicted with another episode.

Design:

We identified multiple scenarios potentially explaining why uncertain episodes occur. Criteria were established and systematically applied to determine whether episodes had evidence of each scenario. Linked Hospital Episode Statistics were used to identify pregnancy events not captured in primary care

Results:

Of 5.8 million pregnancy episodes in the Register, 932,604 (16%) had no recorded outcome, and 478,341 (8.5%) conflicted with another episode (251,026 distinct conflicting pairs of episodes among 210,593 women). 826,146 (89%) of the episodes without outcome recorded in primary care and 215,577 (86%) of the conflicting pairs were consistent with one or more of our proposed scenarios. For 689,737 (74%) episodes with recorded outcome missing and 215,544 (86%) of the conflicting pairs (at least one episode) supportive evidence (e.g. antenatal records, linked hospital records) suggested they were true and current pregnancies. Furthermore, 516,818 (55 %) and 160,936 (64%) respectively were during research quality follow-up time. For a sizeable proportion of uncertain episode there is evidence to suggest that historical outcomes being recorded by the GP during an ongoing pregnancy may offer explanation (73,208 (29.2%) and 349,874 (37.5%)).

For peer review only - http://bmjopen.bmj.com/site/about/guidelines.xhtml

Conclusions:

This work provides insight to users of the CPRD Pregnancy Register on why uncertain pregnancy episodes exist and indicates that most of these episodes are likely to be real pregnancies. Guidance is given to help researchers consider whether to include/exclude uncertain pregnancies from their studies, and how-to tailor approaches to minimise underestimation and bias.

Strengths and Limitations of this study

- This work carefully examines the way in which pregnancies are recorded in electronic health data in order to maximise its usefulness for pregnancy research.
- Detailed scenarios were developed as to why uncertain pregnancy episodes may occur along with criteria which researchers can apply to ascertain which episodes may fit each scenario.
- Clinician advice and clinical guidelines were used to generate assumptions as to why and when clinicians may record information relating to pregnancy however, these may not be correct in every case.
- Electronic health data is not collected for the purposes of research and can be messy for a variety of reasons, some of which may not have been captured in this study.

Introduction

Understanding how diseases, drugs and other exposures affect pregnant women and their children is an important public health priority. However, pregnant women are excluded from many trials due to potential risks to the woman and her unborn child. Observational research using electronic healthcare records (EHR) has thus become a well-established vital tool for investigating disease prevalence, risk factors and pharmacovigilance in pregnant women. UK primary care databases are particularly useful due to the gate-keeper healthcare system meaning all antenatal care is overseen by a general practitioner (GP) (1). One example of such a database is CPRD GOLD. This database is produced and maintained by the Clinical Practice Research Datalink (CPRD), a government research service collecting de-identified and fully coded patient-level EHR from primary care practices across the UK (2). However, challenges such as incomplete data capture in EHR data can make it difficult to identify accurately the start and end of pregnancies. Recently a collaboration between CPRD and the London School of Hygiene and Tropical Medicine (LSHTM) established a Pregnancy Register of all pregnancies in CPRD GOLD (3) which includes approximately six million estimated pregnancies (henceforth, pregnancies in the Register will be referred to as pregnancy episodes).
Previous approaches to generating pregnancy registers have been limited by the exclusion of pregnancies without identified outcomes and pregnancy records which do not fit chronologically into an identified pregnancy episode (4). Ignoring these records potentially excludes periods when women were pregnant. If these pregnancies systematically differ from those captured more completely, their exclusion may lead to bias. For example, pregnancies ending in miscarriage may be less likely to have the outcome recorded than pregnancies ending in live birth (3). Ignoring pregnancy data which is challenging to interpret may therefore underestimate adverse outcomes. Incomplete capture of pregnancies also impacts descriptive studies that need pregnancies as denominator data, such as vaccine uptake studies. A further limitation of previous approaches is that some women have pregnancies that seemingly overlap in the data, and these are not addressed. These conflicting pregnancies highlight that estimated timings of some pregnancies may

For peer review only - http://bmjopen.bmj.com/site/about/guidelines.xhtml

be suboptimal and/or some pregnancy episodes may not be true pregnancies. Approaches which exclude incongruent or incomplete pregnancy data may lead to misclassification of exposure timings.

The unique advantage of the CPRD Pregnancy Register is that it utilises all pregnancy data in CPRD GOLD, thereby capturing all documented pregnancies regardless of completeness. However, this also presents interpretational challenges: approximately 950,000 pregnancy episodes (16% of all pregnancy episodes) have no outcome recorded and approximately 500,000 pregnancy episodes conflict with another episode for the same woman (episodes identified by the algorithm with at least one day of overlap). These episodes are flagged in the Register enabling researchers to identify them when designing their study. However, there may be multiple reasons for the occurrence of uncertain episodes and therefore absolute rules on whether to include or exclude them from a study may be inappropriate.

We therefore aimed to investigate possible reasons why the algorithm used to generate the CPRD Pregnancy Register identifies uncertain episodes and thus generate information to guide future use of this important resource. Our specific objectives were:

1. To identify potential scenarios which may result in pregnancy episodes without a recorded outcome or those which conflict with another episode for the same woman.
2. To use available data (including linked data) to investigate these potential scenarios and flag pregnancy episodes which are consistent with each one.
3. To provide information to researchers using the Register to help inform their decisions on how to handle these uncertain episodes when designing studies.

Methods

Data Sources

CPRD Primary care data and the Pregnancy Register

The CPRD GOLD UK primary care database contains registration information and all care events that general practice staff record to support clinical care. This includes demographic information (birthyear, sex, etc.), clinical events (signs, symptoms, medical diagnoses), referrals to specialists and secondary care, prescriptions issued in primary care, vaccinations, test results, lifestyle information (e.g. smoking status), and other care administered as part of GP practice (5). CPRD data also contain indicators of data quality at the patient level (known as the acceptability flag Appendix 1) and at the practice level (known as the practice up to standard date Appendix 1). As CPRD GOLD is a longitudinal database, updated monthly, it contains variables indicating whether the patient and practice are still contributing data.

The Pregnancy Register lists and characterises all pregnancies identified in CPRD GOLD based on an algorithm (3). A single record represents a unique pregnancy episode. Each woman may have multiple episodes. Information includes the estimated start and end of pregnancy, its outcome (when recorded) and whether it was a singleton or multiple pregnancy. For live birth pregnancies, patient identifiers of linked babies identified through the CPRD Mother-Baby-Link (MBL) (6) are

For peer review only - http://bmjopen.bmj.com/site/about/guidelines.xhtml

provided. Figure 1 gives an overview of the algorithm steps, including how gestational ages were applied, and Appendix 2 gives a list of the variables provided in the Register. Figure in Appendix 3 shows an example of how a real pregnancy might manifest in (a) raw CPRD gold data, and (b) the processed pregnancy register dataset.

Linked data

Person-level linkage of CPRD primary care data with other datasets (e.g. Hospital Episode Statistics HES) is available for English practices who have consented to participate in the linkage scheme (7). These linkages cover approximately ~56% of contributing CPRD GOLD practices in the UK. Where available we utilised linked data to look for further information about the pregnancy episodes within the Register. HES APC (Admitted Patient Care) data includes information on admission and discharge dates, diagnoses, specialists seen and procedures undertaken for linked patients with a hospitalisation record (8) We searched HES APC data for records of pregnancy outcomes using ICD 10 and OPCS codes (Appendix 4 and 5). HES APC Maternity records were also utilised: a recording of an acceptable value in any of the variables identified as relating to delivery (Appendix 6) was taken as evidence that a delivery had taken place.

The HES Diagnostic Imaging Dataset (DID) provides detailed information about diagnostic imaging tests, including x-rays, MRI scans and foetal growth scans, taken from NHS providers' radiological information systems. This was used for records of foetal scans. Office for National Statistics (ONS) mortality data was also used to ascertain additional death records which may have been missing from CPRD.

We utilised Set 17 of the CPRD linked data for which the coverage periods were: HES APC 01/04/1997-31/07/2017; HES DID 01/04/2012- 31/07/2017; ONS Mortality Data 02/01/1998-19/09/2017.

Study Population

This study included all individuals who had at least one pregnancy episode without a recorded outcome or at least one conflicting pregnancy episode in the February 2018 version of the Pregnancy Register. All pregnancy records for these patients were extracted from the CPRD GOLD database using the pregnancy code-list upon which the pregnancy algorithm is based (3) thereby creating a dataset which included all pregnancy records and the summary Pregnancy Register information for these women. Women were followed-up until the minimum of leaving the practice, death or practice last collection date. In the linked data analysis women with HES records beyond this point were followed-up until the end of linked data coverage.

Identifying scenarios to explain the occurrence of uncertain episodes

Potential scenarios which may result in uncertain pregnancy episodes, including those without recorded outcomes and those which conflicted with another episode, were identified through discussions with the creators of the Register (CM, ST, RW), clinicians and CPRD data experts. The scenarios are based on the structure of the CPRD GOLD data and the Pregnancy Register algorithm (Figure 1, steps 1-8)). The scenarios are not mutually exclusive; thus, episodes may be consistent with more than one scenario.

Pregnancy Episodes with no recorded outcome

For peer review only - http://bmjopen.bmj.com/site/about/guidelines.xhtml

Scenarios with the potential to result in episodes with missing outcomes were identified. There are four overarching problems with various specific scenarios within them: the pregnancies are true and current , but the outcome was not captured in CPRD primary care data; the pregnancies are true and current , but the pregnancy was still ongoing at the end of follow up in the database; the patient was not pregnant at the time of the database record; the pregnancy is really part of another pregnancy episode in the Register. The twelve scenarios which fall under these problems are described in Table 1a.

Conflicting Pregnancy Episodes

Scenarios with the potential to result in conflicting episodes were proposed and are described in detail in Table 1b. Identifying the scenarios was an iterative process, after applying initial scenarios we took a sample of 50 conflicting pregnancy episodes and reviewed the patient data. This allowed us to validate existing scenarios and identify further scenarios. Scenarios can be grouped under four overarching problems: both pregnancies are true but one is a historical pregnancy; both pregnancies are historical; both pregnancies are true and current but the gestation of the second pregnancy estimated by the algorithm is too long; the woman was pregnant, but one pregnancy has been split into multiple episodes by the rules of the algorithm (Appendix 3).

For peer review only

For peer review only - http://bmjopen.bmj.com/site/about/guidelines.xhtml

Table 1a: Description of potential scenarios leading to pregnancy episodes with no recorded outcome and scenario criteria applied

**Scenario**	**How does this appear in the data?**	**Criteria used to determine if there is evidence in the data that an episode is consistent with the scenario in question**
Problem 1: The women was pregnant at the time of the database record, but the outcome ***was not captured in CPRD primary care data.***		
1a. The woman was pregnant. She had a delivery, miscarriage, or termination of pregnancy (TOP) in hospital or elsewhere and information either was not fed back to the general practice, or was fed back but not coded in the woman's records	There will be no evidence of an outcome in CPRD data up to 38 weeks* (for delivery) or up to 20 weeks (for miscarriage or TOP) after the first antenatal record for the pregnancy. However, there may be evidence of delivery/miscarriage/TOP in one of the linked HES APC data.	• The woman must be eligible for linkage.<br>• There must be at least one day of overlap between the data coverage for each HES source and the pregstart + 294 days (42 weeks) to give a maximum potential end date.<br>• There must be a record in HES of delivery or loss within 294 days (42 weeks)
1b. The pregnancy outcome was recorded in the primary care data but has no event date recorded alongside it and is therefore not picked up by the algorithm.	There will be an outcome code with missing eventdate** within 38 weeks after the first antenatal record of the pregnancy episode (using the systemdate** as a proxy for the event date)	• There must be an antenatal code with missing eventdate** recorded with a systemdate** >= 294 days after pregnancy episode start

For peer review only - http://bmjopen.bmj.com/site/about/guidelines.xhtml

1c. The pregnancy outcome occurred before the patient was registered at their current practice or before the start of the practice up-to-standard follow-up (UTS). When the patient joined the practice, information was recorded about the pregnancy but not the outcome.	The pregnancy episode will occur before the start of the patient's current registration and/or UTS.	• Pregnancy episode end date must be < up to standard (uts) date** OR <= current registration date
Problem 2: The women was pregnant at the time of the database record***, but the pregnancy was still ongoing at the end of available follow up in the database.***		
2a. The woman moved practices before the end of her pregnancy. If a patient transfers out of a CPRD practice, then follow up is lost. OR The woman died before the end of her pregnancy.	There will be a transfer out date or death date (in either CPRD or the ONS mortality data) less than 38 weeks after the earliest antenatal record for the pregnancy episode.	• The earliest of the woman's transfer out date** or death date (in either CPRD or the ONS mortality data) minus pregnancy episode start date must be < = 294 days
2b. The last collection of data from the practice was before the pregnancy outcome.	There will be a last collection date less than 42 weeks after the start of the pregnancy episode.	• The woman's last collection date minus pregnancy episode start date must be < = 294 days
Problem 3: The patient was not pregnant at the time of the database record.		

For peer review only - http://bmjopen.bmj.com/site/about/guidelines.xhtml

3a. A historical pregnancy was recorded retrospectively in the first few months after patient joins the practice. In this scenario information about the pregnancy is recorded with the current date (by GP software default) rather than the date it occurred (different from scenario 1c). This is more likely to occur when a woman joins a practice and the GP may wish to record past pregnancy events which are relevant to her current clinical care	The pregnancy episode will occur less than one year after the women's current registration date. There will be a record of a pregnancy event which may be clinically useful for future care between the start and end of the pregnancy episode.	• Pregnancy episode start date is < 365 days after current registration date.<br>• There is a record of a pregnancy code from a list identified as likely to be recorded as useful pregnancy history information (Appendix 7).<br>• This must have an eventdate >= pregstart ** & <=pregend**
3b. The woman was not pregnant but was planning a pregnancy and discussed this with the GP, e.g. due to other medical conditions which may complicate pregnancy.	The pregnancy episode will include a pregnancy advice code, for example "67AF.00 Pregnancy advice for patients with epilepsy"	• The woman has antenatal codes identified as pregnancy advice codes (Appendix 8) with an eventdate** >= pregstart** & <= pregend **
Problem 4: The pregnancy record belongs to another pregnancy episode in the Register.		

For peer review only - http://bmjopen.bmj.com/site/about/guidelines.xhtml

4a. There was a delay in recording the outcome of a pregnancy by the practice. Thus, the outcome code has an eventdate** which is later than the true outcome date. The algorithm then calculates the LMP as being later than it was (Figure 1, steps 5 and 6). Records which occurred early in pregnancy are then left unassigned to the pregnancy episode and appear as if belonging to a previous pregnancy episode which has no outcome recorded (Figure 1, step 8).	As the pregnancy episode without outcome has been created from unassigned records at the beginning of the pregnancy it will be followed by another pregnancy episode. There is unlikely to be more than a three-month delay in outcome recording due to the mother attending the practice for postnatal checks and/or infant vaccinations. Therefore, there will be less than 12 weeks between the end of the episode with no recorded outcome and the start of the next pregnancy episode.	• The woman must have >1 episode in the pregnancy register.<br>• Episodes with recorded outcome missing were eligible if they were not the last pregnancy episode for that woman.<br>• There must be <= 84 days (12 weeks) between the pregend** of the episode without outcome and the pregstart** of the woman's next episode.

For peer review only - http://bmjopen.bmj.com/site/about/guidelines.xhtml

4b. The LMP is derived from information in the data and is estimated by the algorithm to have occurred later than reality (Figure 1, steps 5). This may lead to a short pregnancy episode and unassigned codes before the estimated start of pregnancy. These are then grouped to form a pregnancy episode with no recorded outcome (Figure 1, step 8).	The pregnancy episode without outcome will be followed by another pregnancy episode which will be less than 40 weeks long.	• The woman must have >1 episode in the pregnancy register.<br>• The episode after the episode with missing outcome must have a startsource** = to 2,4,5 or 6 (Appendix 2). The length (gestdays) of the episode must be <280 days
4c. If there are pregnancy records within 4 weeks before the estimated LMP, the identified pregnancy episode is shifted earlier in time by the algorithm (within plausible limits) to encompass those records (Figure 1, step 6). This may leave unassigned pregnancy records which occurred shortly after the new estimated delivery date which will then be grouped to form a pregnancy episode with no recorded outcome (Figure 1, step 8).	The pregnancy episode must not be the only pregnancy for this to apply. There will be another pregnancy episode which ends <8 weeks before the first antenatal record of the pregnancy episode without outcome for which the end has been adjusted by the algorithm.	• The woman must have >1 episode in the pregnancy register.<br>• The episode before the one with recorded outcome missing must have an endadj** =2 (Appendix 2)<br>• The pregend** date for the episode with missing outcome must be <= 56 days (8 weeks) after the pregend** for that previous episode.

For peer review only - http://bmjopen.bmj.com/site/about/guidelines.xhtml

4d. The GP records a code relating to the patient's pregnancy outcome history whilst the patient is pregnant. This is incorrectly identified by the algorithm as the outcome of the current pregnancy (Figure 1, step 3). If the actual outcome is <=25 weeks after for delivery or <=12 weeks after for pregnancy losses they will be grouped together as the same outcome. Subsequent antenatal records may then be grouped together to form a new pregnancy episode with no recorded outcome (Figure 1, step 8)	The pregnancy episode must not be the patient's first pregnancy. The pregnancy episode would be within 25 weeks after the previous outcome.	• The woman must have >1 episode in the pregnancy register.<br>• The pregend** date for the episode with missing outcome had to be <= 175 days (25 weeks) after the pregend** for the previous episode.
4e. The outcome of the pregnancy episode has been misclassified as an antenatal event e.g. 'Failed abortion', 'refer to TOP counselling', 'premature labour' etc.	There will be an antenatal code which should have been an outcome code within 38 weeks after the first antenatal record of the pregnancy episode with recorded outcome missing.	• There must be an antenatal record from a code list of potentially misclassified outcomes (Appendix 9) 266 days (38 weeks) of the firstantenatal** record.

*The first antenatal record is assumed to be recorded ≥4 weeks after the LMP as the woman is unlikely to know she is pregnant before then.

** Refers to a CPRD GOLD specific variable for example: pregend = the end of episode as defined by the algorithm; pregstart = the start of episode as defined by the algorithm; endadj = an indication that the end of the episode has been adjusted and how; startsource= which data were used to generate the start of the episode. These variables and others are defined in more detail in appendix 2

Table 1b: Description of potential scenarios leading to conflicting episodes and scenario criteria applied

For peer review only - http://bmjopen.bmj.com/site/about/guidelines.xhtml

Scenario	How does this appear in the data?	Criteria applied to pairs of conflicting episodes to determine if there is evidence in the data that the pair is consistent with the scenario in question.
Problem 1: Both pregnancies are true, but one is a current pregnancy and one is a historical pregnancy		
1a. The GP records a past delivery during a current pregnancy > 25weeks before the true delivery of that pregnancy. OR a past pregnancy loss > 12 weeks before the actual loss of that pregnancy	Both pregnancies will have the same outcome type. Evidence of current pregnancy codes would be expected to fall within the second pregnancy.	• The outcome combination of the two episodes must be delivery/delivery or loss/loss (see Appendix 10 for outcome classifications)<br>• The second episode had an antenatal code from a list deemed likely to only be recorded if the patient was currently pregnant (Appendix 11) OR a scan record in the HES DID data between firstantenatal* and pregend*.

For peer review only - http://bmjopen.bmj.com/site/about/guidelines.xhtml

1b. If a patient has a record relating to a previous loss recorded during a pregnancy ending in delivery or vice-versa then conflicting episodes will be created by the algorithm. The algorithm first generates episodes for consecutive deliveries; it then does the same thing for pregnancy losses. There is no step in the algorithm to check that the loss episodes do not coincide with the delivery episodes (Figure 1, steps 1-6).	The conflicting pregnancies must consist of one loss and one delivery.<br><br>Evidence of current pregnancy codes would be expected to fall within the second pregnancy.	• The outcome combination of the two episodes must be delivery/loss or loss/delivery (see Appendix 10 for outcome classifications)<br><br>• The second episode had an antenatal code from a list deemed likely to only be recorded if the patient was currently pregnant (Appendix 11) OR an antenatal scan record in the HES DID data between firstantenatal* and pregend*
Problem 2: Both pregnancies are historical		
2a. A patient joins a new practice (or has another reason for a full obstetric history to be taken) and has information on historical pregnancies recorded with the current date rather than the actual date of the event. Losses and deliveries recorded on the same date will result in conflicting episodes in the Register as different outcome types are generated separately by the algorithm (Figure 1, steps 1-5).	The conflicting pregnancies must consist of one loss and one delivery. The pregnancy end dates will be the same for both pregnancies. Both pregnancies are likely to be <1 year after the patient's current registration date. We would not expect to find codes indicating current pregnancy.	• The outcome combination of the two episodes must be a delivery and a loss.<br><br>• The pregend* dates must be the same.<br><br>• There must be no antenatal codes relating to current pregnancy (Appendix 11) or HES DID antenatal scan recorded between the firstantenatal* date and the pregend* date of either episode.

Problem 3: Both pregnancies are true and current but the gestation of the second pregnancy estimated by the algorithm is too long.		
3a. The woman has two pregnancy losses which are >8 weeks and <12 weeks apart. The second pregnancy has no information about gestation recorded so the algorithm applies a default of 12 weeks and the episodes overlap.	Both conflicting pregnancies must be losses. The maximum overlap between the two pregnancies must be 4 weeks. Evidence of current pregnancy codes could be found in either pregnancy.	• The outcome combination of the two episodes must be two losses. The pregend* for the first episode must be <= 28 days after the pregstart* of the second episode.
3b. The woman has two pregnancies close together and the second pregnancy ends in delivery. If the information on the LMP in the data of the second pregnancy is wrong, then the algorithm may generate the start too early resulting in an overlap.	The second pregnancy must be a delivery and have no information about gestation in the data. The overlap must be <15 weeks (otherwise the two outcomes would be <25 weeks apart and would have been grouped as one see Figure 1 step 3). There may be evidence of current pregnancy codes in either pregnancy	• The outcome of the second episode must be a delivery.<br>• The startsource* of the second episode must be not equal to 4 or 5 (Appendix 2)<br>• The pregstart *of the second episode must be <105 days (15 weeks) before the pregend* of the first episode.
Problem 4: The pregnancy is true and current but is split into separate episodes by the rules of the algorithm		

For peer review only

For peer review only - http://bmjopen.bmj.com/site/about/guidelines.xhtml

4a. The GP records further information about a pregnancy outcome > 25 weeks after the delivery date for pregnancies ending in delivery OR >8 weeks but <12 weeks for pregnancies ending in loss. The algorithm assumes this further information is a different pregnancy and generates a new episode, which may overlap with the "true" episode.	Both pregnancies must be of the same outcome type. Evidence of current pregnancy codes would be expected to fall within the first pregnancy.	• The outcome combination of the two episodes must be delivery/delivery or loss/loss (Appendix 12)<br>• The first episode had an antenatal code from a list deemed likely to only be recorded if the patient was currently pregnant (Appendix 11) OR a scan record in the HES DID data between firstantenatal* and pregend*.
4b. The GP records further antenatal information about a pregnancy after delivery or pregnancy loss. This will then be used to generate a new pregnancy without outcome episode by the algorithm. If the code is within 4 weeks of the end of the true pregnancy episode the two will overlap	The first pregnancy must be a pregnancy with an outcome recorded in the data. The second pregnancy must be a pregnancy without outcome which consists of one antenatal code not related to a scan.	• The first episode must have outcome= 1-10 in the register (Appendix 2) and must have endadj* =0<br>• The second episode must have no recorded outcome (outcome= 13)<br>• The second episode must have a gestdays* =28 (likely to consist of one code) and there must NOT be a scan code (Appendix 13) with an eventdate* = pregend* of the second episode.

For peer review only - http://bmjopen.bmj.com/site/about/guidelines.xhtml

4c. The patient has a follow up scan after a pregnancy loss. This is recorded in the data by the GP as an antenatal scan. The algorithm then creates a second pregnancy episode based on the antenatal scan code which becomes a pregnancy without outcome in the register.	The first pregnancy must be a pregnancy loss. The second pregnancy must be a pregnancy without outcome which consists of one antenatal code related to a scan.	• The outcome combination of the two episodes must be loss/missing.<br>• The second episode must have a gestdays* =28 (likely to consist of one code) and there must be a scan code (Appendix 13) with an eventdate* = pregend* of the second episode.

For peer review only

For peer review only - http://bmjopen.bmj.com/site/about/guidelines.xhtml

4d. The GP records information about a pregnancy but no information about the outcome. If records relating to this pregnancy are more than 6 weeks apart, they will be turned into multiple episodes. Once estimated start dates are generated for these episodes based on the data recorded (Figure 1, step 8) episodes may overlap. For example, if there is gestational information included in the second episode the start of this episode will be assigned before the start of the previous episode resulting in a nested pregnancy episode.	Both pregnancies must be pregnancies without outcome in the register. The end of the first pregnancy must be greater than six weeks before the first antenatal of the second.	• The outcome combination of the two episodes must be missing/missing.<br>• The pregend* of the first episode is > 42 days before the firstantenatal* date of the second episode.

For peer review only

4e. The first pregnancy episode ended in delivery and has been shifted backwards by the rules of the algorithm leaving unassigned late pregnancy or third trimester records. These records will then be identified by the algorithm as end of pregnancies (Figure 1, step 6) and new conflicting episodes will be created.	The first pregnancy must be a pregnancy with a delivery outcome recorded in the data. The end of the first pregnancy must have been adjusted. The second pregnancy must be a pregnancy where the outcome is based on a late pregnancy or third trimester record.	• The first episode must have a delivery outcome code and endadj* variable not = to 0<br>• The second episode must have outcome= to 11, 12 or 13.

* Refers to a CPRD GOLD specific variable for example: pregend = the end of episode as defined by the algorithm; pregstart = the start of episode as defined by the algorithm; endadj = an indication that the end of the episode has been adjusted and how; startsource= which data were used to generate the start of the episode. These variables and others are defined in more detail in appendix 2

For peer review only

Applying Criteria to identify evidence of each scenario.

Evidence in HES

For each episode it was ascertained whether the woman was eligible for linkage to other data and whether the episode occurred within the coverage period of each linked data source. For pregnancy episodes occurring within the linkage coverage period, the linked HES data was examined for evidence of pregnancy outcomes. The period for which outcomes were searched was from the episode start date to nine months after the episode end date; we excluded from this analysis pregnancies where this period was entirely outside the coverage dates for linked HES data.

ICD 10 and OPCS code lists were used to look for evidence of outcomes in the HES APC Episodes, Diagnosis and Procedures tables (Appendix 4 and 5). In the HES APC Maternity data a recording of an acceptable value in any of the variables identified as relating to delivery (Appendix 6) was flagged as evidence that a delivery had taken place. In the HES OP data an ICD 10 code list for evidence of delivery, termination or early pregnancy loss was used. Snomed codes (Appendix 14) were used to identify all foetal scan records in the HES DID data.

Pregnancy Episodes with recorded outcome missing

All episodes coded as outcome unknown ("13" in the outcome field) were extracted from the Pregnancy Register. For each episode, we extracted information on the timing of the episode in relation to the start and end of patient follow-up and the period of research standard ("up to standard", or UTS) data recording in CPRD, and we also searched for relevant codes in the patient's record, namely: early pregnancy codes which were likely to be recorded in the patient's first antenatal visits to the GP; codes which are likely to be recorded by the GP as clinically important in the patient's medical history even when the patient was not pregnant; codes which may indicate an outcome but were originally classified by the Register as antenatal; codes which are likely to be recorded by the GP as part of a consultation about the potential health impacts on a patient of becoming pregnant (code lists in Appendix 7, 9, 8).

For each scenario a set of criteria based on how these should appear in the data were established (described in detail in Table 1a). Criteria were systematically applied to the data to establish which episodes were consistent with each scenario.

Conflicting pregnancy episodes

All conflicting episodes (those with at least one day of overlap with another episode for the same woman) were ascertained using the conflict flag in the Register. Pregnancy episodes may conflict with more than one other episode. Each conflicting pair was treated separately and therefore an individual pregnancy episode could appear in the analysis multiple times. A dataset was created which contained one row per pair of conflicting pregnancy episodes.

For peer review only - http://bmjopen.bmj.com/site/about/guidelines.xhtml

Episodes were ordered by start date with episode one being the earlier start date of the two. Descriptive variables were added to the dataset from the CPRD GOLD data to indicate if the episodes were during current registration and UTS follow-up. Pregnancy episode outcomes were grouped into three categories: delivery, loss or missing, and a variable was generated to indicate the combination of outcomes in each conflicting pair (Appendix 12).

For each scenario a set of criteria based on how these should appear in the data were established (described in detail in Table 1b). Criteria were systematically applied to the data to establish which conflicting pairs were consistent with each scenario.

Patient and Public Involvement

There was no patient or public involvement in this methodological work.

Results

There were 2,438,493 women with a pregnancy episode in the February 2018 version of the Pregnancy Register of these patients 731,368 (30%) had at least one uncertain episode. Mean patient follow up time for all women was 4,720 days, this was slightly lower for women with a missing outcome record (4,349 days) (Table 2). Women with an uncertain episode were more likely to be over 30 years of age. Uncertain pregnancy episodes were also more likely to be recent (after 2000) (Table 2).

Pregnancy Episodes with recorded outcome missing

Of the 5.8 million pregnancy episodes in the Pregnancy Register there were 932,604 (16%) episodes with no recorded outcome of which over half (516,818, 55.4%) were during UTS follow up and current registration (Table 2). 826,146 (89%) had evidence consistent with at least one of the identified scenarios (Table 3). 689,737 (74%) had evidence of a scenario indicating they were true (either current or historical) pregnancies (scenarios 1a,1b, 1c, 2a, 2b or 4e). The largest proportion of pregnancy episodes occurred before the patient registered at their current practice which contributed the data to CPRD or before that practice was deemed to be contributing research standard data (415,807, 44.6% Scenario 1c). 211,070 (22.6%%) episodes had data in HES consistent with the outcome occurring in hospital and not being fed back to the GP (Scenario 1a), representing approximately 50% of episodes with recorded outcome missing which were eligible for linkage. HES APC data was the most useful linked data source for ascertaining pregnancy outcomes with a small number found in HES Outpatient (Appendix 15).

The second most common potential explanation for pregnancies without outcome was scenario 4d, where a code relating to the patient's pregnancy history may have been recorded by the GP whilst the patient was pregnant. 349,874 (37.5%) episodes without outcome were consistent with this scenario. Relatively fewer episodes were consistent with scenario 4a, 4b and 4e, none were consistent with 4c. For 242,698 (26%) episodes, follow-up ended before the predicted end of the pregnancy (Scenario 2a and 2b) for 822 episodes (<0.1%) of these episodes follow-up ended due to death. Only small proportions of episodes were consistent with other scenarios. The distribution of scenarios that occurred during the period left censored by the practice UTS date and patient current registration date was similar to that of the Pregnancy Register as a whole (Appendix 16, Table 3).

For peer review only - http://bmjopen.bmj.com/site/about/guidelines.xhtml

Table 2: Baseline characteristics of the pregnancy episodes in the February 2018 Pregnancy Register.

	**Episodes with recorded outcome missing N (%)**	**Conflicting episodes N (%)**	**All episodes in the Pregnancy Register N (%)**
Number of patients	*643,689 (26.4%)*	*210,593 (8.6%)*	*2,438,493*
Mean patient follow up time (years)	*11.92*	*12.92*	*12.93*
Mean number of pregnancy episodes per patient	*3.63*	*4.66*	*3.44*
Pregnancy end was during UTS follow up and current registration	516,818 (55.4%)	160,936 (64.1%)	1,926,077 (33.1%)
Age Group of the patient at the end of the pregnancy episode			
11-14	1,344 (0.1%)	76 (0.0%)	7,867 (0.1%)
15-19	72,543 (7.8%)	15,420 (6.1%)	551,025 (9.5%)
20-24	196,979 (21.1%)	48,273 (19.2%)	1397717 (24.0%)
25-29	254,352 (27.3%)	65,601 (26.1%)	1624350 (27.9%)
30-34	235,995 (25.3%)	69,236 (27.6%)	1339439 (23.0%)
35-39	126,369 (13.6%)	40,079 (16.0%)	685,421 (11.8%)
40-44	37,640 (4.0%)	11,355 (4.5%)	194,354 (3.3%)
45-49	7,382 (0.8%)	953 (0.4%)	24,208 (0.4%)
Year pregnancy episode ended			
pre 1950	1,417 (0.2%)	41 (0.0%)	16,695 (0.3%)
1950-1959	8,061 (0.9%)	522 (0.2%)	98,436 (1.7%)
1960-1969	19,312 (2.1%)	1,887 (0.8%)	283,757 (4.9%)
1970-1979	24,296 (2.6%)	3,882 (1.5%)	493,217 (8.5%)
1980-1989	38,768 (4.2%)	9,135 (3.6%)	803,380 (13.8%)
1990-1999	248,016 (26.6%)	54,254 (21.6%)	1,530,212 (26.3%)
2000-2009	336,523 (36.1%)	116,429 (46.4%)	1,705,380 (29.3%)
2010-2018	256,211 (27.5%)	64,843 (25.8%)	893,304 (15.3%)
**Total Pregnancies**	**932,604**	**251,026**	**5,824,381**

For peer review only

For peer review only - http://bmjopen.bmj.com/site/about/guidelines.xhtml

Table 3: Numbers of pregnancy episodes with recorded outcome missing which were consistent with applied criteria for each scenario**

Scenario	Description	N pregnancy episodes with evidence of this scenario (% of total episodes with missing outcome)	N pregnancy episodes with evidence of this scenario only (% of total episodes with missing outcome)	N pregnancy episodes with evidence of an outcome in linked HES (% of linkage eligible episodes with recorded outcome missing*)	N episodes which were during current registration and UTS follow up (% of total episodes with missing outcome)**
Denominator		932,604	932,604	424,375*	932,604
Problem 1: The women was pregnant at the time of the database record, but the outcome was not captured in CPRD primary care data.					
Scenario 1a	The pregnancy outcome occurred in hospital or elsewhere and information wasn't fed back to the practice.	211,070 (22.6%)	1,934 (0.2%)	211,070 (49.7%)	139,084 (14.9%)
Scenario 1b	The outcome of the pregnancy is recorded in the primary care data but has no event date associated with it.	1,595 (0.2%)	48 (0.0%)	523 (0.1%)	475 (0.1%)
Scenario 1c	The pregnancy occurred before the patient was registered at the practice or before UTS	415,807 (44.6%)	204,176 (21.9%)	60,423 (14.2%)	0 (0.0%)

For peer review only - http://bmjopen.bmj.com/site/about/guidelines.xhtml

Problem 2: The women was pregnant at the time of the database record, but the pregnancy was still ongoing at the end of available follow up in the database.					
Scenario 2a	The patient transferred out or died before the putative end of pregnancy	177,557 (19.0%)	40,191 (4.3%)	71,012 (16.7%)	117,571 (12.6%)
Scenario 2b	The last collection date of the practice was before the putative end of pregnancy	65,141 (7.0%)	22,039 (2.4%)	24,091 (5.7%)	58,698 (6.3%)
Problem 3: The patient was not pregnant at the time of the database record.					
Scenario 3a	Episode is derived from historical pregnancy information recorded in the first few months after the patient joined the practice	10,235 (1.1%)	588 (0.1%)	3,058 (0.7%)	3,875 (0.4%)
Scenario 3b	Patient asks for advice whilst planning a pregnancy	0 (0.0%)	0 (0.0%)	0 (0.0%)	0 (0.0%)
Problem 4: The pregnancy record belongs to another pregnancy episode in the Register.					
Scenario 4a	Delay in recording the outcome of a pregnancy, algorithm calculates LMP too late and uncovers records at the beginning of pregnancy creating this PWO.	61,662 (6.6%)	9,299 (1.0%)	23,099 (5.4%)	35,255 (3.8%)

For peer review only - http://bmjopen.bmj.com/site/about/guidelines.xhtml

Scenario 4b	The LMP is derived from the data and is wrong resulting in early codes being uncovered creating this episode	29,057 (3.1%)	4,022 (0.4%)	11,304 (2.7%)	17,110 (1.8%)
Scenario 4c	The LMP has been shifted earlier in time uncovering records at the end of the pregnancy	0 (0.0%)	0 (0.0%)	0 (0.0%)	0 (0.0%)
Scenario 4d	A code recorded relating to the patient's delivery history is incorrectly identified by the algorithm as a delivery uncovering records at the end.	349,874 (37.5%)	113,688 (12.2%)	90,274 (21.3%)	219,505 (23.5%)
Scenario 4e	The outcome of the pregnancy episode has been misclassified as antenatal	38,848 (4.2%)	8,000 (0.9%)	6,611 (1.6%)	18,222 (2.0%)
None	These pregnancy episodes did not meet the criteria for any identified scenarios.	106,458 (11.4%)	-	-	94,769 (10.2%)

* Denominator = pregnancy episodes which had at least one day overlap with the available HES follow up period and where the woman was eligible for linkage.

**A version of this table restricted to episodes which occurred during practice UTS follow-up and patient's current registration is given in the appendices (Appendix 16)

For peer review only - http://bmjopen.bmj.com/site/about/guidelines.xhtml

Conflicting Pregnancy Episodes

There were 478,341 (8.5%) pregnancy episodes with a conflict recorded in the February 2018 Pregnancy Register, amounting to 251,026 conflicting pregnancy pairs. Over half of the pairs (160,936, 64%) were during UTS follow up and current registration. There were 215,577 (88.6%) pairs which were consistent with at least one identified scenario. Of the remaining 106,458 (11.4%), less than half were during UTS follow up and current registration (table showing these pregnancies by scenario is given in Appendix 17). Across all scenarios at least 40% were during UTS follow up and current registration. Of the pregnancy pairs 215,544 (86%) had evidence of a scenario indicating that at least one episode was a true and current pregnancy (scenarios 1a, 1b, 3a, 3b, and 4a-e). Most conflicting pairs had at least one pregnancy episode ending in loss 201,783 (80.3%) (Appendix 18). Furthermore, 41% (101,760) of pairs included at least one pregnancy with no outcome recorded.

75,672 (30%) of all conflicting pairs were shown to have evidence that they were consistent with problem 1, that a patient had a record relating to the outcome of a previous pregnancy recorded during a current pregnancy. This includes scenario 1b: a record of a previous loss recorded during a pregnancy ending in delivery or vice versa, one of the most common scenarios (29% of conflicting pairs) (Table 4).

73,191 (29%) of pairs were consistent with scenario 4e: that adjusting of pregnancy dates by the algorithm had led to unassigned records. Of these, over 96% (70,472) were consistent with this scenario only, and 73% (53,464) of these pairs had a linked baby identified. 43,581 (17.4%) of episodes had evidence that they were consistent with further antenatal information having been recorded after the end of pregnancy (scenario 4b).

For approximately 16% (39,373) of conflicting pairs there was evidence to suggest that the gestation of the second pregnancy episode specified by the algorithm may have been too long leading to an overlap (scenario 3a and 3b).

Ten percent of conflicting pairs had a loss and delivery recorded on the same date and no “current pregnancy” antenatal codes suggesting they may have been recorded as part of an obstetric history (scenario 2a). Only small percentages of episodes were consistent with other scenarios. Proportional distribution of the scenarios was similar when restricted to those recorded during UTS and current registration to that of the whole Pregnancy Register.

For peer review only - http://bmjopen.bmj.com/site/about/guidelines.xhtml

Table 4: Numbers of conflicting pregnancy episodes which were consistent with applied criteria for each scenario**

Scenario	Description	N pregnancy pairs with evidence of this scenario (% of total conflicting pregnancy pairs)	N pairs with evidence of only this scenario (% of total conflicting pregnancy pairs)	N of pairs with a linked baby in the MBL (% of total conflicting pregnancy pairs)	N pairs with evidence of pregnancy in linked HES (% of pairs eligible for HES linkage*)	N pairs which were during current registration and UTS follow up MBL (% of total conflicting pregnancy pairs)
Denominator		251,026	251,026	251,026	160,461*	251,026
Problem 1: Both pregnancies are true but one is a current pregnancy and one is a historical pregnancy						
Scenario 1a	The GP records a past delivery or loss during a current pregnancy with the same outcome resulting in another episode being created	2,464 (1.0%)	413 (0.2%)	2,164 (0.9%)	2,332 (1.5%)	1,981 (0.8%)
Scenario 1b	A patient has a record relating to a loss recorded during a pregnancy ending in delivery or vice-versa. Conflicting episodes are generated by the algorithm	73,208 (29.2%)	35,026 (14.0%)	11,388 (4.5%)	19,900 (12.4%)	31,526 (12.6%)
Problem 2: Both pregnancies are historical						
Scenario 2a	A patient has information on historical pregnancies recorded with the current date rather than the actual date.	27,250 (10.9%)	0 (0.0%)	175 (0.1%)	6,835 (4.3%)	12,557 (5.0%)
Problem 3: Both pregnancies are true and current but the gestation of the second pregnancy estimated by the algorithm is too long.						
Scenario 3a	The woman has two losses which are >8weeks and <12weeks apart.	6,425 (2.6%)	12 (0.0%)	0 (0.0%)	1,336 (0.8%)	2,284 (0.9%)

For peer review only - http://bmjopen.bmj.com/site/about/guidelines.xhtml

Scenario 3b	The woman has two pregnancies close together and the second ends in delivery. If the LMP information is wrong for this pregnancy, then algorithm episodes may overlap.	32,948 (13.1%)	3,705 (1.5%)	1,564 (0.6%)	7,833 (4.9%)	13,464 (5.4%)
Problem 4: The pregnancy is real but is split into separate episodes by the rules of the algorithm						
Scenario 4a	The GP records further information about a pregnancy outcome >25 weeks later for deliveries or >8weeks <12 weeks later for losses.	2,939 (1.2%)	251 (0.1%)	2,646 (1.1%)	2,824 (1.8%)	2,347 (0.9%)
Scenario 4b	The GP records further antenatal information after the end of a pregnancy. Conflicting episodes are generated by the algorithm	43,581 (17.4%)	40,928 (16.3%)	13,531 (5.4%)	16,718 (10.4%)	27,131 (10.8%)
Scenario 4c	The patient has a follow up scan after a pregnancy loss. The scan is recorded in the data as an antenatal scan, a conflicting episode is then generated by the algorithm.	2,734 (1.1%)	0 (0.0%)	0 (0.0%)	744 (0.5%)	2,088 (0.8%)
Scenario 4d	The GP records information about a pregnancy but no outcome with >6 weeks between records. If the second episode has gestational information the start may be assigned before the start of the first episode.	14,695 (5.9%)	14,695 (5.9%)	0 (0.0%)	7,392 (4.6%)	9,911 (3.9%)
Scenario 4e	The pregnancy dates have been shifted backwards by the rules of the algorithm leaving uncovered records. Conflicting episodes are generated by the algorithm.	73,191 (29.2%)	70,472 (28.1%)	53,464 (21.3%)	42,785 (26.7%)	55,205 (22.0%)

For peer review only - http://bmjopen.bmj.com/site/about/guidelines.xhtml

For peer review only

None	These pairs of pregnancies did not meet the criteria for any identified scenarios.	35,449 (14.1%)	-	13,241 (5.3%)	14,173 (8.8%)	15,650 (6.2%)

* Denominator = pregnancy episodes which had at least one day overlap with the available HES follow up period and where the woman was eligible for linkage.

**A version of this table restricted to episodes which occurred during practice UTS follow-up and patient's current registration is given in the appendices (Appendix 17)

For peer review only

For peer review only - http://bmjopen.bmj.com/site/about/guidelines.xhtml

Discussion

This work has shown that uncertain pregnancy episodes in the CPRD pregnancy register can contain valuable information about a woman's pregnancy. A high proportion of the uncertain episodes were during research quality follow up time and therefore comprise data which would usually be included in study designs (9). We have systematically identified potential reasons for the existence of uncertain episodes within the pregnancy register to allow researchers to consider in more detail whether inclusion is appropriate for their study. This work adds further value to the CPRD Pregnancy Register which is already unique in its inclusion of all pregnancy data regardless of completion (3,4). To our knowledge no previous studies have attempted to examine uncertain pregnancies in EHR data in this way and many of the scenarios we have described will also be applicable to other EHR data sources.

We found that most episodes with a missing outcome could be explained by the outcomes not being captured in the CPRD GOLD primary care database; either the patient was not registered at the time of the pregnancy, the outcome was not recorded by the GP but could be found in linked data, or follow up ended before the outcome. These are likely to be genuine and contemporaneous pregnancies which would be missed if episodes with recorded outcome missing were excluded from the Register. In fact, most of the scenarios we identified are consistent with the episodes being true and current pregnancies. When conducting drug utilisation or vaccine uptake studies researchers may wish to include episodes where the database follow-up ended before the outcome to avoid underestimation especially for new drugs or vaccination programs. Further to our objective to provide guidance, Table 5 outlines potential considerations for researchers deciding whether to include or exclude uncertain episodes from their study.

There is evidence to suggest that historical outcomes being recorded by the GP during an ongoing pregnancy may explain a sizeable proportion of the uncertain episodes generated by the algorithm. This can lead to true pregnancies being split by the algorithm and depending on the timing this will either generate an additional episode with outcome missing or two separate episodes with outcomes (Figure 1, step 3). In either case the resulting episodes may conflict with one another. Based on our findings this appears to be something that happens fairly frequently. One concern is that these episodes are likely to appear more frequently for women with a history of complicated pregnancy outcomes. For example, previous caesarean sections may be likely to be noted by the GP during current care as would outcomes such as ectopic pregnancies. Researchers should be aware that exclusion of women who have overlapping pregnancies for this reason might therefore systematically exclude those with a history of pregnancy complications, introducing bias.

It is also possible that current pregnancies with serious complications are more likely to have an uncertain episode in the Register. For example, women with pre-eclampsia are more likely to have consultant led antenatal care carried out in hospital increasing the chances that their primary care record is incomplete and has no recorded outcome (10). This data pattern is likely to result in the pregnancy being spilt into multiple episodes without outcome (Figure 1, step 8). Dropping all uncertain episodes at the study design stage may mean that these patients are missed. Researchers who are interested in specific pregnancy complications should take this into consideration and use a tailored approach when selecting a study population.

For peer review only - http://bmjopen.bmj.com/site/about/guidelines.xhtml

Whilst some conflicting episodes may be caused by poor quality data there are many conflicting episodes for which it may be possible to clarify which time period is likely to be the true pregnancy. We found that episode conflicts were more likely to occur for pregnancies ending in loss; this is of little surprise given the wider variation around the true gestation of such pregnancies (11). There was also a large overlap between the conflicting episodes and those that were missing an outcome. Again, this is not surprising as the start and end dates for the missing outcome episodes have large margins of error, given they are often estimated based on one or two antenatal codes (Figure 1, step 8) (3). Not including uncertain episodes may lead to under ascertainment of miscarriage as an outcome. However, including them all may lead to exposure status misclassification due to mis-timed start and end dates or past pregnancy outcomes being counted.

Researchers may consider using multiple imputation to handle missing outcomes. However, there is a strong likelihood that the pattern of missing pregnancy outcomes is not missing at random and both multiple imputation and listwise deletion could result in biased results. Investigation of the linked HES data has shown that utilising this additional data alongside the Register could help users to identify many missing outcomes (12, 8, 7)). Potentially useful pregnancy outcome data was found in multiple places across the HES APC database (NHS Digital, 2021). Identifying outcomes in HES could allow users of the Register to adjust the dates of the pregnancy episodes. Whilst HES data is useful as a complementary source of information it is also an EHR database derived from data that were not collected for research purposes and there may be gaps in recording. It is however, less likely that pregnancy outcome events which happen in hospital will be recorded retrospectively and therefore dates of recorded outcomes may be considered more reliable.

Furthermore, utilising the HES DID data to access antenatal scan records offers a useful way to validate the dates of primary care pregnancy episodes as patients are unlikely to have an antenatal scan when they are not currently pregnant (13). When utilising linked data to we recommend that the study population is restricted to those patients in the Pregnancy Register who are eligible for linkage.

The main limitation of this work is that it relies on the assumption that real life scenarios will consistently result in the same data patterns. EHR data such as CPRD GOLD is not collected for the purposes of research and can be messy for a variety of reasons. The criteria we applied to identify our proposed scenarios may not have been a true fit to each pregnancy episode, this may have resulted in misclassification of the true underlying cause. Whilst we did validate a random sample of pregnancy episodes by looking at the individual Read codes recorded it was not possible to look at every episode in detail. Furthermore, some of our scenarios relied on assumptions as to why and when GPs may record clinical information relating to pregnancy. Whilst this was informed by clinician advice and clinical guidelines it may not be correct in every case. There is also the possibility that there are other scenarios which we did not identify, and special cases of scenarios that we could not test. For example, since 2007 women in the UK have been given the option of accessing midwife led care directly. Whilst information about the pregnancy should be fed to their GP this may not always be the case. A survey report by the Quality Care Commission published in 2020 estimated that in 2018 47% of women accessed antenatal care directly through a midwife (14). As yet, no routinely linked data allow for investigation of this special case of Scenario 1a.

We have described in detail reasons why uncertain pregnancy episodes may occur in the CPRD Pregnancy Register and criteria which researchers can apply to ascertain which episodes may fit each scenario. This work offers researchers the opportunity to tailor their study to accommodate these episodes where appropriate (Table 5).

For peer review only - http://bmjopen.bmj.com/site/about/guidelines.xhtml

Table 5: Issues with different approaches to dealing with uncertain episodes and recommendations

**Example uses**	**Issues with a Highly Specific Approach: Excluding all uncertain episodes**	**Issues with a Highly Sensitive Approach: Including all uncertain episodes**	**Recommended Tailored approach: Including or excluding uncertain episodes based on scenario criteria.**
Vaccine uptake study	• Underestimate of uptake during pregnancy	• Overestimate of uptake during pregnancy where historic episodes are included	• Consider utilising episodes without recorded outcome which continue after data follow-up to maximise the capture of exposure events.<br>• Consider utilising linked data to obtain additional outcomes.<br>• Exclude episodes which are likely to be derived from historical data based on our described scenarios.
Drug/Vaccine safety study	• Underestimation of pregnancies ending in loss.<br>• Underestimation of pregnancy complications	• Misclassification of exposure status<br>• Overestimation of outcomes	• Consider utilising linked data to obtain additional outcomes restricting the study population to those patients eligible for linkage.<br>• Exclude episodes which are likely to be derived from historical data based on our described scenarios.<br>• Consider merging conflicting episodes which are consistent with problem 4 and adjusting the timing accordingly (deciding which of the outcomes is likely to be the true outcome based

For peer review only - http://bmjopen.bmj.com/site/about/guidelines.xhtml

			on the scenarios we have described and then estimating a start date. This should be based on a combination of the patient's antenatal records and default duration dependent on outcome type (3)).<br>• Consider ensuring pregnancy start is at least nine months before the last data collection date to allow for attainment of outcomes
Ascertaining Pregnancy History	• Underestimation of parity.<br>• Underestimation of certain pregnancy events.<br>• Underestimation of pregnancies ending in loss.	• Overestimation of parity.	• Consider utilising linked data to obtain additional outcomes restricting the study population to those patients eligible for linkage.<br>• Exclude episodes which are likely to be derived from historical data based on our described scenarios.<br>• Consider ensuring pregnancy start is at least nine months before the last data collection date to allow for attainment of outcomes.
Excluding Pregnant Women from a study cohort	• Reduction in potential study population	• Potential misclassification of pregnancy status<br>• Potential errors in pregnancy timing	• Consider merging conflicting episodes which are consistent with problem 4 and adjusting the timing accordingly (deciding which of the outcomes is likely to be the true outcome based on the scenarios we have described and then estimating a start date. This should be based on a combination of the patient's antenatal records and a default duration dependent on outcome type (3)).Consider utilising linked data to obtain additional outcomes, restricting the study population to those patients eligible for linkage.

For peer review only - http://bmjopen.bmj.com/site/about/guidelines.xhtml

			• Exclude episodes which are likely to be derived from historical data based on our described scenarios.

33

Conclusions

This work has shown evidence that most uncertain pregnancy episodes are consistent with true and current pregnancies for which the data contains valuable information. It is important that researchers carefully consider the impact of including or excluding these episodes from their study. We have demonstrated that examining patterns of events within the primary care data or looking for further evidence in linked data can help to identify possible explanations. Here we offer users of the Pregnancy Register an insight into why these episodes exist and guidance on how to tailor their study population accordingly.

Contributors

JC, KB, ST, RW, HIM and CM contributed to the initiation, planning and design of the study. JC performed the analysis. KB, ST, RW, and CM conducted study supervision. HIM and ST provided clinical input. JC wrote the manuscript with KB, ST, RW, HIM and CM performing critical revision.

Ethics Statement

This study was approved by the Independent Scientific Advisory Committee (ISAC) for Medicines and Healthcare Products Regulatory Agency Database Research (protocol no: 17_285R2 and 19_140). The study was approved by the London School of Hygiene and Tropical Medicine Ethics Committee

Competing Interests

JC and RW are employees of CPRD. There are no other conflicts of interest to report.

Funding

This work forms part of JC's PhD which is funded by CPRD (grant number N/A). KB is funded by a Wellcome Senior Research Fellowship (220283/Z/20/Z). CM was supported by a UKRI Innovation Fellowship at Health Data Research UK London (MR/S003932/1) HIM and ST were funded by the National Institute for Health Research (NIHR) Health Protection Research Unit (HPRU) in Immunisation (IS-HPU1112-10096) at the London School of Hygiene and Tropical Medicine in partnership with Public Health England (PHE). The views expressed are those of the authors and not necessarily those of the NHS, the NIHR, the Department of Health and Social Care, or PHE.

Acknowledgements

This work uses data provided by patients and collected by the NHS as part of their care and support.

For peer review only - http://bmjopen.bmj.com/site/about/guidelines.xhtml

Data sharing statement

The data used for this study were obtained from the Clinical Practice Research Datalink (CPRD). All data are available via an application via CPRD's Research Data Governance (RDG) Process (see https://www.cprd.com/research-applications). Data acquisition is associated with a fee and subject to ethics approval. Text file versions of codelists provided in in the appendices are available from the corresponding author on request.

References

1. National Institute for Health and Care Excellence. Antenatal care for uncomplicated pregnancies. Clin Guidel [Internet]. 2008;(March):1–55. Available from: https://www.nice.org.uk/guidance/cg62/resources/antenatal-care-for-uncomplicated-pregnancies-975564597445

2. CPRD. CPRD: Clinical Practice Research Datalink [Internet]. 2021. Available from: www.cprd.com

3. Minassian C, Williams R, Meeraus WH, Smeeth L, Campbell OMR, Thomas SL. Methods to generate and validate a Pregnancy Register in the UK Clinical Practice Research Datalink primary care database. Pharmacoepidemiol Drug Saf [Internet]. 2019;(April):1–11. Available from: http://doi.wiley.com/10.1002/pds.4811

4. Margulis A V., Palmsten K, Andrade SE, Charlton RA, Hardy JR, Cooper WO, et al. Beginning and duration of pregnancy in automated health care databases: Review of estimation methods and validation results. Pharmacoepidemiology and Drug Safety. 2015.

5. Padmanabhan S. CPRD GOLD Data Specification [Internet]. 2017 [cited 2020 Jul 8]. Available from: https://cprd.com/sites/default/files/CPRD GOLD Full Data Specification v2.0_0.pdf

6. Boggon R, Gallagher A, Williams T, van Staa T. Creating a Mother Baby Link and Pregnancy Register for a UK Population. Pharmacoepidemiol Drug Saf. 2011;20(S44).

7. Padmanabhan S, Carty L, Cameron E, Ghosh RE, Williams R, Strongman H. Approach to record linkage of primary care data from Clinical Practice Research Datalink to other health-related patient data: overview and implications. Eur J Epidemiol [Internet]. 2019;34(1):91–9. Available from: https://doi.org/10.1007/s10654-018-0442-4

8. NHS Digital. Hospital Episode Statistics [Internet]. 2021. Available from: https://digital.nhs.uk/data-and-information/data-tools-and-services/data-services/hospital-episode-statistics

9. Lewis JD, Bilker WB, Weinstein RB, Strom BL. The relationship between time since registration and measured incidence rates in the General Practice Research Database. Pharmacoepidemiol Drug Saf. 2005 Jul;14(7):443–51.

10. National Institute for Health and Care Excellence. Hypertension in pregnancy: pre-eclampsia. 2020.

11. NHS. Miscarriage Overview [Internet]. 2021. Available from:

For peer review only - http://bmjopen.bmj.com/site/about/guidelines.xhtml

https://www.nhs.uk/conditions/miscarriage/

12. Harron K, Gilbert R, Cromwell D, Van Der Meulen J. Linking data for mothers and babies in de-identified electronic health data. PLoS One [Internet]. 2016;11(10):1–18. Available from: http://dx.doi.org/10.1371/journal.pone.0164667

13.NHS Digital. Diagnostic Imaging Dataset [Internet]. 2021. Available from: https://did.hscic.gov.uk/

14. Care Quality Commission. 2018 survey of women's experiences of maternity care [Internet]. 2020. Available from: https://www.cqc.org.uk/sites/default/files/20200128_mat19_statisticalrelease.pdf

For peer review only

Figure 1: Pregnancy Register Algorithm Steps used to create the CPRD Pregnancy Register

For peer review only - http://bmjopen.bmj.com/site/about/guidelines.xhtml

For peer review only

For peer review only - http://bmjopen.bmj.com/site/about/guidelines.xhtml

For peer review only

1. Identify all pregnancy outcome records : All records relating to pregnancy outcomes of any type (live births, stillbirths and early pregnancy losses) are extracted. Records relating to deliveries are considered separately to those relating to early pregnancy losses.

2. Date the first pregnancy outcome: The date of each woman's first pregnancy outcome is estimated using the records identified in step 1 and additional data from linked babies' records (for live births, when available) .

3. Group together records relating to the first pregnancy outcome and characterise the pregnancy outcome: Additional pregnancy outcome records relating to each woman's first outcome which are <= 25 weeks apart for deliveries and <= 8weeks apart for pregnancy losses, are assigned to that outcome. Characteristics of the delivery (pre- or post-term, stillbirth, multiple birth) or the type of early pregnancy loss (miscarriage, induced abortion, ectopic, molar pregnancy or blighted ovum) are determined from these assigned records.

4. Date and characterise each successive pregnancy outcome *:* Steps 2 & 3 are repeated to identify, date and characterise successive pregnancy outcomes sequentially for each woman.

5. Estimate the start of each pregnancy episode *:* Records relating to the timing of the start of pregnancy (first day of last menstrual period) are used to estimate pregnancy start dates. These include information such as records of gestational age, estimated date of delivery and estimated date of conception. In the absence of such data, pregnancy start dates are imputed according to the type of pregnancy outcome (40 weeks when the pregnancy is not flagged as preterm, post-term or multiple , 36 weeks for pre-term pregnancies , 37 weeks for multiple pregnancies, 41 weeks for post term pregnancies).

6. Adjust the start and end dates of a pregnancy *:* Adjustments to pregnancy start and end dates are made either when antenatal records are identified in the 4 weeks before the estimated pregnancy start date (indicating that the initial estimated start date was too late), or when the estimated pregnancy duration exceeds the maximum duration for that type of pregnancy outcome.

7. Assign antenatal records to each pregnancy episode *:* Antenatal records occurring between the start and end date of an identified pregnancy episode are assigned to the pregnancy.

8. Identify additional pregnancies with no recorded outcome *:* All remaining unassigned antenatal records are extracted and categorised into distinct pregnancy episodes. Successive records which are <= 6 weeks apart are grouped together to create a pregnancy. The date of the latest antenatal record in the episode is used as a proxy for the pregnancy end date. The pregnancy start date is estimated using information on gestational age (when available) or by subtracting 4 weeks from the earliest antenatal record in the episode.

Appendix

Appendix 1: Key CPRD GOLD variables

Column name	*Field name*	*Description*
Last Collection Date	lcd	Date of the last collection for the practice
Up to Standard Date	uts	Date at which the practice data is deemed to be of research quality. Derived using a CPRD algorithm that primarily looks at practice death recording and gaps in the data
First Registration Date	frd	Date the patient first registered with the practice.
Current Registration Date	crd	Date the patient's current period of registration with the practice began.
Transfer Out Date	tod	Date the patient transferred out of the practice, if relevant. Empty for patients who have not transferred out
Death Date	deathdate	Patient's date of death – derived using a CPRD algorithm
Acceptable Patient Flag	accept	Flag to indicate whether the patient has met certain quality standards: 1 = acceptable, 0 = unacceptable
Event Date	eventdate	Date associated with the event, as entered by the GP

For peer review only

System Date	sysdate	The date on which information was entered on to the GP software system (generated automatically)

For peer review only

Appendix 2: CPRD Pregnancy Register Variables

Field name	*Description*
Patid	Encrypted unique patient identifier
Pregid	Unique identifier of the pregnancy episode
Mblbabies	Number of babies the pregnancy is linked to in the MBL
babypatid[1]	Encrypted unique patient identifier (linked baby)
babymob	Baby's month of birth as recorded in the baby's medical record
babyyob	Baby's year of birth as recorded in the baby's medical record
totalpregs	Total number of identified pregnancy episodes (per woman)
pregnumber	Pregnancy episode number (per woman)
pregstart	Estimated start date of pregnancy
firstantenatal	Date of earliest antenatal record within the pregnancy

For peer review only

Appendix 3 Example of how a pregnancy may appear in the Register vs GOLD data vs reality

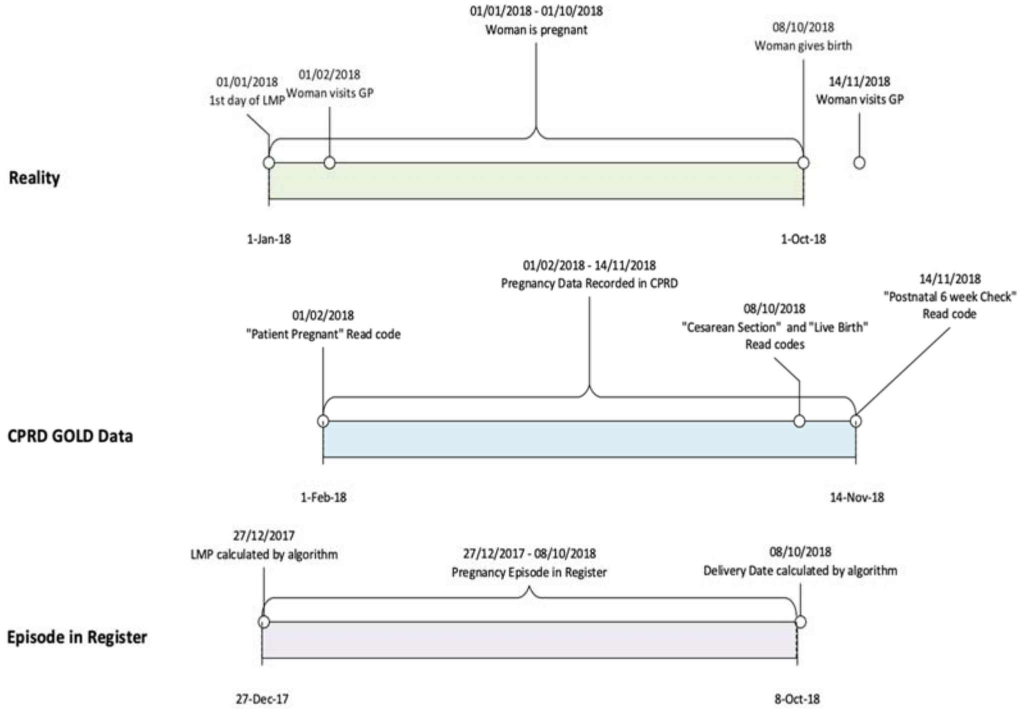

Appendix 4: ICD codes indicating end of pregnancy

O00	Ectopic pregnancy	
O00.0	Abdominal pregnancy	
O00.1	Tubal pregnancy	
O00.2	Ovarian pregnancy	
O00.8	Other ectopic pregnancy	
O00.9	Ectopic pregnancy, unspecified	
O01	Hydatidiform mole	
O01.0	Classical hydatidiform mole	
O01.1	Incomplete and partial hydatidiform mole	
O01.9	Hydatidiform mole, unspecified	
O02	Other abnormal products of conception	
O02.0	Blighted ovum and nonhydatidiform mole	
O02.1	Missed abortion	
O02.8	Other specified abnormal products of conception	
O02.9	Abnormal product of conception, unspecified	
O03	Spontaneous abortion	
O03.0	Spontaneous abortion	Incomplete, complicated by genital tract and pelvic infection
O03.1	Spontaneous abortion	Incomplete, complicated by delayed or excessive haemorrhage
O03.2	Spontaneous abortion	Incomplete, complicated by embolism
O03.3	Spontaneous abortion	Incomplete, with other and unspecified complications
O03.4	Spontaneous abortion	Incomplete, without complication
O03.5	Spontaneous abortion	Complete or unspecified, complicated by genital tract and pelvic infection
O03.6	Spontaneous abortion	Complete or unspecified, complicated by delayed or excessive haemorrhage
O03.7	Spontaneous abortion	Complete or unspecified, complicated by embolism
O03.8	Spontaneous abortion	Complete or unspecified, with other and unspecified complications
O03.9	Spontaneous abortion	Complete or unspecified, without complication

For peer review only

O04	Medical abortion	
O04.0	Medical abortion	Incomplete, complicated by genital tract and pelvic infection
O04.1	Medical abortion	Incomplete, complicated by delayed or excessive haemorrhage
O04.2	Medical abortion	Incomplete, complicated by embolism
O04.3	Medical abortion	Incomplete, with other and unspecified complications
O04.4	Medical abortion	Incomplete, without complication
O04.5	Medical abortion	Complete or unspecified, complicated by genital tract and pelvic infection
O04.6	Medical abortion	Complete or unspecified, complicated by delayed or excessive haemorrhage
O04.7	Medical abortion	Complete or unspecified, complicated by embolism
O04.8	Medical abortion	Complete or unspecified, with other and unspecified complications
O04.9	Medical abortion	Complete or unspecified, without complication
O05	Other abortion	
O05.0	Other abortion	Incomplete, complicated by genital tract and pelvic infection
O05.1	Other abortion	Incomplete, complicated by delayed or excessive haemorrhage
O05.2	Other abortion	Incomplete, complicated by embolism
O05.3	Other abortion	Incomplete, with other and unspecified complications
O05.4	Other abortion	Incomplete, without complication
O05.5	Other abortion	Complete or unspecified, complicated by genital tract and pelvic infection
O05.6	Other abortion	Complete or unspecified, complicated by delayed or excessive haemorrhage
O05.7	Other abortion	Complete or unspecified, complicated by embolism
O05.8	Other abortion	Complete or unspecified, with other and unspecified complications
O05.9	Other abortion	Complete or unspecified, without complication
O06	Unspecified abortion	
O06.0	Unspecified abortion	Incomplete, complicated by genital tract and pelvic infection
O06.1	Unspecified abortion	Incomplete, complicated by delayed or excessive haemorrhage
O06.2	Unspecified abortion	Incomplete, complicated by embolism
O06.3	Unspecified abortion	Incomplete, with other and unspecified complications

For peer review only

O06.4	Unspecified abortion	Incomplete, without complication
O06.5	Unspecified abortion	Complete or unspecified, complicated by genital tract and pelvic infection
O06.6	Unspecified abortion	Complete or unspecified, complicated by delayed or excessive haemorrhage
O06.7	Unspecified abortion	Complete or unspecified, complicated by embolism
O06.8	Unspecified abortion	Complete or unspecified, with other and unspecified complications
O06.9	Unspecified abortion	Complete or unspecified, without complication
O07	Failed attempted abortion	
O07.0	Failed medical abortion, complicated by genital tract and pelvic infection	
O07.1	Failed medical abortion, complicated by delayed or excessive haemorrhage	
O07.2	Failed medical abortion, complicated by embolism	
O07.3	Failed medical abortion, with other and unspecified complications	
O07.4	Failed medical abortion, without complication	
O07.5	Other and unspecified failed attempted abortion, complicated by genital tract and pelvic infection	
O07.6	Other and unspecified failed attempted abortion, complicated by delayed or excessive haemorrhage	
O07.7	Other and unspecified failed attempted abortion, complicated by embolism	
O07.8	Other and unspecified failed attempted abortion, with other and unspecified complications	
O07.9	Other and unspecified failed attempted abortion, without complication	
O08	Complications following abortion and ectopic and molar pregnancy	
O08.0	Genital tract and pelvic infection following abortion and ectopic and molar pregnancy	
O08.1	Delayed or excessive haemorrhage following abortion and ectopic and molar pregnancy	
O08.2	Embolism following abortion and ectopic and molar pregnancy	
O08.3	Shock following abortion and ectopic and molar pregnancy	
O08.4	Renal failure following abortion and ectopic and molar pregnancy	
O08.5	Metabolic disorders following abortion and ectopic and molar pregnancy	
O08.6	Damage to pelvic organs and tissues following abortion and ectopic and molar pregnancy	
O08.7	Other venous complications following abortion and ectopic and molar pregnancy	
O08.8	Other complications following abortion and ectopic and molar pregnancy	
O08.9	Complication following abortion and ectopic and molar pregnancy, unspecified	

For peer review only

O60.1	Preterm spontaneous labour with preterm delivery
O60.2	Preterm spontaneous labour with term delivery
O62.3	Precipitate labour
O68	Labour and delivery complicated by fetal stress [distress]
O68.0	Labour and delivery complicated by fetal heart rate anomaly
O68.1	Labour and delivery complicated by meconium in amniotic fluid
O68.2	Labour and delivery complicated by fetal heart rate anomaly with meconium in amniotic fluid
O68.3	Labour and delivery complicated by biochemical evidence of fetal stress
O68.8	Labour and delivery complicated by other evidence of fetal stress
O68.9	Labour and delivery complicated by fetal stress, unspecified
O69	Labour and delivery complicated by umbilical cord complications
O69.0	Labour and delivery complicated by prolapse of cord
O69.1	Labour and delivery complicated by cord around neck, with compression
O69.2	Labour and delivery complicated by other cord entanglement, with compression
O69.3	Labour and delivery complicated by short cord
O69.4	Labour and delivery complicated by vasa praevia
O69.5	Labour and delivery complicated by vascular lesion of cord
O69.8	Labour and delivery complicated by other cord complications
O69.9	Labour and delivery complicated by cord complication, unspecified

For peer review only

O70	Perineal laceration during delivery
O70.0	First degree perineal laceration during delivery
O70.1	Second degree perineal laceration during delivery
O70.2	Third degree perineal laceration during delivery
O70.3	Fourth degree perineal laceration during delivery
O70.9	Perineal laceration during delivery, unspecified
O74	Complications of anaesthesia during labour and delivery
O74.0	Aspiration pneumonitis due to anaesthesia during labour and delivery
O74.1	Other pulmonary complications of anaesthesia during labour and delivery
O74.2	Cardiac complications of anaesthesia during labour and delivery
O74.3	Central nervous system complications of anaesthesia during labour and delivery
O74.4	Toxic reaction to local anaesthesia during labour and delivery
O74.5	Spinal and epidural anaesthesia-induced headache during labour and delivery
O74.6	Other complications of spinal and epidural anaesthesia during labour and delivery
O74.7	Failed or difficult intubation during labour and delivery
O74.8	Other complications of anaesthesia during labour and delivery
O74.9	Complication of anaesthesia during labour and delivery, unspecified
O75	Other complications of labour and delivery, not elsewhere classified
O75.0	Maternal distress during labour and delivery

For peer review only

O75.1	Shock during or following labour and delivery
O75.5	Delayed delivery after artificial rupture of membranes
O75.6	Delayed delivery after spontaneous or unspecified rupture of membranes
O75.7	Vaginal delivery following previous caesarean section
O75.8	Other specified complications of labour and delivery
O75.9	Complication of labour and delivery, unspecified
O80	Single spontaneous delivery
O80.0	Spontaneous vertex delivery
O80.1	Spontaneous breech delivery
O80.8	Other single spontaneous delivery
O80.9	Single spontaneous delivery, unspecified
O81	Single delivery by forceps and vacuum extractor
O81.0	Low forceps delivery
O81.1	Mid-cavity forceps delivery
O81.3	Other and unspecified forceps delivery
O81.4	Vacuum extractor delivery
O81.5	Delivery by combination of forceps and vacuum extractor
O82	Single delivery by caesarean section
O82.0	Delivery by elective caesarean section

For peer review only

O82.1	Delivery by emergency caesarean section
O82.2	Delivery by caesarean hysterectomy
O82.8	Other single delivery by caesarean section
O82.9	Delivery by caesarean section, unspecified
O83	Other assisted single delivery
O83.0	Breech extraction
O83.1	Other assisted breech delivery
O83.2	Other manipulation-assisted delivery
O83.4	Destructive operation for delivery
O83.8	Other specified assisted single delivery
O83.9	Assisted single delivery, unspecified
O84	Multiple delivery
O84.0	Multiple delivery, all spontaneous
O84.1	Multiple delivery, all by forceps and vacuum extractor
O84.2	Multiple delivery, all by caesarean section
O84.8	Other multiple delivery
O84.9	Multiple delivery, unspecified
P03	Fetus and newborn affected by other complications of labour and delivery
P03.0	Fetus and newborn affected by breech delivery and extraction

For peer review only

P03.1	Fetus and newborn affected by other malpresentation, malposition and disproportion during labour and delivery
P03.2	Fetus and newborn affected by forceps delivery
P03.3	Fetus and newborn affected by delivery by vacuum extractor [ventouse]
P03.4	Fetus and newborn affected by caesarean delivery
P03.5	Fetus and newborn affected by precipitate delivery
P03.8	Fetus and newborn affected by other specified complications of labour and delivery
P03.9	Fetus and newborn affected by complication of labour and delivery, unspecified
P04.0	Fetus and newborn affected by maternal anaesthesia and analgesia in pregnancy, labour and delivery
P20.1	Intrauterine hypoxia first noted during labour and delivery
P61.2	Anaemia of prematurity
Z37	Outcome of delivery
Z37.0	Single live birth
Z37.1	Single stillbirth
Z37.2	Twins, both liveborn
Z37.3	Twins, one liveborn and one stillborn
Z37.4	Twins, both stillborn
Z37.5	Other multiple births, all liveborn
Z37.6	Other multiple births, some liveborn
Z37.7	Other multiple births, all stillborn

For peer review only

Z38	Liveborn infants according to place of birth
Z38.0	Singleton, born in hospital
Z38.1	Singleton, born outside hospital
Z38.2	Singleton, unspecified as to place of birth
Z38.3	Twin, born in hospital
Z38.4	Twin, born outside hospital
Z38.5	Twin, unspecified as to place of birth
Z38.6	Other multiple, born in hospital
Z38.7	Other multiple, born outside hospital
Z38.8	Other multiple, unspecified as to place of birth
Z39.0	Care and examination immediately after delivery

For peer review only

Appendix 5: OPCS codes indicating end of pregnancy

OPCS		
P141	INCISION OF INTROITUS OF VAGINA	POSTERIOR EPISIOTOMY AND DIVISION OF LEVATOR ANI MUSCLE
P142	INCISION OF INTROITUS OF VAGINA	POSTERIOR EPISIOTOMY NEC
P143	INCISION OF INTROITUS OF VAGINA	ANTERIOR EPISIOTOMY
Q101	CURETTAGE OF UTERUS	DILATION OF CERVIX UTERI AND CURETTAGE OF PRODUCTS OF CONCEP
Q102	CURETTAGE OF UTERUS	CURETTAGE OF PRODUCTS OF CONCEPTION FROM UTERUS NEC
Q111	OTHER EVACUATION OF CONTENTS OF UTERUS	VACUUM ASPIRATION OF PRODUCTS OF CONCEPTION FROM UTERUS NEC
Q112	OTHER EVACUATION OF CONTENTS OF UTERUS	DILATION OF CERVIX UTERI AND EVACUATION OF PRODUCTS OF CONCE
Q113	OTHER EVACUATION OF CONTENTS OF UTERUS	EVACUATION OF PRODUCTS OF CONCEPTION FROM UTERUS NEC
Q115	OTHER EVACUATION OF CONTENTS OF UTERUS	VACUUM ASPIRATION/PRODUCTS OF CONCEPTION/UTERUS USING RIGID
Q116	OTHER EVACUATION OF CONTENTS OF UTERUS	VACUUM ASPIRATION/PRODUCTS OF CONCEPTION/UTERUS USING FLEXI
Q141	INTRODUCTION OF ABORTIFACIENT INTO UTERINE CAVITY	INTRA-AMNIOTIC INJECTION OF PROSTAGLANDIN
Q142	INTRODUCTION OF ABORTIFACIENT INTO UTERINE CAVITY	INTRA-AMNIOTIC INJECTION OF ABORTIFACIENT NEC
Q143	INTRODUCTION OF ABORTIFACIENT INTO UTERINE CAVITY	EXTRA-AMNIOTIC INJECTION OF PROSTAGLANDIN
Q144	INTRODUCTION OF ABORTIFACIENT INTO UTERINE CAVITY	EXTRA-AMNIOTIC INJECTION OF ABORTIFACIENT NEC
Q145	INTRODUCTION OF ABORTIFACIENT INTO UTERINE CAVITY	INSERTION OF PROSTAGLANDIN PESSARY

For peer review only

Q146	INTRODUCTION OF ABORTIFACIENT INTO UTERINE CAVITY	INSERTION OF ABORTIFACIENT PESSARY NEC
Q148	INTRODUCTION OF ABORTIFACIENT INTO UTERINE CAVITY	OTHER SPECIFIED
Q149	INTRODUCTION OF ABORTIFACIENT INTO UTERINE CAVITY	UNSPECIFIED
R031	SELECTIVE DESTRUCTION OF FETUS	EARLY SELECTIVE FETICIDE
R032	SELECTIVE DESTRUCTION OF FETUS	LATE SELECTIVE FETICIDE
R038	SELECTIVE DESTRUCTION OF FETUS	OTHER SPECIFIED
R039	SELECTIVE DESTRUCTION OF FETUS	UNSPECIFIED
R141	SURGICAL INDUCTION OF LABOUR	FOREWATER RUPTURE OF AMNIOTIC MEMBRANE
R142	SURGICAL INDUCTION OF LABOUR	HINDWATER RUPTURE OF AMNIOTIC MEMBRANE
R148	SURGICAL INDUCTION OF LABOUR	OTHER SPECIFIED
R149	SURGICAL INDUCTION OF LABOUR	UNSPECIFIED
R151	OTHER INDUCTION OF LABOUR	MEDICAL INDUCTION OF LABOUR
R158	OTHER INDUCTION OF LABOUR	OTHER SPECIFIED
R159	OTHER INDUCTION OF LABOUR	UNSPECIFIED
R171	ELECTIVE CAESAREAN DELIVERY	ELECTIVE UPPER UTERINE SEGMENT CAESAREAN DELIVERY
R172	ELECTIVE CAESAREAN DELIVERY	ELECTIVE LOWER UTERINE SEGMENT CAESAREAN DELIVERY
R178	ELECTIVE CAESAREAN DELIVERY	OTHER SPECIFIED
R179	ELECTIVE CAESAREAN DELIVERY	UNSPECIFIED
R181	OTHER CAESAREAN DELIVERY	UPPER UTERINE SEGMENT CAESAREAN DELIVERY NEC
R182	OTHER CAESAREAN DELIVERY	LOWER UTERINE SEGMENT CAESAREAN DELIVERY NEC
R188	OTHER CAESAREAN DELIVERY	OTHER SPECIFIED
R189	OTHER CAESAREAN DELIVERY	UNSPECIFIED

For peer review only

R191	BREECH EXTRACTION DELIVERY	BREECH EXTRACTION DELIVERY WITH VERSION
R198	BREECH EXTRACTION DELIVERY	OTHER SPECIFIED
R199	BREECH EXTRACTION DELIVERY	UNSPECIFIED
R201	OTHER BREECH DELIVERY	SPONTANEOUS BREECH DELIVERY
R202	OTHER BREECH DELIVERY	ASSISTED BREECH DELIVERY
R208	OTHER BREECH DELIVERY	OTHER SPECIFIED
R209	OTHER BREECH DELIVERY	UNSPECIFIED
R211	FORCEPS CEPHALIC DELIVERY	HIGH FORCEPS CEPHALIC DELIVERY WITH ROTATION
R212	FORCEPS CEPHALIC DELIVERY	HIGH FORCEPS CEPHALIC DELIVERY NEC
R213	FORCEPS CEPHALIC DELIVERY	MID FORCEPS CEPHALIC DELIVERY WITH ROTATION
R214	FORCEPS CEPHALIC DELIVERY	MID FORCEPS CEPHALIC DELIVERY NEC
R215	FORCEPS CEPHALIC DELIVERY	LOW FORCEPS CEPHALIC DELIVERY
R218	FORCEPS CEPHALIC DELIVERY	OTHER SPECIFIED
R219	FORCEPS CEPHALIC DELIVERY	UNSPECIFIED
R221	VACUUM DELIVERY	HIGH VACUUM DELIVERY
R222	VACUUM DELIVERY	LOW VACUUM DELIVERY
R223	VACUUM DELIVERY	VACUUM DELIVERY BEFORE FULL DILATION OF CERVIX
R228	VACUUM DELIVERY	OTHER SPECIFIED
R229	VACUUM DELIVERY	UNSPECIFIED
R231	CEPHALIC VAGINAL DELIVERY WITH ABNORMAL PRESENTATION OF	MANIPULATIVE CEPHALIC VAGINAL DELIVERY WITH ABNORMAL PRESENT
R232	CEPHALIC VAGINAL DELIVERY WITH ABNORMAL PRESENTATION OF	NON-MANIPULATIVE CEPHALIC VAGINAL DELIVERY WITH ABNORMAL PRE
R238	CEPHALIC VAGINAL DELIVERY WITH ABNORMAL PRESENTATION OF	OTHER SPECIFIED

For peer review only

R239	CEPHALIC VAGINAL DELIVERY WITH ABNORMAL PRESENTATION OF	UNSPECIFIED
R249	NORMAL DELIVERY	ALL
R251	OTHER METHODS OF DELIVERY	CAESAREAN HYSTERECTOMY
R252	OTHER METHODS OF DELIVERY	DESTRUCTIVE OPERATION TO FACILITATE DELIVERY
R258	OTHER METHODS OF DELIVERY	OTHER SPECIFIED
R259	OTHER METHODS OF DELIVERY	UNSPECIFIED
R271	OTHER OPERATIONS TO FACILITATE DELIVERY	EPISIOTOMY TO FACILITATE DELIVERY
R278	OTHER OPERATIONS TO FACILITATE DELIVERY	OTHER SPECIFIED
R279	OTHER OPERATIONS TO FACILITATE DELIVERY	UNSPECIFIED
R281	INSTRUMENTAL REMOVAL/PRODUCTS/CONCEPTION FROM DEL.UTERU	CURETTAGE OF DELIVERED UTERUS
R288	INSTRUMENTAL REMOVAL/PRODUCTS/CONCEPTION FROM DEL.UTERU	OTHER SPECIFIED
R289	INSTRUMENTAL REMOVAL/PRODUCTS/CONCEPTION FROM DEL.UTERU	UNSPECIFIED
R291	MANUAL REMOVAL/PRODUCTS/CONCEPTION FROM DELIVERED UTERU	MANUAL REMOVAL OF PLACENTA FROM DELIVERED UTERUS
R298	MANUAL REMOVAL/PRODUCTS/CONCEPTION FROM DELIVERED UTERU	OTHER SPECIFIED
R299	MANUAL REMOVAL/PRODUCTS/CONCEPTION FROM DELIVERED UTERU	UNSPECIFIED

For peer review only

R301	OTHER OPERATIONS ON DELIVERED UTERUS	REPOSITIONING OF INVERTED DELIVERED UTERUS
R302	OTHER OPERATIONS ON DELIVERED UTERUS	EXPRESSION OF PLACENTA
R303	OTHER OPERATIONS ON DELIVERED UTERUS	INSTRUMENTAL EXPLORATION OF DELIVERED UTERUS NEC
R304	OTHER OPERATIONS ON DELIVERED UTERUS	MANUAL EXPLORATION OF DELIVERED UTERUS NEC
R308	OTHER OPERATIONS ON DELIVERED UTERUS	OTHER SPECIFIED
R309	OTHER OPERATIONS ON DELIVERED UTERUS	UNSPECIFIED
R321	REPAIR OF OBSTETRIC LACERATION	REPAIR OF OBSTETRIC LACERATION OF UTERUS OR CERVIX UTERI
R322	REPAIR OF OBSTETRIC LACERATION	REPAIR OF OBSTETRIC LACERATION OF PERINEUM AND SPHINCTER
R323	REPAIR OF OBSTETRIC LACERATION	REPAIR OF OBSTETRIC LACERATION OF VAGINA AND FLOOR OF PELVIS
R324	REPAIR OF OBSTETRIC LACERATION	REPAIR OF MINOR OBSTETRIC LACERATION
R325	REPAIR OF OBSTETRIC LACERATION	REPAIR OBSTETRIC LACERATION PERINEUM SPHINCTER MUCOSA ANUS
R328	REPAIR OF OBSTETRIC LACERATION	OTHER SPECIFIED
R329	REPAIR OF OBSTETRIC LACERATION	UNSPECIFIED

For peer review only

Appendix 6: HES Maternity Values to indicate delivery

Variable	*Definition*	*Acceptable values*
numbaby	Number of babies delivered	1-4
delmeth	Method used to deliver a baby that is a registrable birth	0-9
delplac	Actual type of delivery place	0-8
delprean	Anaesthetic or analgesic administered before and during labour and delivery	1-7
delposan	Anaesthetic or analgesic administered after delivery	1-7
neodur	Baby's age in days	>=1
neocare	Neonatal level of care	0-3
postdur	Postnatal days of stay	>=1

For peer review only

Appendix 7: Pregnancy Read codes identified as likely to be recorded as useful pregnancy history

medcode	read_oxmis_code	read_oxmis_term
164	635..13	Premature baby
165	L04..11	Miscarriage
255	L05..12	Termination of pregnancy
364	7F13111	Lower uterine segment caesarean section (LSCS) NEC
618	L398400	Delivery by emergency caesarean section
683	Q420.00	Haemolytic disease due to rhesus isoimmunisation
720	L398.00	Caesarean delivery
740	7F12.00	Elective caesarean delivery
863	L398200	Caesarean section - pregnancy at term
974	Q4z..15	Stillbirth NEC
1413	L264.00	Intrauterine death
1492	L36..00	Postpartum haemorrhage (PPH)
1744	L03..00	Ectopic pregnancy
2240	Q4z..12	Neonatal death
2638	L1...00	Pregnancy complications
2639	E204.11	Postnatal depression
2664	L180900	Gestational diabetes mellitus
2787	L11..11	Antepartum haemorrhage
2923	62T1.00	Puerperal depression
2924	7E06600	Hysterotomy and termination of pregnancy
3029	L166500	Infections of kidney in pregnancy
3085	7F12z00	Elective caesarean delivery NOS
3327	L13..11	Hyperemesis gravidarum
3874	L031200	Tubal abortion
4367	L362.00	Secondary and delayed postpartum haemorrhage
4530	L00..00	Hydatidiform mole
4607	L414.00	Postnatal deep vein thrombosis

For peer review only

4638	7F13.00	Other caesarean delivery
4786	L213200	Multiple delivery, all by caesarean section
4979	Eu53012	[X]Postpartum depression NOS
5113	L39y411	Postnatal vaginal discomfort
5464	L11y100	Other antepartum haemorrhage - delivered
7174	L43..00	Obstetric pulmonary embolism
7670	L398z00	Caesarean delivery NOS
7916	Z254500	Delivered by caesarean section - pregnancy at term
8147	L264.11	Fetal death in utero
8295	Q48D100	[X]Macerated stillbirth
8446	L180811	Gestational diabetes mellitus
8776	Q48D.00	[X] Stillbirth
8906	ZV27.12	[V]Stillbirth
9067	L125.00	Severe pre-eclampsia
9668	7F12100	Elective lower uterine segment caesarean delivery
9800	L398300	Delivery by elective caesarean section
10049	7F12111	Elective lower uterine segment caesarean section (LSCS)
10278	L180800	Diabetes mellitus arising in pregnancy
11359	L180.00	Diabetes mellitus during pregnancy/childbirth/puerperium
11947	L181500	Postpartum thyroiditis
11986	7E13300	Excision of ruptured ectopic tubal pregnancy
12090	L126.00	Eclampsia
12118	7F13300	Emergency caesarean section
12320	L09..11	Complications following abortion/ectopic/molar pregnancies
13307	Eu53011	[X]Postnatal depression NOS
13584	3885	Edinburgh postnatal depression scale
15061	L13..12	Hyperemesis of pregnancy
15514	7F13000	Upper uterine segment caesarean delivery NEC
15533	L451400	Obstetric breast abscess with postnatal complication

For peer review only

16250	L414.12	Phlegmasia alba dolens - obstetric
16281	L45z400	Obstetric breast infection NOS with postnatal complication
16321	L360.00	Third-stage postpartum haemorrhage
17614	Eu53111	[X]Puerperal psychosis NOS
17744	7F13100	Lower uterine segment caesarean delivery NEC
18258	L167.00	Liver disorder in pregnancy
18369	ZV27100	[V]Single stillbirth
18702	6G00.00	Postnatal depression counselling
18770	Q20yz13	Renal injury due to birth trauma
18830	L414.11	DVT - deep venous thrombosis, postnatal
20152	L090y00	Sepsis NOS following abortion/ectopic/molar pregnancy
20165	L363.00	Postpartum coagulation defects
20307	L091.00	Delayed/excessive haemorrhage following abortive pregnancy
20573	Q48D000	[X]Fresh stillbirth
22775	L11y.00	Other antepartum haemorrhage
23015	6334	Twins - 1 still + 1 live born
23588	L414200	Postnatal deep vein thrombosis with postnatal complication
23642	Eu53z00	[X]Puerperal mental disorder, unspecified
24089	L356z00	Obstetric damage to pelvic joints and ligaments NOS
24927	Eu53.00	[X]Mental and behav disorders assoc with the puerperium NEC
24951	L18C.00	Endocrine nutrition+metab dis complic pregn,childbirth+puerp
25028	L09z.00	Complication NOS following abortion/ectopic/molar pregnancy
25415	Q411.00	Perinatal intraventricular haemorrhage
28364	Q420.12	Rhesus isoimmunisation of the newborn
28861	L398500	Delivery by caesarean hysterectomy
29155	7F1A000	Caesarean hysterectomy

For peer review only

31203	6332	Single stillbirth
31857	Q204.00	Spine or spinal cord injury due to birth trauma
32950	L03y100	Cornual pregnancy
33477	L398100	Caesarean delivery - delivered
33724	L03z.00	Ectopic pregnancy NOS
34136	L120z00	Benign essential hypertension in preg/childb/puerp NOS
34173	L12B.00	Proteinuric hypertension of pregnancy
34299	L240.00	Congenital abnormality of uterus in preg/childbirth/puerp
34502	6335	Twins - both still born
34639	L180100	Diabetes mellitus during pregnancy - baby delivered
34868	L4...00	Complications of the puerperium
35190	7F13z00	Other caesarean delivery NOS
35309	6755	Post miscarriage counselling
36421	L167z00	Liver disorder in pregnancy NOS
37280	L36z.00	Postpartum haemorrhage NOS
39117	L126500	Eclampsia in pregnancy
40224	Eu53000	[X]Mild mental/behav disorder assoc with the puerperium NEC
40500	Eu53100	[X]Severe mental and behav disorder assoc wth puerperium NEC
40730	L125z00	Severe pre-eclampsia NOS
42088	L125100	Severe pre-eclampsia - delivered
42598	L175.00	Maternal rubella in pregnancy, childbirth and the puerperium
44494	L441z00	Caesarean wound disruption NOS
45806	L070x00	Unspecified abortion with complication NOS
46756	L184.00	Mental disorders in pregnancy, childbirth and the puerperium
47227	ZV27300	[V]Twins, one live born and one stillborn
47542	L362200	Secondary postpartum haemorrhage with postnatal problem

For peer review only

47546	7F12y00	Other specified elective caesarean delivery
47607	L440.11	CVA - cerebrovascular accident in the puerperium
47686	L181.00	Thyroid dysfunction in pregnancy/childbirth/puerperium
47741	L127000	Pre-eclampsia or eclampsia with hypertension unspecified
47863	Lyu5200	[X]Other single delivery by caesarean section
48500	Q49..00	Cardiovascular disorders originating in the perinatal period
49363	Q200100	Subdural haemorrhage unspecified, due to birth trauma
50093	L093000	Oliguria following abortive pregnancy
52875	L398000	Caesarean delivery unspecified
52967	Lyu0B00	[X]Complic following abortion & ectopic & molar preg, unspec
53141	L241.00	Tumour of uterine body in pregnancy/childbirth/puerperium
54652	L362z00	Secondary and delayed postpartum haemorrhage NOS
55304	L131z00	Hyperemesis gravidarum with metabolic disturbance NOS
56279	L440.12	Stroke in the puerperium
57236	L400200	Puerperal endometritis with postnatal complication
58156	L03y.00	Other ectopic pregnancy
58982	L186.00	Other cardiovascular diseases in pregnancy/childbirth/puerp
61204	L414z00	Postnatal deep vein thrombosis NOS
61578	L441000	Caesarean wound disruption unspecified
62052	L092500	Uterus damage following abortive pregnancy
62358	L167000	Liver disorder in pregnancy unspecified
62919	L125200	Severe pre-eclampsia - delivered with postnatal complication
63277	L393.00	Acute renal failure following labour and delivery
64127	L121000	Renal hypertension in pregnancy/childbirth/puerp unspecified
64384	L180z00	Diabetes mellitus in pregnancy/childbirth/puerperium NOS
66213	Q20yz12	Kidney injury due to birth trauma

For peer review only

66594	L186.11	Heart disease during pregnancy
67006	L096400	Pulmonary embolism following abortive pregnancy
68319	L351300	Rupture of uterus during/after labour with postnatal problem
70891	L126400	Eclampsia with postnatal complication
71314	L093.00	Renal failure following abortive pregnancy
71717	L121100	Renal hypertension in pregnancy/childbirth/puerp - delivered
72215	L241z00	Uterine body tumour in pregnancy/childbirth/puerperium NOS
72230	L241100	Tumour of uterine body - baby delivered
72458	L393000	Post-delivery acute renal failure unspecified
72513	7F13200	Extraperitoneal caesarean section
73407	L261200	Rhesus isoimmunisation with antenatal problem
73617	L261000	Rhesus isoimmunisation unspecified
73647	L188000	Abnormal GTT - unspec whether during pregnancy/puerperium
86756	Qyu3600	[X]Other chronic resp diseases originating/perinatal period
93710	Q317y00	Other specified perinatal chronic respiratory disease
94718	L121z00	Renal hypertension in pregnancy/childbirth/puerperium NOS
97367	L43z100	Obstetric pulmonary embolism NOS - delivered
99188	L173.00	Maternal tuberculosis in pregnancy/childbirth/puerperium
103465	Qyu3B00	[X]Cardiovasc disord origin in the perinat period, unspecif
103677	Eu32B00	[X]Antenatal depression
110868	L181000	Thyroid dysfunction - unspec whether in pregnancy/puerperium
111574	L114z00	Antepartum haemorrhage with trauma NOS

For peer review only

Appendix 8: Antenatal Read codes identified as pregnancy advice codes

medcode	read_oxmis_code	read_oxmis_term
30351	67A6.00	Drugs in pregnancy advice
36903	67AZ.00	Pregnancy advice NOS
102359	67AF.00	Pregnancy advice for patients with epilepsy
107892	67Iu.00	Advice on risk harm to fetus from maternl medictn dur preg
110888	67It.00	Advice on risk harm to mother from maternl medictn dur preg

For peer review only

startsource	Data source used to estimate pregnancy start date: 1 = Imputed[2], 2 = EDD, 3 = LMP, 4 = Gestational age at birth, 5 = Gestational age from antenatal record, 6 = EDC
startadj	Flag to indicate whether the pregnancy start date has been adjusted: 0 = Not adjusted, 1 = Due to antenatal records in the preceding 4 weeks, 2 = Due to specific conflicts between the estimated pregnancy duration and records indicating gestational age at birth (live births and stillbirths only), 3 = Both
Secondtrim[3]	Estimated start date of second trimester
Thirdtrim[3]	Estimated start date of third trimester
pregend	Estimated end date of pregnancy. NB: For pregnancies with unknown outcome, the date of the latest antenatal record in the pregnancy episode is provided.
endsource	Data source used to estimate pregnancy end date: 1 = Delivery record, 2 = Postnatal record in the mother's medical record, 3 = Discharge date relating to a delivery, 4 = Baby's (month and) year of birth as recorded in the baby's medical record, 5 = Postnatal record in the baby's medical record, 6 = First consultation in the baby's medical record. Only completed for live births and stillbirths.
endadj	Flag to indicate whether the pregnancy end date has been adjusted: 0 = Not adjusted, 1 = Due to specific conflicts between the estimated pregnancy duration and records indicating gestational age, 2 = Due to prior adjustments to the start date, 3 = Both. Missing for deliveries based on late pregnancy records[4].
gestdays	Estimated duration of pregnancy episode in days (calculated as pregend minus pregstart)

For peer review only

matage	Mother's age at end of pregnancy (years)
outcome	Outcome of pregnancy: 1 = Live birth, 2 = Stillbirth, 3 = 1 and 2, 4 = Miscarriage, 5 = TOP, 6 = Probable TOP, 7 = Ectopic, 8 = Molar, 9 = Blighted ovum, 10 = Unspecified loss, 11 = Delivery based on a third trimester pregnancy record, 12 = Delivery based on a late pregnancy record[4], 13 = Outcome unknown
preterm_ev	Flag to indicate evidence of a premature delivery: 1=preterm, 0=no evidence of preterm, 9=not applicable (outcome not a delivery)
postterm_ev	Flag to indicate evidence of a post-term delivery: 1=post-term, 0=no evidence of post-term, 9=not applicable (outcome not a delivery)
multiple_ev	Flag to indicate evidence of a multiple pregnancy: 1=multiple, 0=no evidence of multiple. Missing for pregnancy losses.
conflict	Flag to indicate whether the pregnancy episode overlaps with another episode (within a woman): 1=conflicting, 0= non-conflicting

1 A single babypatid is provided. For multiple pregnancies resulting in >1 liveborn infant (when mblbabies>1), additional babypatids may be retrieved from the MBL.

2 For "Outcome unknown" pregnancies, the imputed start date is obtained by subtracting 4 weeks from the earliest antenatal record in the episode.

3 The timing of trimesters is estimated using a common convention: first trimester (first day of LMP [pregstart] to 13 completed weeks), second (weeks 14 to 26), and third (week 27 to delivery [pregend]).

4 Late pregnancy records refer to the period up to 3 weeks before delivery, e.g. "Baby overdue".

For peer review only

Appendix 9: Read codes potentially misclassified as antenatal rather than outcomes

medcode	read_oxmis_code	read_oxmis_term
424	L281.00	Premature rupture of membranes
906	L100.00	Threatened abortion
1413	L264.00	Intrauterine death
1737	L02..00	Missed abortion
1879	L071.00	Unspecified abortion incomplete
3004	L14..11	Premature labour
6730	L051.12	Surgical abortion - incomplete
7114	L044.00	Inevitable abortion incomplete
7413	L041.00	Spontaneous abortion incomplete
8076	8H7W.00	Refer to TOP counselling
8147	L264.11	Fetal death in utero
8173	L043.00	Inevitable abortion unspecified
12241	L02..11	Missed miscarriage
12337	L051.00	Legal abortion incomplete
17625	L044.11	Inevitable miscarriage incomp
20621	ZV25313	[V]Admission for termination of pregnancy
20809	L14..00	Early or threatened labour
20933	6776	Preg. termination counselling
25883	L071y00	Unspecified incomplete abortion + no mention of complication
28605	L051z00	Incomplete legal abortion NOS
29439	L041z00	Incomplete spontaneous abortion NOS
33964	L0A4.00	Failed medical abortion, without complication
35184	L071z00	Unspecified incomplete abortion NOS
35273	L097.00	Readmission for abortive pregnancy (NHS codes)
35701	L100000	Threatened abortion unspecified
37831	L264z00	Intrauterine death NOS

For peer review only

39754	L051.11	Medal abortion - incomplete
41118	L08z.00	Failed attempted abortion NOS
41783	L041100	Incomp spontaneous abortion + delayed/excessive haemorrhage
47376	L0A1.00	Failed medical abortion complic by genital tract/pelvic infn
47435	L097200	Readmission for retained produc of concept, illegal abortion
50903	L0A2.00	Failed medical abortion comp by delayed/excessive haem'ge
53201	ZV25B00	[V]Admission for administration of abortifacient
59572	L0A3.00	Failed medical abortion, complicated by embolism
59789	L14z.00	Early or threatened labour NOS
65716	Q011.00	Fetus/neonate affected maternal premature rupture membrane
68683	7E0B.00	Introduction of abortifacient into uterine cavity
96418	L06z.00	Illegally induced abortion NOS
97391	L281200	Premature rupture of membranes with antenatal problem
99205	7E0Bz00	Introduction of abortifacient into uterine cavity NOS
101959	7E0B300	Extraamniotic injection of abortifacient NEC
102362	389B.00	Assessment for termination of pregnancy
102494	8Hh3.00	Self referral to termination of pregnancy service
105048	7E0By00	Introduction of abortifacient into uterine cavity OS

For peer review only

Appendix 10: Outcome Groupings

Pregnancy Outcomes will be grouped together with those pregnancies which would have similar rules applied and combinations of outcome group for each pair will be coded.

Group	*Pregnancy Register codes*	*Group*
Early Pregnancy Loss	4, 5, 6, 10, 7, 8, 9	1
Delivery	1, 2, 3, 11, 12	2
Unknown Outcome	13	3

For peer review only

Appendix 11: Read Codes identified as likely to only be recorded during current pregnancy

medcode	read_oxmis_term
30979	[SO]Fetus
36441	[V]Amniocentesis to screen for chromosomal anomalies
61455	[V]Amniotic fluid to screen for alphafetoprotein levels
6298	[V]Antenatal screening
49665	[V]Antenatal screening for chromosomal anomalies
35912	[V]Pregnancy confirmed
43428	[V]Screening for fetal growth retardation using ultrasonics
103341	[V]Screening for isoimmunisation
7536	[V]Screening for malformations using ultrasonics
13167	A/N 12 weeks examination
13166	A/N 16 week examination
29364	A/N 20 week examination
13169	A/N 24 week examination
26554	A/N 28 week examination
29627	A/N 30 week examination
13171	A/N 32 week examination
13170	A/N 34 week examination
29727	A/N 35 week examination
29610	A/N 36 week examination
26552	A/N 37 week examination
26553	A/N 38 week examination
26551	A/N 39 week examination
29280	A/N 40 week examination
37029	A/N 41 week examination
55605	A/N 42 week examination
3517	A/N booking examination
13984	Antenatal ultrasound confirms ectopic pregnancy
12260	A/N Rh antibody screen

For peer review only

68089	A/N Rh antibody screen NOS
70616	A/N sickle cell screen done
102099	A/N sickle cell screen NOS
64141	A/N syphilis screen-blood sent
14086	A/N U/S scan abnormal
27057	A/N U/S scan for ? abnormality
64537	A/N U/S scan for slow growth
37221	A/N U/S scan normal +? dates
35826	A/N U/S scan normal += dates
106588	Antenatal 22 week examination
106923	Antenatal 25 week examination
106425	Antenatal 31 week examination
13168	Antenatal examination NOS
10056	Antenatal examinations
13416	Antenatal sickle cell screen
13417	Antenatal syphilis screen
42326	Antenatal syphilis screen NOS
13968	Antenatal ultrasound confirms intra-uterine pregnancy
2029	Antenatal ultrasound scan
27056	Antenatal ultrasound scan at 17-22 weeks
39611	Antenatal ultrasound scan at 22-40 weeks
14084	Antenatal ultrasound scan at 9-16 weeks
14083	Antenatal ultrasound scan NOS
14085	Antenatal ultrasounds scan at 4-8 weeks
12890	Confirmation of pregnancy
50546	Dating scan
9462	Dating/booking US scan
100164	Detailed structural scan
103741	Doppler ultrasound scan of middle cerebral artery of fetus
102885	Doppler ultrasound scan of umbilical artery
95166	Doppler ultrasound scan of uterine artery
46126	Double test
13414	Downs screen - blood test

For peer review only

38358	Downs screen blood test abnormal
34508	Downs screen blood test normal
64832	Downs screening - blood sent
39173	Downs screening blood test NOS
103893	Fetal ascites scan
19720	Fetal monitoring
19590	Fetal movements felt
55493	Fetal movements seen
53420	Fetal tachycardia
9164	Fetal U-S scan
31110	Fundal height equal to dates
25875	Fundal height high for dates
37039	Fundal height low for dates
37038	Girth of pregnant abdomen
91773	Good baseline variability in fetal heart rate
105992	Height of uterine fundus
92171	Mid trimester scan
85992	Non routine obstetric scan for fetal observations
95875	Non routine obstetric scan for fetal observations NOS
38846	Normal fetal heart baseline pattern
13997	Nuchal scan
95881	O/E - fetal heart < 40
101119	O/E - fetal heart > 200
68996	O/E - fetal heart 100-120
26707	O/E - fetal heart 120-160
62903	O/E - fetal heart 160-180
62898	O/E - fetal heart 180-200
72837	O/E - fetal heart 40-80
70856	O/E - fetal heart 80-100
7681	O/E - fetal heart heard
22815	O/E - fetal movements
25153	O/E - fetal movements felt
52857	O/E - fetal movements NOS
53687	O/E - fetal movements seen

For peer review only

27801	O/E - fetal movemnt.diminished
26710	O/E - fetal presentation
67186	O/E - fetal presentation NOS
69819	O/E - fetal station NOS
24701	O/E - fetus very active
26708	O/E - fundal size = dates
37049	O/E - fundus = term size
26705	O/E - fundus 12-16 week size
37051	O/E - fundus 16-20 week size
26704	O/E - fundus 20-24 week size
26709	O/E - fundus 24-28 week size
30802	O/E - fundus 28-32 week size
30803	O/E - fundus 32-34 week size
26703	O/E - fundus 34-36 week size
26706	O/E - fundus 36-38 week size
13318	O/E - fundus size - obstetric
30804	O/E - gravid uterus size
62897	O/E - gravid uterus size NOS
37180	O/E - lie of fetus
29788	O/E - multiple presentation
63024	O/E -fetal presentation unsure
37050	O/E -fundus 38 weeks-term size
49519	Observation of position of pregnancy
12625	Obstetric monitoring
44173	Obstetric X-ray - fetus
56727	Obstetric X-ray - placenta
85951	Other non routine obstetric scan NOS
96343	Other specified routine obstetric scan
13165	Patient currently pregnant
127	Patient pregnant
14899	Patient pregnant NOS
38669	Placenta U-S scan
9986	Pregnancy care
4536	Pregnancy confirmed

For peer review only

15338	Pregnancy unplanned ? wanted
14877	Pregnant - ? planned
30817	Pregnant - blood test confirms
51298	Pregnant - on abdom. palpation
20240	Pregnant - planned
16215	Pregnant - urine test confirms
35592	Pregnant - V.E. confirms
10173	Pregnant abdomen observation
15567	Pregnant -unplanned-not wanted
107698	Pregnant uterus displaced laterally
32975	Pregnant, diaphragm failure
29692	Pregnant, IUD failure
14994	Pregnant, sheath failure
11989	Referral for termination of pregnancy
2278	Requests pregnancy termination
69815	Rh screen - 1st preg. sample
29623	Rh screen - 2nd preg. sample
109416	Rh screen - 3rd preg. sample
93946	Rhesus detailed scan
86011	Routine obstetric scan
85245	Routine obstetric scan NOS
6095	Seen in antenatal clinic
29205	Serum pregnancy test positive
70845	Sinusoidal pattern of fetal heart
27614	Triple test
39218	Ultrasonic doppler for fetal heart sounds
19800	Ultrasound in obstetric diagn.
12837	Ultrasound monitoring of early pregnancy
13965	Ultra-sound scan - obstetric
3030	Urine pregnancy test positive
2382	U-S obstetric diagn. scan NOS
29685	U-S obstetric scan abnormal
4797	U-S obstetric scan normal
45963	U-S scan - fetal abnormality

For peer review only

72159	U-S scan - fetal cephalometry
42093	U-S scan - fetal maturity
41919	U-S scan - fetal presentation
41937	U-S scan - multiple fetus
35558	U-S scan - obstetric, diagn.
68858	U-S scan -placental localisatn
67047	Viability scan
37147	Viability US scan
10306	Weeks pregnant

For peer review only

Appendix 12: Outcome Group Combinations

Within conflicting pairs combinations of outcome groups will be coded as follows:

Outcome Group combination	*Variable Code*
1 1 (Loss- Loss)	1
1 2 (Loss- Delivery)	2
1 3 (Loss- Unknown)	3
2 2 (Delivery- Delivery)	4
2 3 (Delivery- Unknown)	5
3 3 (Unknown- Unknown)	6

For peer review only

Appendix 13: Read codes for Antenatal scan

medcode	read_oxmis_code	Read term
2029	62G..00	Antenatal ultrasound scan
13965	584..13	Ultra-sound scan - obstetric
9462	584A.00	Dating/booking US scan
2382	584Z.00	U-S obstetric diagn. scan NOS
13997	584G.00	Nuchal scan
42093	5846	U-S scan - fetal maturity
37147	584B.00	Viability US scan
4797	5842	U-S obstetric scan normal
27019	5841	U-S obstetric scan requested
9164	584..11	Fetal U-S scan
14083	62GZ.00	Antenatal ultrasound scan NOS
35826	62G6.00	A/N U/S scan normal += dates
14084	62GC.00	Antenatal ultrasound scan at 9-16 weeks
35558	584..12	U-S scan - obstetric, diagn.
50546	7F26000	Dating scan
29012	7F27300	Nuchal translucency scan
27056	62GD.00	Antenatal ultrasound scan at 17-22 weeks
39611	62GE.00	Antenatal ultrasound scan at 22-40 weeks
47415	62G5.00	A/N U/S scan awaited
37220	62G2.00	A/N U/S scan offered
14085	62GB.00	Antenatal ultrasounds scan at 4-8 weeks
29685	5843	U-S obstetric scan abnormal
72159	5845	U-S scan - fetal cephalometry
45963	5847	U-S scan - fetal abnormality
27057	62G9.00	A/N U/S scan for ? abnormality

For peer review only

41919	5849	U-S scan - fetal presentation
30885	62G4.00	A/N U/S scan wanted
86011	7F26.00	Routine obstetric scan
68858	5844	U-S scan -placental localisatn
67047	7F26100	Viability scan
41937	5848	U-S scan - multiple fetus
14086	62G8.00	A/N U/S scan abnormal
85992	7F27.00	Non routine obstetric scan for fetal observations
37221	62G7.00	A/N U/S scan normal +? dates
38669	5844.11	Placenta U-S scan
78449	7F28.00	Other non routine obstetric scan
100164	7F27100	Detailed structural scan
92171	7F26200	Mid trimester scan
95166	7F2A111	Doppler ultrasound scan of uterine artery
64537	62GA.00	A/N U/S scan for slow growth
47116	7F28000	Placental localisation scan
85245	7F26z00	Routine obstetric scan NOS
102885	7F2A011	Doppler ultrasound scan of umbilical artery
96343	7F26y00	Other specified routine obstetric scan
95875	7F27z00	Non routine obstetric scan for fetal observations NOS
85951	7F28z00	Other non routine obstetric scan NOS
98261	7F27y00	OS non routine obstetric scan for fetal observations
95698	7F28y00	Other specified other non routine obstetric scan

For peer review only

Appendix 14: DID Snomed foetal scan codes

Dating/booking ultrasound scan (procedure)	169229007
Fetal anatomy study (procedure)	271442007
Fetal biophysical profile (procedure)	21623001
Fetal echocardiography (procedure)	433235006
Magnetic resonance imaging of multiple pregnancy (procedure)	450825001
Placental localization (procedure)	164817009
Ultrasonography of multiple pregnancy for fetal anomaly (procedure)	445866007
Ultrasonography of multiple pregnancy for fetal nuchal translucency (procedure)	446810002
Ultrasound scan for amniotic fluid volume (procedure)	241494004
Ultrasound scan for fetal growth (procedure)	241493005

For peer review only

Appendix 15: Number of episodes with a suitably timed outcome in linked HES data

Dataset in which evidence of a suitably timed pregnancy outcome was found.	N pregnancy episodes where evidence of an outcome was found (% of episodes which were eligible for this linked data source)	N pregnancy episodes which were during current registration and UTS follow up	Total number of pregnancy episodes with recorded outcome missing which were eligible for HES linkage to each source
HES Diagnosis (Part of HES APC)	24,902 (5.9%)	16,389 (65.8%)	424,375
HES Maternity (Part of HES APC)	163,483 (38.5%)	109,393 (66.9%)	424,375
HES Procedures (Part of HES APC)	201,731 (47.5%)	133,077 (66.0%)	424,375
HES Episodes (Part of HES APC)	185,436 (43.7%)	122,350 (66.0%)	424,375
HES Outpatient	735 (0.2%)	560 (76.2%)	311,982
Any HES Source	211,070 (49.7%)	139,084 (65.9%)	424,375

For peer review only

Appendix 16: Numbers of pregnancy episodes with recorded outcome missing which were within practice UTS follow-up and patient's current registration period that were consistent with applied criteria for each scenario

**Scenario**	**Description**	**N pregnancy episodes which meet this scenario (% of total episodes with missing outcome)**	**N pregnancy episodes which only meet this scenario (% of the total episodes with missing outcome)**	**N pregnancy episodes with evidence of an outcome in linked HES (% of linkage eligible episodes)**
Denominator		475,664	475,664	265,264
Problem 1: The women was pregnant at the time of the database record, but the outcome was not captured in CPRD primary care data.				
Scenario 1a	The pregnancy outcome occurred in hospital or elsewhere and information wasn't fed back to the practice.	139,084 (29.2%)	1,825 (0.4%)	139,084 (52.4%)
Scenario 1b	The outcome of the pregnancy is recorded in the primary care data but has no event date associated with it.	475 (0.1%)	28 (0.0%)	113 (0.0%)
Scenario 1c	The pregnancy occurred before the patient was registered at the practice or before UTS	-	-	-

Problem 2: The women was pregnant at the time of the database record, but the pregnancy was still ongoing at the end of available follow up in the database.				
Scenario 2a	The patient transferred out before the putative end of pregnancy	117,571 (24.7%)	34,659 (7.3%)	52,601 (19.8%)
Scenario 2b	The last collection date of the practice was before the putative end of pregnancy	58,698 (12.3%)	20,122 (4.2%)	21,702 (8.2%)
Problem 3: The patient was not pregnant at the time of the database record.				
Scenario 3a	Episode is derived from historical pregnancy information recorded in the first few months after the patient joined the practice	3,875 (0.8%)	386 (0.1%)	1,271 (0.5%)
Scenario 3b	Patient asks for advice whilst planning a pregnancy	0 (0.0%)	0 (0.0%)	0 (0.0%)
Problem 4: The pregnancy record belongs to another pregnancy episode in the Register.				
Scenario 4a	Delay in recording the outcome of a pregnancy, algorithm calculates LMP too late and uncovers records at the beginning of pregnancy creating this PWO.	35,255 (7.4%)	8,265 (1.7%)	14,402 (5.4%)

For peer review only

Scenario 4b	The LMP is derived from the data and is wrong resulting in early codes being uncovered creating this episode	17,110 (3.6%)	3,715 (0.8%)	6,651 (2.5%)
Scenario 4c	The LMP has been shifted backwards uncovering records at the end of the pregnancy	0 (0.0%)	0 (0.0%)	0 (0.0%)
Scenario 4d	A code recorded relating to the patient's delivery history is incorrectly identified by the algorithm as a delivery uncovering records at the end.	219,505 (46.1%)	109,161 (22.9%)	65,883 (24.8%)
Scenario 4e	The outcome of the pregnancy episode has been misclassified as antenatal	18,222 (3.8%)	7,418 (1.6%)	3,990 (1.5%)
Pregnancy Episodes which didn't meet any scenario	These pregnancy episodes did not meet the criteria for any identified scenarios.	94,769 (19.9%)	0 (0.0%)	0 (0.0%)

For peer review only

Appendix 17: Numbers of conflicting pregnancy episodes which were within practice UTS follow-up and patient's current registration period that were consistent with applied criteria for each scenario

Scenario	Description	N pregnancy pairs (% of total conflicting pregnancy pairs)	N which only fit this scenario (% of the total pairs meeting this scenario)	N of pairs with a linked baby in the MBL (% of the total pairs meeting this scenario)	N pairs with evidence of pregnancy in linked HES
Denominator		144,670	144,670	144,670	93,100
Problem 1: Both pregnancies are true but one is a current pregnancy and one is a historical pregnancy					
Scenario 1a	The GP records a past delivery or loss during a current pregnancy with the same outcome resulting in another episode being created	1,981 (1.4%)	317 (0.2%)	1,782 (1.2%)	1,875 (2.0%)
Scenario 1b	A patient has a record relating to a loss recorded during a pregnancy ending in delivery or vice-versa. Conflicting episodes are generated by the algorithm	31,526 (21.8%)	15,453 (10.7%)	8,275 (5.7%)	11,410 (12.3%)
Problem 2: Both pregnancies are historical					

For peer review only - http://bmjopen.bmj.com/site/about/guidelines.xhtml

Scenario 2a	A patient has information on historical pregnancies recorded with the current date rather than the actual date.	12,557 (8.7%)	0 (0.0%)	97 (0.1%)	4,309 (4.6%)
Problem 3: Both pregnancies are true and current but the gestation of the second pregnancy estimated by the algorithm is too long.					
Scenario 3a	The woman has two losses which are >8weeks and <12weeks apart.	2,284 (1.6%)	3 (0.0%)	0 (0.0%)	635 (0.7%)
Scenario 3b	The woman has two pregnancies close together and the second ends in delivery. If the LMP information is wrong for this pregnancy, then algorithm episodes may overlap.	13,464 (9.3%)	2,387 (1.6%)	1,113 (0.8%)	4,502 (4.8%)
Problem 4: : The pregnancy is true and current but is split into separate episodes by the rules of the algorithm					
Scenario 4a	The GP records further information about a pregnancy outcome >25 weeks later for deliveries or >8weeks <12 weeks later for losses.	2,347 (1.6%)	183 (0.1%)	2,155 (1.5%)	2,255 (2.4%)

For peer review only

Scenario 4b	The GP records further antenatal information after the end of a pregnancy. Conflicting episodes are generated by the algorithm	27,131 (18.8%)	25,097 (17.3%)	11,097 (7.7%)	11,668 (12.5%)
Scenario 4c	The patient has a follow up scan after a pregnancy loss. The scan is recorded in the data as an antenatal scan, a conflicting episode is then generated by the algorithm.	2,088 (1.4%)	0 (0.0%)	0 (0.0%)	587 (0.6%)
Scenario 4d	The GP records information about a pregnancy but no outcome with >6 weeks between records. If the second episode has gestational information the start may be assigned before the start of the first episode.	9,911 (6.9%)	9,911 (6.9%)	0 (0.0%)	5,079 (5.5%)
Scenario 4e	The pregnancy dates have been shifted backwards by the rules of the algorithm leaving uncovered records. Conflicting episodes are generated by the algorithm.	55,205 (38.2%)	53,044 (36.7%)	43,945 (30.4%)	33,057 (35.5%)
None	These pairs of pregnancies did not meet the criteria for any identified scenarios.	15,650 (10.8%)	-	8,921 (6.2%)	8,235 (8.8%)

For peer review only

Appendix 18: Number of conflicting episode pairs by outcome combination

Outcome Combination	N pairs (% of total conflicting pairs)
two losses	65,826 (26.2%)
one loss one delivery	73,222 (29.2%)
one loss one unknown	62,776 (25.0%)
two deliveries	10,204 (4.1%)
one delivery one unknown	24,303 (9.7%)
two unknowns	14,695 (5.9%)
Total Pairs	251,026 (100%)

For peer review only

The RECORD statement – checklist of items, extended from the STROBE statement, that should be reported in observational studies using routinely collected health data.

	Item No.	STROBE items	Location in manuscript where items are reported	RECORD items	Location in manuscript where items are reported
**Title and abstract**					
	1	(a) Indicate the study's design with a commonly used term in the title or the abstract (b) Provide in the abstract an informative and balanced summary of what was done and what was found		RECORD 1.1: The type of data used should be specified in the title or abstract. When possible, the name of the databases used should be included.<br><br>RECORD 1.2: If applicable, the geographic region and timeframe within which the study took place should be reported in the title or abstract.<br><br>RECORD 1.3: If linkage between databases was conducted for the study, this should be clearly stated in the title or abstract.	Reported in the title (page 1, line 2)<br><br>Reported in the abstract setting and participants sections (page 1, line 22-26)<br><br>Reported in the design section of the abstract (page 1, line 30)
**Introduction**					
Background rationale	2	Explain the scientific background and rationale for the investigation being reported			Introduction pages 2-3
Objectives	3	State specific objectives, including any prespecified hypotheses			Page 3 line 102-108
**Methods**					
Study Design	4	Present key elements of study design early in the paper			See section "Identifying scenarios to explain the occurrence of

For peer review only

					uncertain episodes" pages 4 and 5, line 165-189
Setting	5	Describe the setting, locations, and relevant dates, including periods of recruitment, exposure, follow-up, and data collection			Data Sources section pages 3 and 4, line 113-151
Participants	6	*(a) Cohort study* - Give the eligibility criteria, and the sources and methods of selection of participants. Describe methods of follow-up<br>*Case-control study* - Give the eligibility criteria, and the sources and methods of case ascertainment and control selection. Give the rationale for the choice of cases and controls<br>*Cross-sectional study* - Give the eligibility criteria, and the sources and methods of selection of participants<br><br>*(b) Cohort study* - For matched studies, give matching criteria and number of exposed and unexposed<br>*Case-control study* - For matched studies, give matching criteria and the number of controls per case		RECORD 6.1: The methods of study population selection (such as codes or algorithms used to identify subjects) should be listed in detail. If this is not possible, an explanation should be provided.<br><br>RECORD 6.2: Any validation studies of the codes or algorithms used to select the population should be referenced. If validation was conducted for this study and not published elsewhere, detailed methods and results should be provided.<br><br>RECORD 6.3: If the study involved linkage of databases, consider use of a flow diagram or other graphical display to demonstrate the data linkage process, including the number of individuals with linked data at each stage.	Study Population page 4, line 156-163<br><br>The Pregnancy Register algorithm is detailed in Figure 1 and the publication which details it's methodology and validation is referenced throughout the paper (Reference 3 page 36, line 470)
Variables	7	Clearly define all outcomes, exposures, predictors, potential confounders, and effect modifiers. Give diagnostic criteria, if applicable.		RECORD 7.1: A complete list of codes and algorithms used to classify exposures, outcomes, confounders, and effect modifiers should be provided. If these cannot be reported, an explanation should be provided.	Full code lists are provided in the supplementary material.

For peer review only - http://bmjopen.bmj.com/site/about/guidelines.xhtml

Data sources/ measurement	8	For each variable of interest, give sources of data and details of methods of assessment (measurement). Describe comparability of assessment methods if there is more than one group			Page 5 line 167- to page 18 line 204
Bias	9	Describe any efforts to address potential sources of bias			N/A This study is not standard epidemiological design, it is methodological designed to help inform future studies.
Study size	10	Explain how the study size was arrived at			Page 4 line 158
Quantitative variables	11	Explain how quantitative variables were handled in the analyses. If applicable, describe which groupings were chosen, and why			N/A This study is not standard epidemiological design, it is methodological designed to help inform future studies.
Statistical methods	12	(a) Describe all statistical methods, including those used to control for confounding<br>(b) Describe any methods used to examine subgroups and interactions<br>(c) Explain how missing data were addressed<br>(d) *Cohort study* - If applicable, explain how loss to follow-up was addressed<br>*Case-control study* - If applicable, explain how			N/A This study is not standard epidemiological design, it is methodological designed to help inform future studies.

For peer review only

		matching of cases and controls was addressed<br>*Cross-sectional study* - If applicable, describe analytical methods taking account of sampling strategy<br>(e) Describe any sensitivity analyses			
Data access and cleaning methods		..		RECORD 12.1: Authors should describe the extent to which the investigators had access to the database population used to create the study population.<br><br>RECORD 12.2: Authors should provide information on the data cleaning methods used in the study.	No data cleaning was applied beyond restricting to UTS follow-up described P3 line 125 and appendix 1.
Linkage		..		RECORD 12.3: State whether the study included person-level, institutional-level, or other data linkage across two or more databases. The methods of linkage and methods of linkage quality evaluation should be provided.	Routine rather than study specific linkages were used. Details are provided on page 4 and page 19
**Results**					
Participants	13	(a) Report the numbers of individuals at each stage of the study (*e.g.*, numbers potentially eligible, examined for eligibility, confirmed eligible, included in the study, completing follow-up, and analysed)<br>(b) Give reasons for non-participation at each stage.		RECORD 13.1: Describe in detail the selection of the persons included in the study (*i.e.,* study population selection) including filtering based on data quality, data availability and linkage. The selection of included persons can be described in the text and/or by means of the study flow diagram.	Page 21 baseline table of study participants and pregnancy episodes provided.

For peer review only

		(c) Consider use of a flow diagram			
Descriptive data	14	(a) Give characteristics of study participants (*e.g.*, demographic, clinical, social) and information on exposures and potential confounders<br>(b) Indicate the number of participants with missing data for each variable of interest<br>(c) *Cohort study* - summarise follow-up time (*e.g.*, average and total amount)			Page 21 baseline table of study participants and pregnancy episodes provided
Outcome data	15	*Cohort study* - Report numbers of outcome events or summary measures over time<br>*Case-control study* - Report numbers in each exposure category, or summary measures of exposure<br>*Cross-sectional study* - Report numbers of outcome events or summary measures			This study is not standard epidemiological design, it is methodological designed to help inform future studies.
Main results	16	(a) Give unadjusted estimates and, if applicable, confounder-adjusted estimates and their precision (e.g., 95% confidence interval). Make clear which confounders were adjusted for and why they were included<br>(b) Report category boundaries when continuous variables were categorized<br>(c) If relevant, consider translating estimates of relative risk into absolute risk for a meaningful time period			Page 20-28

For peer review only

Other analyses	17	Report other analyses done—e.g., analyses of subgroups and interactions, and sensitivity analyses			Appendix 16 and 17.
**Discussion**					
Key results	18	Summarise key results with reference to study objectives			Page 29-30
Limitations	19	Discuss limitations of the study, taking into account sources of potential bias or imprecision. Discuss both direction and magnitude of any potential bias		RECORD 19.1: Discuss the implications of using data that were not created or collected to answer the specific research question(s). Include discussion of misclassification bias, unmeasured confounding, missing data, and changing eligibility over time, as they pertain to the study being reported.	Limitations discussed on Page 30 Lines 399-413
Interpretation	20	Give a cautious overall interpretation of results considering objectives, limitations, multiplicity of analyses, results from similar studies, and other relevant evidence			Page 29 lines 329-396
Generalisability	21	Discuss the generalisability (external validity) of the study results			Page 29 line 338
**Other Information**					
Funding	22	Give the source of funding and the role of the funders for the present study and, if applicable, for the original study on which the present article is based			Page 33 line 446
Accessibility of protocol, raw data, and programming code		..		RECORD 22.1: Authors should provide information on how to access any supplemental information such as the study protocol, raw data, or programming code.	Data sharing statement page 34 line 458

For peer review only

*Reference: Benchimol EI, Smeeth L, Guttmann A, Harron K, Moher D, Petersen I, Sørensen HT, von Elm E, Langan SM, the RECORD Working Committee. The REporting of studies Conducted using Observational Routinely-collected health Data (RECORD) Statement. *PLoS Medicine* 2015; in press.

*Checklist is protected under Creative Commons Attribution (CC BY) license.

For peer review only
